# High five! Methyl probes at five ring positions of phenylalanine explore the hydrophobic core dynamics of zinc finger miniproteins†

**DOI:** 10.1039/d1sc02346b

**Published:** 2021-07-24

**Authors:** Philip Horx, Armin Geyer

**Affiliations:** Philipps-University Marburg 35043 Marburg Germany geyer@staff.uni-marburg.de

## Abstract

The elucidation of internal dynamics in proteins is essential for the understanding of their stability and functionality. Breaking the symmetry of the degenerate rotation of the phenyl side chain provides additional structural information and allows a detailed description of the dynamics. Based on this concept, we propose a combination of synthetic and computational methods, to study the rotational mobility of the Phe ring in a sensitive zinc finger motif. The systematic methyl hopping around the phenylalanine ring yields *o*-, *m*-, *p*-tolyl and xylyl side chains that provide a vast array of additional NOE contacts, allowing the precise determination of the orientation of the aromatic ring. MD simulations and metadynamics complement these findings and facilitate the generation of free energy profiles for each derivative. Previous studies used a wide temperature window in combination with NMR spectroscopy to elucidate the side chain mobility of stable proteins. The zinc finger moiety exhibits a limited thermodynamic stability in a temperature range of only 40 K, making this approach impractical for this compound class. Therefore, we have developed a method that can be applied even to thermolabile systems and facilitates the detailed investigation of protein dynamics.

## Abstract 2

Breaking the symmetry of the degenerate rotation of the phenylalanine side chain provides information about the side chain dynamics in a zinc finger motif.

## Graphical abstract

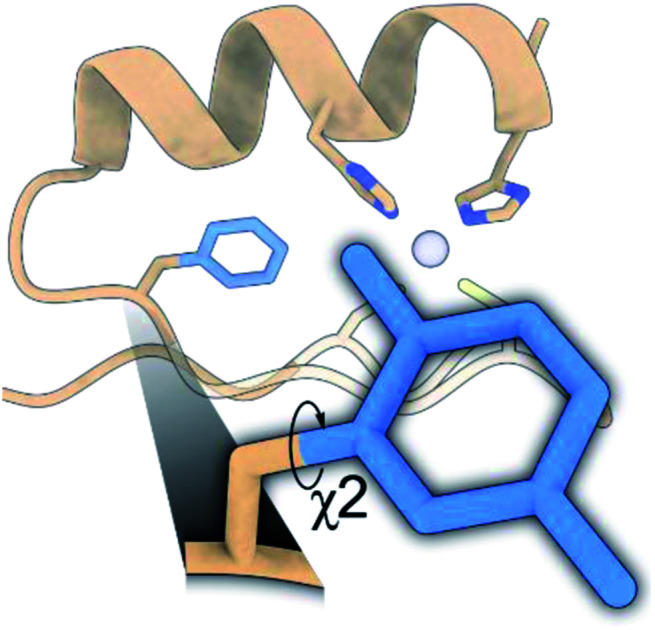

## Introduction

Side chain rotations make protein structures dynamic so they can fulfill their functional duties like molecular recognition, catalysis, and signal transmission to name a few examples. Different spectroscopic and computational methods identify mobilities on different timescales with the aim of correlating structural dynamics with functional properties.^1,2^ The application of the model free quantification of signal coalescence for the determination of aromatic side chain mobility in BPTI by Wüthrich *et al.*^3,4^ was an early hallmark in the characterization of protein dynamics. While the general idea of studying dynamics by applying temperature gradients is still used today, its application is limited to only the most stable proteins and hampered by the number of aromatic NMR signals.^5^ A further development is the use of relaxation dispersion experiments which provided excellent agreement with simulations^6^ and allows the study of near degenerate spin systems.^7–10^ Here, we propose a method using phenylalanine derivatives with modified side chains in combination with molecular dynamics simulation, to characterize the hydrophobic core dynamics in miniproteins with limited thermodynamic stability.

**Fig. 1:**
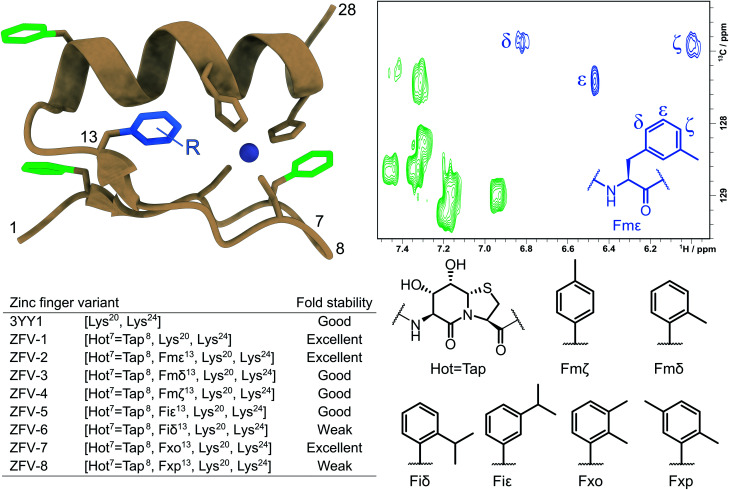
Tertiary structure of the third zinc finger of Yin Yang 1 protein (3YY1), based on the crystal structure^30^ showing the side chains involved in zinc binding and the four phenylalanines in a color code. The solution structure exhibits the same ββα fold^15^ with fast aromatic flips, causing an AA′BB′C spin system in the ^1^H NMR of all four Phe. The central aromatic residue Phe^13^, shown in blue in the HSQC expansion above right, stands out because of its large chemical shift dispersion. The zinc finger variants (ZFVs) in the inset table differ by their methylation pattern of Phe^13^. The amino acid code identifies the position of the methyl groups from the Greek letters. Hot=Tap stands for the hydroxythreonine annulated at thiaproline according to lit.^31^ The arginine residues on positions 20 and 24 were substituted with lysine to obtain higher solubility. Every zinc finger variant is listed in this table with the corresponding mutations and fold stability, qualitatively determined by temperature dependent ^1^H NMR spectroscopy (ESI†).

Miniproteins are the smallest cooperative functional polypeptide fold.^11^ One prominent example is zinc finger peptides that contain a β-turn, antiparallel β-sheet, α-helix, and loop, all tightly packed around a hydrophobic core.^12,13^ In the early 90's, investigations focused on illuminating structural features and motions profiting from the straightforward synthesis of standalone hairpin folds and the accessibility for theoretical studies.^14–17^ One structural element that is essential for a stable fold is the hydrophobic core, which consists of several aliphatic amino acids, often accompanied by aromatic side chains. The importance of this structural feature for the stability of zinc finger peptides has been under investigations multiple times throughout the literature using various spectroscopic methods and mutation experiments.^18–21^ It could be shown that especially the central phenylalanine on the position 10 or 13, depending on the size of the zinc binding site, is of great importance for a stable tertiary fold. ^1^H NMR spectroscopy identifies exceptionally large signal dispersion for the central aromatic amino acid F13 of the hydrophobic core and is clearly separated from the rest with a strongly shielded ζ-proton, due to edge-to-face interaction with His (Fig. 1). It was noted that even though the central F13 is packed tightly in the hydrophobic core, an AA′BB′X spin system resulting from fast conformational averaging was observed for this side chain.^22^ Relaxation measurements concluded on restricted rotational motion around *χ*^2^ whereas mutagenesis experiments highlighted the intolerance of the hydrophobic core against non-aromatic substitutions.^18,21,23^ This highly conserved F13 was substituted by Lachenmann *et al.* with a cyclohexyl amino acid with the aim to differentiate π-stacking from other interactions within the hydrophobic core.^21^ They discovered that the side chain volume is of importance rather than aromaticity. Building on this, further substitutions on other positions have been performed, to obtain more stable zinc finger derivatives and further elucidate the structure and functions. They range from single side mutations to precisely tuned DNA-interactions^24,25^ to multiple non-standard residues, which improve stability while retaining the fold.^26^ Lombardo *et al.* were even able to completely swap the α-helix of the zinc finger peptide with an oligourea helix and preserve metal binding properties.^27^ Beyond side chain substitutions, other changes were studied in zinc fingers too, although care must be taken in the region of the β-hairpin turn to avoid the loss of the affinity for zinc in its tetrahedral coordination sphere.^26^ Cys-X_4_-Cys type zinc fingers, such as the third finger of YY1 or the second finger of Sp1 studied here are known to tolerate significant changes in the β-turn region while adopting a stable and strong fold.^28,29^ We chose the third zinc finger of Yin Yang1, since the system has been well studied and provides an ideal model for miniproteins.^28,30^

Here, we introduce strongly β-turn stabilizing Hot=Tap^31^ which has been incorporated into several β-turn motifs^32,33^ and displays superior conformational restriction of hairpin turns, in comparison to BTD.^34^ After the substitution of the natural β-turn, we observed an increased yield, crude purity, and solubility for the zinc finger derivative, while retaining the fold of the natural zinc finer peptide and the BTD derivative. As expected for a well-folded structure, the influence of the stronger restriction by Hot=Tap is nearly indistinguishable from BTD which lacks preferred lactam puckering and β-branching. With this stable zinc finger fold at hand, we then stepwise increased the steric demand of the aromatic side chain at position Phe^13^. The combined effects reduce the flipping frequency of the Phe^13^ aromatic side chain and characterize the often underestimated contributions of dynamic modes to the fold stability of the miniprotein. Fig. 1 shows the systematic methyl-hopping on the aromatic ring of Phe^13^ with the aim to identify structures with less time-averaged NOEs resulting from the symmetry-broken side chain. This additional structural information about the rotational flexibility is then used to analyze the entropic contribution of phenyl groups to the overall fold stability. The classical Cys_2_–His_2_ zinc finger fold allows the aromatic residue to rotate freely around its *χ*^2^ dihedral. Because of this fast exchange only ambiguous information is available about the orientation of the ring. The tolyl and xylyl amino acids of Fig. 1 deliver a characteristic set of NOE contacts, to precisely determine the orientation (*χ*^1^) and the rotational mobility (*χ*^2^) of the aromatic side chain. The additional structural information facilitated the calculations of NMR structures to further visualize the ββα motif and aid in the understanding of geometrical requirements. Nevertheless, NMR structure ensembles are still limited in quantifying the internal motions of peptides. To overcome this challenge researchers are using molecular dynamics simulation to further investigate those fluctuations.^35^ For freely accessible side chains, those motions are on a fast timescale and thus can be easily addressed. After the formation of a well-defined hydrophobic core, rotations can slow down immensely and thus are out of reach for standard MD. To address this challenge, enhanced sampling methods like replica exchange MD,^36^ steered MD,^37^ Gaussian accelerated MD^38^ and metadynamics^39^ have been developed. Metadynamics is a widely used method for the reduction of dimensions in which one (or a few) specific collective variable (reaction coordinates) is selected and a positive Gaussian potential is employed at a certain timestep.^40–42^ This bias increases sampling and furthermore allows the creation of a free energy surface along the sampled coordinate. These combined advantages, for us, make metadynamics the method of choice to investigate the rotation of modified phenylalanine residues inside the hydrophobic core.

## Results and discussion

**Fig. 2:**
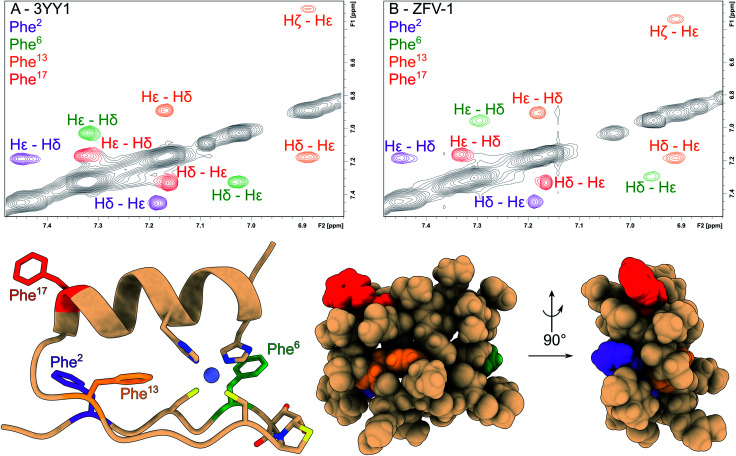
The aromatic region of the phenylalanine residues has been magnified and color coded in the NOESY spectra of 3YY1 (A) and ZFV-1 (B). All phenylalanine moieties show two signals for Hδ and Hε; while the Hζ from Phe^2^, Phe^6^ and Phe^17^ falls into a very narrow chemical shift range. Only Phe^13^ splits into 3 separate signals. Besides small changes in chemical shift values for Phe^6^, no major changes in the aromatic region could be observed upon the introduction of Hot=Tap. Below, the molecular representation of ZFV-1 with the Phe moieties color coded to the correspondent NOESY spectra and Hot=Tap is explicitly shown.

We started our investigation by synthesizing the native third zinc finger of YY1, which we denote in the manuscript as 3YY1, using Fmoc solid phase peptide synthesis, as previously reported by Viles *et al.*^28^ After purification with semipreparative RP-HPLC and addition of zinc to an aqueous solution, the peptide exhibits the, for a Cys_2_–His_2_ zinc finger common, ββα fold. The substitution of Glu^7^ and Gly^8^ with Hot=Tap, a dipeptide β-turn mimic, developed in our group^31^ results in the same fold with only small changes in chemical shift values. This mimic shows strong resemblance to the well-established BTD^43^ yet is superior in the rigidification of the β-turn, as BTD lacks preferred lactam puckering and β-branching.^31^ The hydroxy groups are liberated from the acetonide during acidic cleavage from the resin.^34^ After successful synthesis we observed higher purity, both crude and purified (ESI†), higher yield and better solubility. NMR analysis of the apo peptide reveals that the Hot=Tap derivative exhibits a pre-folded species with β-turn indications. 2D-NMR structural analysis after the addition of zinc reveals the same TOCSY/NOE pattern as observed in the parent peptide, which indicates the ββα fold for both peptides which is most obvious from the identity of the aromatic chemical shifts of the four phenylalanines well-distributed over the entire zinc finger fold (Fig. 2) of 3YY1. All four show AA'BB'C spin systems caused by fast rotational averaging. Due to signal overlap, only δ and ε protons are clearly distinguishable. Only Phe^13^, which is part of the hydrophobic core, situated between both secondary structure elements, shows three clearly resolved signals even at lower temperatures.

With the aim of obtaining more information about the rotational behavior, we performed molecular dynamics simulations to accurately model an atomistic representation of zinc finger proteins and to supplement the experimental investigations. Researchers in the past few decades have employed MD simulations in various studies to examine various challenges, like folding/unfolding or DNA-binding mechanisms.^44–47^ We simulated each zinc finger peptide for 1.5 μs and compared the flexibility of the *χ*^2^-dihedral between the phenylalanine residues.

**Fig. 3:**
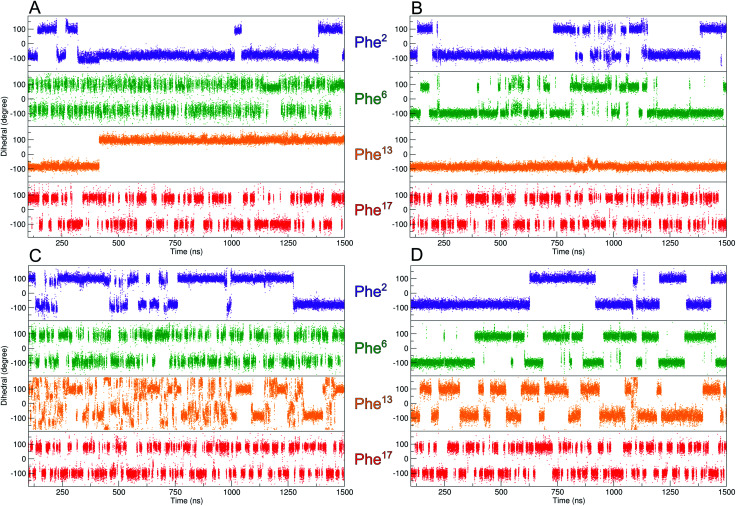
Evolution of the *χ*^2^-dihedral orientation of the four Phe residues over the duration of the MD-simulation. (A) 3YY1 zinc finger peptide and (B) ZFV-1. Since the Phe in the hydrophobic core displays restricted mobility, well-tempered metadynamics simulations of 3YY1 and ZFV-1 have been performed (C and D) biasing the *χ*^2^-dihedral of Phe^13^. The filling of the minima does not affect other mobilities of ZFV-1.

**Fig. 4:**
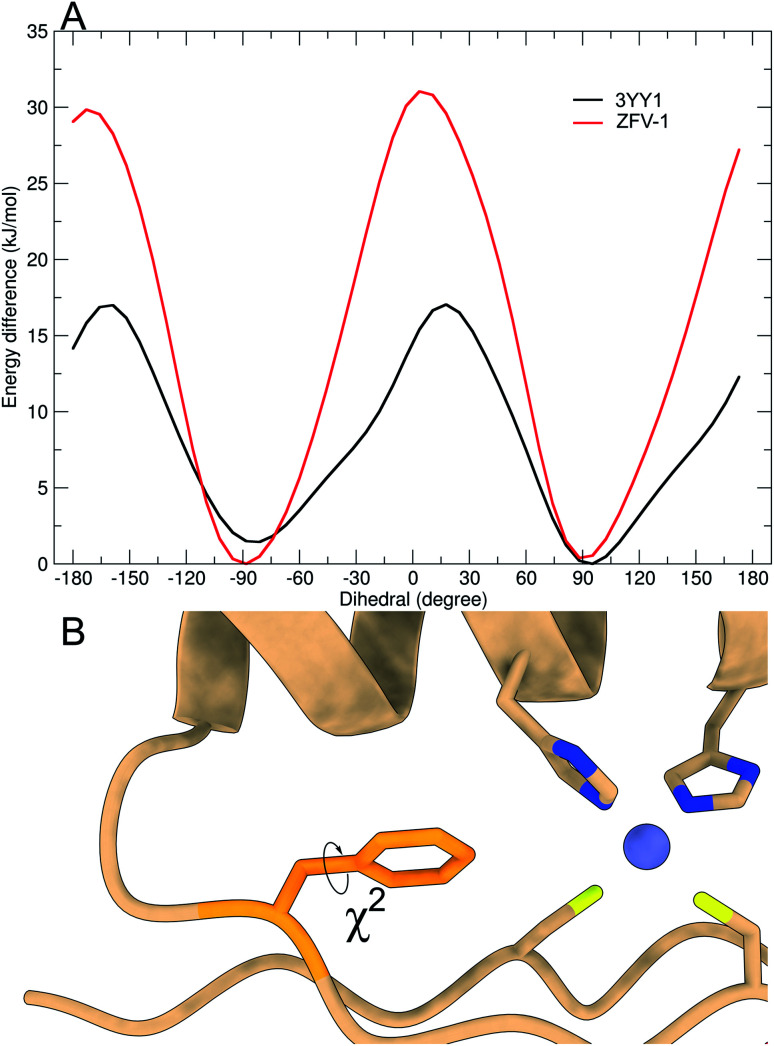
Free energy profile of Phe^13^ calculated during the metadynamics simulation, biasing the *χ*^2^-dihedral (A). Structural excerpt of ZFV-1 with focus on the central Phe^13^ and its *χ*^2^-dihedral (B).

As depicted in Fig. 3, out of all phenylalanine amino acids, Phe^6^ has the most flexible aromatic side chain. Even though it shows a face-to-face packing with His,^26^ it appears to perform numerous turns around its *χ*^2^-dihedral in the 1.5 μs MD simulation. Hot=Tap rigidifies the β-turn, thus restricting the rotational mobility of Phe^6^. This impact can also be observed in ^1^H NMR, as the H^26β*ProR*^-proton experiences a strong upfield shift of 0.5 ppm, due to a stronger aromatic ring anisotropy. Phe^17^ which is situated at the beginning of the α-helix rotates freely. Furthermore, no immediate long-range effect on Phe^13^ and Phe^2^ is visible as both *χ*^2^-dihedral mobilities remain similar in both zinc finger peptides. In contradiction to the TOCSY/NOESY-pattern, Phe^13^ is only able to perform a single 180° turn during the simulation for 3YY1 but is unable to rotate in ZFV-1. The high energy barrier makes this occurrence statistically unlikely to be sampled during a classical MD simulation in 1.5 μs. This difference in experimental results and simulated outcome is one major challenge of MD simulations since the timespan sampled by standard methods lies in the ns to μs range. In contrast, the timescale in ^1^H NMR spectroscopy is typically much longer. Trying to resolve this discrepancy, enhanced sampling techniques have been developed to further improve the timescale visited during a simulation. We chose well-tempered (wt) metadynamics to investigate the *χ*^2^ flexibility of Phe^13^ since it is an established method for the computational study of dynamic systems.^48–51^ The flexibility during the wt-metadynamics simulation increases dramatically and multiple turns can be observed for Phe^13^ in both peptides. Additionally, since only one local parameter was biased, no severe change in the flexibility of the other Phe could be observed and shown in Fig. 3 for Phe^2^, Phe^6^ and Phe^17^. One remarkable advantage of wt-metadynamics is the ability to readily produce the free energy surface alongside a biased parameter. We compared the free energy surface between 3YY1 and ZFV-1 and depicted them in Fig. 4. Even though the β-turn is, by no means, in the vicinity of the hydrophobic core, its influence is considerable. 3YY1 has an energy barrier of 17 kJ mol^−1^, while the incorporation of Hot=Tap results in an increase to 31 kJ mol^−1^. This large impact can be explained by the change in rigidity in the β-sheet and difference in the turn-type. The Glu–Gly motif favored a type-II turn, while the replacement with Hot=Tap forces the peptide to adopt a type-II′ turn. This feature has also been observed in the literature for the BTD derivative.^28,29^ Besides the rigidifying effect in proximity, Hot=Tap also exerts a long-range interaction by stiffening the β-sheet itself. This reduction in the flexibility of the secondary structure raises the energy barrier for the rotation of Phe^13^ around the *χ*^2^-dihedral.

After establishing the “baseline” for the flexibility of Phe^13^ inside the hydrophobic core and the influence of β-turn modification, we wanted to gain further insight into the dynamics of this central amino acid. Wüthrich *et al.* have established methods for the analysis of slow ring-flips, using elaborate temperature gradients.^3^ Since the thermodynamic stability of zinc finger miniproteins is limited to around 330 K (ESI†), this method is not applicable here. We therefore sought to replace the aromatic moiety with synthetic Phe-analogs, to further gain insight into its orientation. In the past few decades, studies have focused on replacing this conserved position with non-aromatic amino acids to assess its importance for structural integrity and thermodynamic stability.^16,18,20,23^ Since the library of natural aromatic amino acids is limited and has either been investigated (Tyr) or didn't fold (Trp), we focused on expanding this set of building blocks. As mentioned in the Introduction, one example of an unnatural amino acid was the implementation of cyclohexyl alanine in the zinc finger motif.^21^ While this side chain has a similar hydrophobicity, the complex spin system with its many spins impedes a direct identification of the side chain orientation. Therefore, we employed the well-established Negishi cross coupling strategy of aryl-iodides in combination with *N*-Boc-3-iodo-l-alanine methyl ester to quickly generate unnatural building blocks with spin systems analogous to phenylalanine, as depicted in the ESI.†^52,53^

**Fig. 5:**
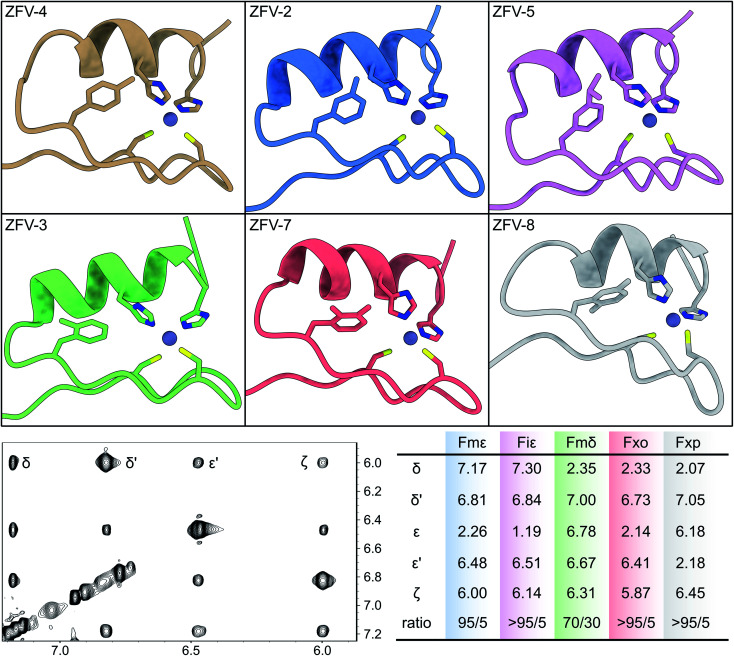
Top row: the lowest energy conformer of the NMR structure ensemble for six Phe^13^-substituted zinc fingers, determined by 2D-NMR. Bottom row left: aromatic region of the ZFV-2 TOCSY spectra with large chemical dispersion. On the bottom right: table of the chemical shift values (*X*^13^) for each modified zinc finger in combination with the orientation ratio, whereas the first number indicates facing towards solvent, outside the hydrophobic core.

We were able to obtain the modified Phe amino acids of Fig. 1 in moderate to good yields, which were deprotected by hydrolysis followed by Fmoc protection. These seven unnatural building blocks were introduced into the zinc finger sequence by standard Fmoc-based solid-phase peptide synthesis. To evaluate the orientation of the side chain and rotational dynamics, each aromatic position was systematically modified. Even though, Fmζ exhibits structural similarities to Tyr (which also has a modification on the ζ-position) we wanted to include this building block to evaluate the influence of methylation on each aromatic position. As expected, ZFV-4 was able to adapt a stable ββα fold though we observed that a higher pH value was needed to facilitate complete folding. NMR structure analysis showed a structure in close analogy to the tyrosine derivative, with the methyl group facing outwards of the hydrophobic core towards the solvent. This disposition leaves a small cleft inside the hydrophobic core, which is compensated for by Leu^19^ advancing inside the core. Furthermore, ZFV-4 exhibits the same dynamics as Phe and Tyr, namely an AA′BB′ spin system. For clarity, only the lowest NMR structure for each variant is depicted in Fig. 5, while an ensemble of 10 conformers is represented in the ESI.† Generally, all derivatives were able to adopt a zinc finger type fold, as typical chemical shift values remained the same and TOCSY/NOESY patterns differentiated only slightly.

Because of the steric requirements of the hydrophobic core, the introduction of a methyl group in either the delta or the epsilon position should favor one specific orientation. Furthermore, breaking the rotational symmetry of the aromatic ring was expected to yield more NOE contacts. This was most successful for ZFV-2, as a defined set of 5 distinct TOCSY/NOE signals (4 in the aromatic region plus one signal in the aliphatic region) could be observed (Fig. 5). Analysis revealed that the methyl group is orientated towards the solvent, outside of the hydrophobic core. Facing inside the hydrophobic core is disfavored since competition with Leu19 would distort the hydrophobic core, resulting in thermodynamic instability. Besides the main NOE signals, additional signals arise at low intensity. This signal set corresponds to the disfavored orientation inside the hydrophobic core, as indicated by few key NOE contacts (ESI†). By evaluating the relative intensity of both conformers which are in fast chemical exchange, we can estimate that the methyl group is orientated inside the core to only ∼5%, based on the NOE signal volume proportion.

In contrast to ZFV-4, methylation on the epsilon position allows for edge to face interaction with His^22^, as visible in the NMR structure (Fig. 5). Therefore, ZFV-2 shows a remarkable thermodynamic stability, exhibiting a good signal to noise ratio even at 320 K. To further rigidify the rotational flexibility of Phe^13^, the Fiε building block was introduced, which increases the steric bulk at the epsilon position significantly. This zinc finger derivative (ZFV-5) depicts a near identical fold in comparison to ZFV-2. Furthermore, no second signal set could be observed for the disfavored orientation inside the hydrophobic core. However, a decrease in thermal stability could be detected. After evaluating the zeta and epsilon positions, we introduced the Fmδ-building block. Despite the structural similarity to Fmε and Fmζ, ZFV-3 displays a different behavior. Though the peptide clearly retains the classical ββα fold, a decrease in structural stability, as the sample degrades after several days and loses its S/N ratio at higher temperature with no reversibility, could be observed. Additionally, aromatic signals, while clearly visible, are broadened in comparison to those of other derivatives at lower temperature, which hints at a deceleration of the rotation around *χ*^2^ on the edge of slow chemical exchange. 2D NMR analysis revealed that the conformer ratio has shifted to around 70/30. This behavior could also be observed in the NMR structure ensemble. In the 10 lowest energy conformers, several examples of the methyl group facing inside the zinc finger core could be observed. The results indicate that the hydrophobic core opposes certain geometrical restrictions. Both Fmδ and Fmε contain the same steric bulk, yet ZFV-3 shows more rotational flexibility, signifying a better fit for the methyl group inside the hydrophobic core than in ZFV-2. The increased mobility broadens the aromatic signals. Unfortunately, the limited range of thermal stability makes large temperature gradients inaccessible to gain a detailed description for this zinc finger variant. Following the same approach, the steric demand on the delta position with Fiδ was increased. After a straightforward synthesis, we first analyzed the apo peptide, to verify the successful incorporation of the building block. This was followed by the addition of zinc in aqueous solution and resulted in a zinc finger like fold. Surprisingly, no aromatic signals for Phe^13^ and for residues 14–18 which, in the zinc finger structure, are in proximity to Phe^13^, could be observed. Therefore, structural analysis of this zinc finger derivative was incomplete. One explanation for this behavior may be found in the rotation around *χ*^2^, which is considerably slowed down and thus leads to the broadening of the aromatic signals. Due to increased steric demand, a rotation around *χ*^2^ would require the secondary structural features to change conformation to allow passing of the isopropyl residue. If this conformational change is slow enough, broadening would make the signals from residues 14–18 undetectable. After the evaluation of single position modified phenylalanine derivatives, we investigated the influence of multiple modifications. This way, competition experiments could further aid in understanding of the rotational dynamics inside the hydrophobic core. As we have demonstrated, modification on the zeta position provides no benefit during our investigation of the rotational behavior. Therefore, only double methylation, which would break the symmetry, at the delta and epsilon positions was evaluated. The Fxo building block, which features syn-type methylation, was successfully introduced into the zinc finger moiety, and exhibits a very stable ββα fold (ZFV-7). Strong resemblance is present to ZFV-2 and only one rotamer, facing outside of the hydrophobic core, could be identified. In contrast to this, ZFV-8, which contains methyl groups at opposite positions, still adopt the same tertiary fold, but a displacement of the aromatic ring, similar to Tyr and ZFV-4, could be observed. Consequently, the most shielded aromatic signal is now the epsilon proton, because of edge-to-face stacking against the imidazole residue of His^22^. Furthermore, all aromatic signals for Fxp^13^ are broadened at 290 K hinting at underlying rotational mobility, yet no second signal set for the other orientation was observed.

**Fig. 6:**
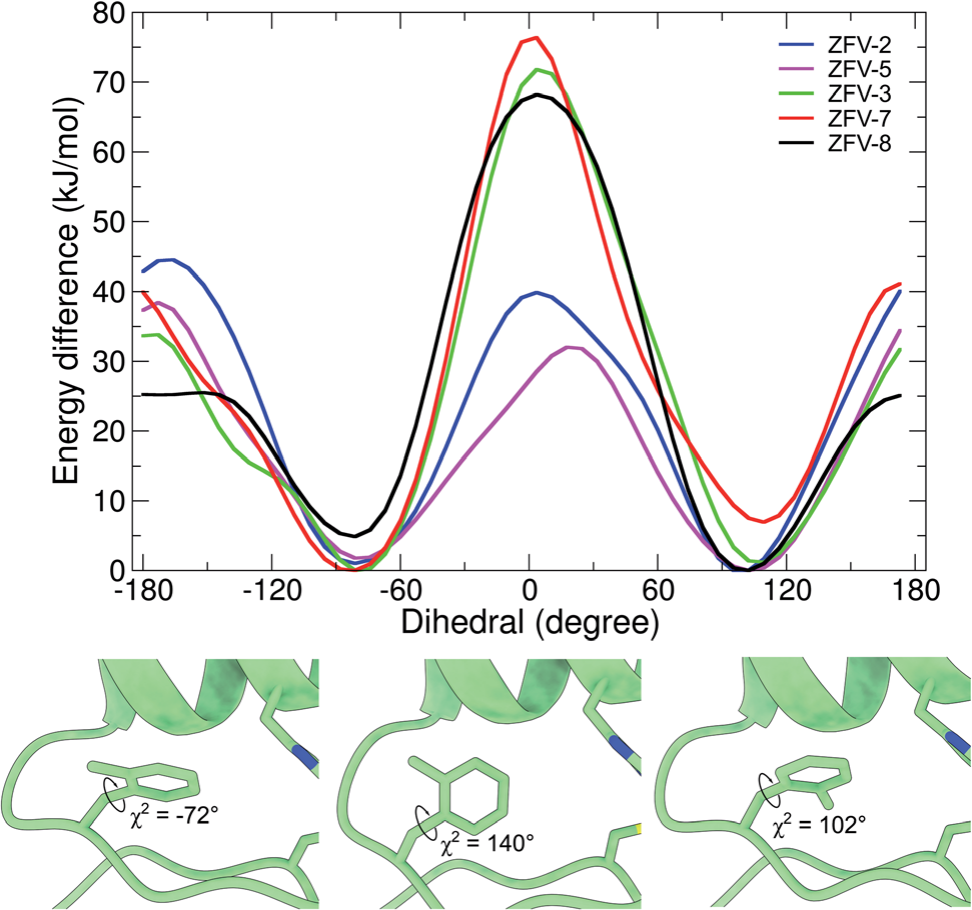
Free energy profile of the biased dihedral for synthetic zinc finger variants with a Phe^13^ substituted hydrophobic core. Snapshots obtained at different timeframes during the wt-metadynamics simulation of ZFV-3, which shows the stepwise rotation inside the hydrophobic core for Fmδ.^13^

Motivated by the experimental results for these synthetic zinc fingers, we performed MD (ESI†) and wt-metadynamics simulations of the stable zinc finger variants. The results are summarized in Fig. 6. As expected, all zinc finger peptides exhibit the same mobility for Phe^6^ and Phe^17^, since they are remote to the point of mutation and are not influenced by long range effects. Phe^2^ rotational mobility around *χ*^2^ is only slightly hindered in the substituted zinc finger peptides; one reason could be the slight displacement of Leu^19^, which changes the steric environment of Phe^2^. Wt-metadynamics revealed that after the introduction of a methyl group in the epsilon position, an increase in the energy barrier to 40 kJ mol^−1^ could be detected. In contrast to expectations, the behavior changes in ZFV-5, which shows a significantly lower barrier, similar to ZFV-1. Methylation on the δ-position demonstrates only a slight increase of the energy barrier to 34 kJ mol^−1^ but the direction of rotation is strongly influenced. Fmδ is only able to rotate towards the α-helix because a 70 kJ mol^−1^ energy barrier prevents its rotation towards the β-sheet. As already evident from NMR analysis, double syn-like methylation in ZFV-7 generates a robust fold that bears great resemblance to ZFV-2. This characteristic is also reproduced in the wt-metadynamics simulation, as the progression of Fxo mimics a combination of Fmε and Fmδ with an energy barrier of 40 kJ mol^−1^ but also preferred rotational orientation. Temperature-dependent broadening of aromatic signals for Fxp could be observed in the NMR spectra, indicating slow chemical exchange. The energy profile obtained during the simulation confirmed this observation, as the double methylation at opposite positions lowers the energy barrier to 25 kJ mol^−1^, where again a preferential direction of rotation towards the α-helix could be noted. The direct conversion of these energy barriers in ring flip rates using the Eyring equation^54^ overestimates rates, since wt-metadynamics seems to underestimate the energy barrier for the rotation in the zinc finger hydrophobic core. This systematic deviation is avoided by comparing the relative rotation rates of the side chains. Relative to the MD flip rate of 10^6^ for 3YY1 we estimate the factors of ZFV-1 1 × 10^4^, ZFV-2 1 × 10^2^, ZFV-3 1 × 10^3^, ZFV-5 5 × 10^2^, ZFV-7 1 × 10^2^ and ZFV-8 1 × 10^5^. Upon the introduction of Fmε,^13^ the ring flip rate is reduced by a magnitude of 10^4^. The modification of the delta position only reduces the flip rate by 10^3^. The scaled rates highlight the potential that these building blocks have for the characterization of side chain dynamics.

## Conclusion and outlook

After the first investigations on zinc finger structures in 1989,^15^ it was soon detected that the conserved aromatic residue exhibits flexible dynamics. In the following years, the studies have focused on the elucidation of these dynamics by NMR relaxation methods or complete swapping of the aromatic residue in the hydrophobic core.^20–22^ We extended this search by the systematic variation of the *X*^13^ position in 3YY1, using unnatural phenylalanine amino acids. These building blocks showed different methylation/isopropylation patterns, which resulted in the splitting of the AA′BB′C spin system and allowed detailed investigation of the rotational dynamics. The systematic variation of the position of the substituents identifies zinc finger variants with fast ring flips caused by decreased barriers from ground state elevation over selective line broadening in ^1^H NMR from the intermediate exchange of the aromatic ring at position Phe^13^. The approach presented here is applicable to temperature sensitive proteins like zinc fingers. For each of the nine modified zinc finger derivatives, NMR structure ensembles were generated, using two-dimensional NMR spectroscopy, and displayed local and global changes of the zinc finger structure depending on the modification pattern. To overcome the discrepancy between experimental and theoretical results, well-tempered metadynamics simulation, biasing the *χ*^2^-dihedral, was employed. The analysis of the dynamics demonstrated that the rotation of *X*^13^ was independent of other residues and a small energy barrier for 3YY1 and ZFV-1 could be observed, allowing for a fast rotation. The synthetic phenylalanine derivatives resulted in different rotational behavior. Epsilon modification locked the ring in one position, while delta substitutions allowed for a rotation inside the hydrophobic core. By expanding the steric bulk to isopropyl, successful incorporation induced mobility, which distorts the secondary features considerably and resulted in the broadening of the signals beyond detection. Simultaneous modification on delta and epsilon positions on the same site again resulted in locked orientation while opposite methylation allows the ring to rotate freely due to a low energy barrier. This behavior leads to selective broadening of Fxp^13^ signals in NMR spectra at room temperature and is amplified at lower temperatures. To describe the mobility of the synthetic phenylalanine derivatives in more detail, wt-metadynamics simulations were carried out, the results of which agree with the observations from NMR spectroscopy in many areas. While epsilon methylation slightly increases the rotational barrier, delta methylation generates a preferred rotational direction, as the energy barrier for the rotation towards the β-hairpin is highly restricted. Double methylated derivatives (Fxo and Fxp) exhibit an energy profile similar to that of Fmδ and Fmε. Of particular interest is Fxp, since wt-metadynamics show a decrease in the energy barrier, compared to ZFV-1. However, despite the low energy barrier and signal broadening, no additional set of NOE contacts for the second orientation could be detected in the strongly restricted temperature range of ZFV-8.

In summary, we developed methods to use modified aryl amino acids as sensors to probe side chain dynamics on the positions of phenylalanine in temperature sensitive miniproteins. Experimental and computational methods were performed using a sensitive zinc finger fold as a model peptide. By applying systematic methyl-hopping around the aromatic ring, the rotational symmetry of the *χ*^2^-dihedral is broken, thus generating new insightful contacts in NMR spectroscopy. Based on these results, we can conclude that the hydrophobic core of the 3YY1 zinc finger can accept single methylation at every position of the Phe^13^ residue. However, stability and rotational mobility strongly correlate with the modified position. The most stable structures were studied by wt-metadynamics simulations, which are in good agreement with the experimental outcomes. The quantification of the restricted mobility of a buried Phe is applicable to other synthetically accessible proteins containing Phe and Tyr, especially when expanded to a molecular library approach. Further research could focus on the symmetric substitution of phenylalanine residues, which would result in an equilateral increase in the rotational barrier. This in turn would facilitate the elucidation of the rotational behavior by temperature gradients for stable miniproteins. We hope that the methodology of combining systematic methyl hopping and wt-metadynamics becomes a useful tool for the study of aromatic side chain dynamics in the hydrophobic core of miniproteins.

## Author contributions

AG contributed the design and financial support of the research project. PH performed synthesis, NMR, and conducted the modeling studies. Both authors participated in the data interpretation, wrote and edited the manuscript.

## Conflicts of interest

We declare no conflict of interest.

## Supplementary Material

SC-012-D1SC02346B-s001
