# Supplementary material for: High five! Methyl probes at five ring positions of phenylalanine explore the hydrophobic core dynamics of zinc finger miniproteins
Source: Chem Sci. 2021 Jul 24;12(34):11455–63. doi: 10.1039/d1sc02346b (PMC8447250; doi:10.1039/d1sc02346b)
Supplement: SC-012-D1SC02346B-s001 [file SC-012-D1SC02346B-s001.pdf]

Electronic Supplementary Material (ESI) for Chemical Science.
This journal is © The Royal Society of Chemistry 2021

Supporting Information

High five! Methyl probes at five ring positions of phenylalanine explore the hydrophobic core dynamics of zinc finger miniproteins.

Philip Horx, Armin Geyer

Table of Contents

1) Experimental Procedures 2

Chemicals and Materials 2

Instrumentation 2

Computational Details 3

2) Synthetic Procedures 5

3) Supplementary Results and Discussions 11

Purity and pre folding comparison of 3YY1 vs ZFV-1 11

Important NOE-signals to define the X13 orientation 13

4) Analytical Data for zinc finger peptides 16

5) Variable temperature NMR 38

6) Supplementary Computational Results 43

7) Supplementary spectroscopic Data for unnatural building blocks 55

8) References 69

Experimental Procedures

Chemicals and Materials. Chemicals were purchased from different vendors and used without further purification. SPPS building blocks and materials were purchased from Iris Biotech. Reaction solvents were distilled and then degassed via freeze-pump-thaw or extrusion with argon. DMF (peptide grade) was bought from Iris Biotech and stored under inert atmosphere. Reactions were monitored using TLC on Merck silica gel TLC 60 F_{254} plates. Visualization of spots was facilitated using UV light and $KMnO_4$ stain or ninhydrin stain, respectively. Crude products were purified by column chromatography using silica gel by Macherey-Nagel 60 M (0.040-0.063 mm with 230-400 mesh). The modified phenylalanine analogues were synthesized by employing a Negishi-type cross-coupling of the corresponding aryl halides with Boc-Iodo-Ala-OMe. All peptides were synthesized on an automated peptide Synthesizer (*Liberty Blue*, *CEM,* 0.1-0.05 mmol scale). 2-Chlorotritylchloride resin (1.60 mmol/g) was loaded with Fmoc-Gly-OH by adding 1.50 eq of the protected amino acid and 6.00 eq DIPEA in DMF 10mL/g resin. This suspension was shaken for 4 h and washed with DMF, methanol and DCM several times and treated with a mixture of DCM/methanol/DIPEA (80:15:5) two times for 30min. After washing several times with DMF, methanol and DCM the resin was dried, 1 mg was weighed and treated with 20% piperidine in DMF for 30 min. Afterwards the loading was determined to be between 0.40 and 0.45 mmol/g by UV-VIS spectroscopy, using the wavelengths of 298 nm and 300 nm. After successful synthesis and final deprotection the resin was washed several times with DMF and DCM. The resin was dried under high vacuum for 3 h and treated with a mixture of TFA/H_2O/phenol/TIPS/EDT (84:4:4:4:4) 8 mL for 5 h. The filtrate was concentrated either by evaporation under high vacuum or by blowing nitrogen over it, added to cold diethyl ether (35 mL) which led to precipitation of the desired peptide. Following this, the precipitate was washed several times with DEE and finally lyophilized from water/acetonitrile. Peptides were purified on a Thermo Fisher Ultimate 3000 LC System by semipreparative reversed-phase high-performance liquid chromatography (HPLC). An Macherey-Nagel VP Nucleodur C18 Gravity with an inside diamterer of 150 mm x 21 mm was used. Gradients varied from 10 to 40 or 15 to 40% B in 60 min (A, water and 0.1% TFA; B, acetonitrile and 0.085% TFA) with a flow rate of 15 mL/min. An ACE UltraCore 2.5 SuperC18 (150 x 2.1 mm) with a flow rate of 0.45 mL/min was used for all the analytical runs. The chromatograms of the pure fractions are depicted in the Supporting Info.

NMR-Instrumentation. All one- and two-dimensional NMR spectra were acquired on Bruker AV II 300, Bruker HD-500, Bruker AV III 500 or AV II 600 spectrometers. All chemical shifts are reported in ppm using the signal of solvents DMSO-d6: 1H: 2.50 ppm, 13C: 39.52 ppm or 3-(trimethylsilyl)-2,2,3,3,-tetradeuteropropionic acid sodium salt 0.00 ppm as reference. NMR spectra of peptides were measured using Watergate pulse sequence for water suppression. Signal assignment of all peptides was performed using nmrfam-sparky, utilizing TOCSY, NOESY. Multiplicity are described as d = dublet, t= triplet, q = quartet, quint = quintet, dd = double dublet. Peptidic NMR samples were prepared in the following way: First 1.5 mg of the lyophylized peptide was dissolve in 400 µL H_2O, followed by additon of 40µL ZnCl2-solution (1.5 eq.) and 60 µL D2O. The pH-value was then adjusted to 5.8-7.2 with 2 µL aliquots. The required pH-value for folding was determined by pH-titration and measuring 1D-NMR in

selected steps. After reaching the desired pH-value additional H2O was added to reach the final sample volume (580 µl). Each solvent was degassed prior to use by using a syringe and bubbling argon through the solution for 30 min. For stable derivatives, the sample could be used up to 2 weeks, while unstable derivatives showed signal loss, or degradation of the whole spectra after several days. Mass spectra of all compounds were acquired on a Thermo Fisher Scientific LTQ-FT Ultra mass spectrometer using ESI+. Peptides were first purified with HPLC and then subjected to HPLC-MS.

Computational details. Structure determination of zinc finger peptides were performed using the Xplor-NIH suite of programs[1] NOE-restraints were extracted from 2D-NOESY spectra with a mixing time of 300 ms. For the native derivative mixing times of 100, 200 and 300 ms were evaluated. Integration was performed using NMRFAM-Sparky.[2] The cross-peaks were divided according to their intensities as weak, medium, or strong. This resulted in 115-205 restraints depending on the amount of folded structure. The calculations started from an extended conformation using the torsion angle dynamics simulated annealing protocol written by Stein et al.[3] Additionally, both termini were capped with n-terminal acetyl and c-terminal amide groups to avoid artificial charge-charge interactions. The system was heated to 3500 K and cooled down in 12.5 K steps. The eefx2 implicit force field was chosen since it yields in higher quality solution structures.[4] After the initial structure generation, a the refine protocol, implemented in Xplor-NIH, was applied to further improve structural quality. The structure with the lowest energy was chosen as the starting structure for all further molecular dynamics simulations. All structural representation were generated using ChimeraX.[5] Parameters for non-standard amino acid residues for NMR structure calculations were obtained using the charmm-gui ligand reader & modeler.[6,7] For peptides ZFV-6 the 2D-NMR spectra were missing key TOCSY and NOE-signals for some residues due to line broadening. All simulations were performed using the GROMACS 2018.4 suite.[8-11] For the native zinc finger, the respective fragment from the crystal structure of DNA-bound YY1 (pdb code: 1UBD) was taken. For all other derivatives, the lowest energy structure originating from the NMR calculations was chosen for input preparation and processed using the pdb2gmx program. The peptides were solvated with ~3000 TIP4P water molecules in a dodecahedron box. A salt concentration of 0.15M was added, in addition to charge-balancing counter ions, to neutralize the system and mimic physiological conditions. The relevance of the salt concentration for the stabilization of folded conformations has been outlined in several publications.[12-14] We evaluated different force field families for the 3YY1 zinc finger to assess the robustness of our results (Amber99SBILDN, CHARMM36, AMBER14SB and AMBER15) by comparing RMSD, RMSF, radius of gyration for the whole peptide and χ^2 flexibility of the 4 phenylalanine residues. We observed that the secondary structure in amber14 and amber15 unfolds after 200-400 ns. Both, amber99sbildn and Charmm36 were able to stabilize both helix and hairpin structure for at least 1000ns. We chose the former since it has been employed numerous times for similar systems in the literature[15-17] and carried out all following calculation using this force field. After performing metadynamics we observed unfolding for other derivatives. Therefore, we employed a harmonic restraint on the zinc coordination site which stabilizes the fold for all simulations. Long-range electrostatic interactions were calculated using the Particle-mesh Ewald (PME) summation scheme.[18] Van der Waals and Coulomb interactions were truncated at 1.2 nm. All bonds involving a hydrogen atom were constrained using the LINCS algorithm.[19] For non-standard amino acid residues parameterization

has been performed using the acpype-server.[20] Derivative FIE and FXM were unable to produce stable zinc finger type fold after a certain number of nanoseconds. Therefore, we employed dihedral restraint to further stabilize the hairpin motif. Gromacs 2018.4 patched with plumed 2.4 was used for all wt-metadynamics simulations to evaluate the FES of the X^{13}-residues.[21] The χ2-dihedral of X^{13} was biased (Cα-Cβ-Cγ-Cδ1). A Gaussian with a height of 0.125 kJ/mol and bias factor of 3 was deposited every 2 ps. Attempts of increasing the height and bias factor led to a more rugged energy surface and an imbalance between the two energy basins in 3YY1 was observed. Further increasing the height led to instances of unfolding. To estimate the conversion of the free energy surface during the simulation, a surface analysis was performed every 250ns. The corresponding energy surfaces are depicted in the supporting information. For every metadynamics the equilibrated structure of the standard md-simulation after 100ns was taken as the input-structure. Derivatives ZFV-3 and ZFV-7 required the increase of the biasfactor to overcome the large energy barrier. We observed low structural stability for ZFV-3 and ZFV-8 during the wt-metadynamics, even after the introduction of additional dihedral restraint in the backbone. Therefore, we inserted harmonic restraints for the hydrogen bonds within the β-hairpin. Thus, a stable tertiary structure was obtained. We estimate that the influence on the resulting energy profile to be small, since the derivative ZFV-7, which did not receive such restraints, also exhibits a similarly high energy barrier at 0°.

Synthetic Procedures

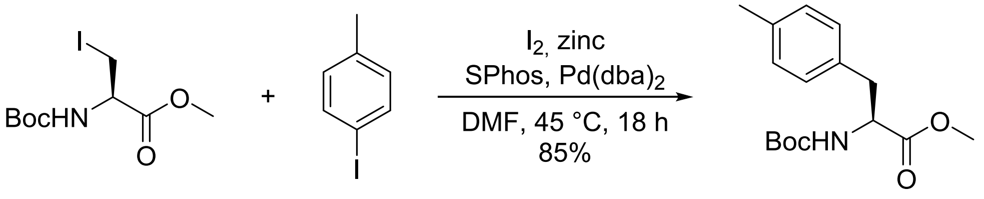

S1 [329.13] **S2** [218.04] **S3** [293.36]

A pressure flask was charged with previously activated zinc (297 mg, 4.56 mmol, 3.00 eq), iodine (57 mg, 0.23 mmol, 0.15 eq) and 3.00 mL DMF. The flask was charged with argon and after 5 min additional iodine (57 mg, 0.23 mmol, 0.15 eq) was added, followed by N-Boc-3-iodo-L-alanine methyl ester (*Org. Synth.* **2015**, *92*, 103-116.), (**S1**, 500 mg, 1.52 mmol, 1.00 eq) which resulted in an exothermic reaction. After complete insertion of zinc $Pd(dba)_2$ (87 mg, 0.15 mmol, 0.10 eq), Sphos (62 mg, 0.15 mmol, 0.10 eq) und 1-Iodo-4-methylbenzene (**S2**, 430 mg, 1.97 mmol, 1.30 eq) were added. The flask was sealed and stirred for 18 h at 45 °C. After completion, the mixture was diluted with ethyl acetate and filtered through a pad of celite. The filtrate was concentrated under vacuo, resuspended in ethyl acetate and washed with water and brine. The organic phase was dried over magnesium sulfate, separated and the solvent removed under vacuo. Purification was achieved by column chromatography on silica using toluene/ethyl acetate to provide 380 mg (1.29 mmol, 85%) of phenylalanine derivate **S3** as a brown solid.

1H-NMR: 500 MHz, $DMSO_{d6}$, δ = 7.23 (d, ^3J = 8.1 Hz, 1H, NH), 7.10 (d, ^3J = 8.0 Hz, 2H, CH_{arom}), 7.07 (d, ^3J = 8.0 Hz, 2H, CH_{arom}), 4.12 (ddd, ^3J = 9.7, 8.1, 5.3 Hz, 1H, Hα), 3.59 (s, 3H, OMe), 2.93 (dd, ^2J = 13.6 Hz, ^3J = 5.0 Hz, 1H, Hβ), 2.80 (dd, ^2J = 13.7 Hz, ^3J = 9.7 Hz, 1H, Hβ), 2.25 (s, 3H, C4-CH_3), 1.32 (s, 9H, Boc). 13C-NMR: 75 MHz, $DMSO_{d6}$, δ = 172.6 (*C*OOMe), 155.2 (Boc-CO), 135.4 (C4), 134.4 (C1), 128.9 (C3), 128.7 (C2), 78.2 (*C*$(CH_3)_3$), 55.3 (Cα), 51.7 (OMe), 36.0 (Cβ), 28.1 (C$(CH_3)_3$), 20.6 (C4-CH_3) ppm. HRMS (ESI+): Calcd for $C_{16}H_{23}NO_4Na$ $[M+Na]^+$: 316.1519, found: 316.1521. TLC: R_f = 0.24 (toluene:ethyl acetat 19:1).

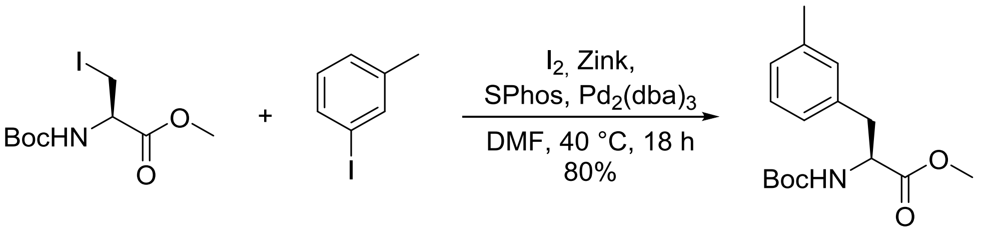

S1 [329.13] **S4** [218.04] **S5** [293.36]

A pressure flask was charged with previously activated zinc (418 mg, 6.39 mmol, 3.00 eq), iodine (81 mg, 0.32 mmol, 0.15 eq) and 3.00 mL DMF. The flask was charged with argon and after 5 min additional iodine (81 mg, 0.32 mmol, 0.15 eq) was added, followed by N-Boc-3-iodo-L-alanine methyl ester (*Org. Synth.* **2015**, *92*, 103-116.), (**S1**, 700 mg, 2.13 mmol, 1.00 eq) which resulted in an exothermic reaction. After complete insertion of zinc $Pd_2(dba)_3$ (107 mg, 0.11 mmol, 0.05 eq), Sphos (87 mg, 0.21 mmol, 0.10 eq) und 1-Iodo-3-methylbenzene (**S4**, 603 mg, 2.77 mmol, 1.30 eq) were added. The flask was sealed and stirred for 18 h at 40 °C. After completion, the mixture was diluted with ethyl acetate and filtered through a pad of celite. The filtrate was concentrated under vacuo, resuspended in ethyl

acetate, washed with water and brine. The organic phase was dried over magnesium sulfate, separated and the solvent removed under vacuo. Purification was achieved by column chromatography on silica using toluene/ethyl acetate to provide 499 mg (1.70 mmol, 80%) of phenylalanine derivate **S5** as a brown solid.

1H-NMR: 300 MHz, $DMSO_{d6}$, δ = 7.27 (d, ^3J = 8.2 Hz, 1H, NH), 7.16 (t, ^3J = 7.5 Hz, 1H, Hε‘), 7.05-6.99 (m, 3H, Hδ, Hδ‘, Hζ), 4.15 (ddd, ^3J = 9.7, 8.2, 5.0 Hz, 1H, Hα), 3.60 (s, 3H, OMe), 2.94 (dd, ^2J = 13.8 Hz, ^3J = 5.1 Hz, 1H, Hβ), 2.80 (dd, ^2J = 13.7 Hz, ^3J = 10.0 Hz, 1H, Hβ), 2.27 (s, 3H, ε-CH_3), 1.33 (s, 9H, Boc) ppm. 13C-NMR: 75 MHz, $DMSO_{d6}$, δ = 172.5 (*C*OOMe), 155.3 (Boc-CO), 137.4 (Cγ), 137.1 (Cε), 129.6 (Cδ), 128.0 (Cε‘), 127.0 (Cζ), 126.1 (Cδ‘), 78.2 (*C*(CH_3)$_3$), 55.1 (Cα), 51.7 (OMe), 36.4 (Cβ), 28.1 (C(*C*H_3)$_3$), 20.9 (Cε-*C*H_3) ppm. HRMS (ESI+): Calcd. for [$C_{16}H_{24}NO_4$] $[M+H]^+$: 295.1733, found: 295.1727. TLC: R_f = 0.55 (toluene:ethyl acetate 9:1).

According to this procedure, only the temperature was varied, and the following derivatives have been prepared.

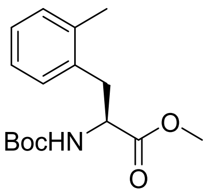

S6 [293.36]

40 °C, 514 mg, 1.75 mmol, 82%, brown solid. 1H-NMR: 500 MHz, $DMSO_{d6}$, δ = 7.32 (d, ^3J = 8.3 Hz, 1H, NH), 7.16-7.07 (m, 4H, Hδ‘, Hε, Hε‘, Hζ), 4.17 (dt, ^3J = 9.7, 5.3 Hz, 1H, Hα), 3.60 (s, 3H, OMe), 3.02 (dd, ^2J = 14.0 Hz, ^3J = 5.2 Hz, 1H, Hβ), 2.84 (dd, ^2J = 14.0 Hz, ^3J = 10.0 Hz, 1H, Hβ), 2.27 (s, 3H, δ-CH_3), 1.32 (s, 9H, Boc) ppm. 13C-NMR: 75 MHz, $DMSO_{d6}$, δ = 172.6 (*C*OOMe), 155.3 (Boc-CO), 136.0 (Cδ), 135.7 (Cγ), 130.0 (Cε), 129.6 (Cδ‘), 126.5 (Cζ), 125.6 (Cε‘), 78.2 (*C*(CH_3)$_3$), 53.7 (Cα), 51.7 (OMe), 34.0 (Cβ), 28.1 (C(*C*H_3)$_3$), 18.8 (Cδ-*C*H_3) ppm. HRMS (ESI+): Calcd. for [$C_{16}H_{23}NO_4Na$] $[M+Na]^+$: 316.1519, found: 316.1530. TLC: R_f = 0.20 (toluene:ethyl acetat 19:1).

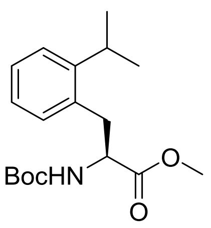

S7 [321.42]

45 °C, 835 mg, 2.60 mmol, 57%, brown oil. 1H-NMR: 300 MHz, $DMSO_{d6}$, δ = 7.34 (d, ^3J = 8.3 Hz, 1H, NH), 7.27 (d, ^3J = 7.3 Hz, 1H, Hε), 7.19 (dt, ^3J = 6.7 Hz, ^4J = 1.3 Hz, 1H, Hζ), 7.15-7.00 (m, 2H, Hδ‘, Hε‘), 4.12 (dt, ^3J = 8.7, 4.6 Hz, 1H, Hα), 3.59 (s, 3H, OMe), 3.11 (sept, ^3J = 6.6 Hz, 1H, Cδ-C*H*-CH_3), 3.08 (dd, ^2J = 14.2 Hz, ^3J = 5.3 Hz, 1H, Hβ), 2.87 (dd, ^2J = 14.2 Hz, ^3J = 9.8 Hz, 1H, Hβ), 1.31 (s, 9H, Boc), 1.18 (dd, ^3J = 6.7 Hz, ^4J = 2.0 Hz, 6H, δ-CH-C*H*$_3$) ppm. 13C-NMR: 75 MHz, $DMSO_{d6}$, δ = 172.5 (*C*OOMe), 155.3 (Boc-CO), 146.7 (Cδ), 134.0 (Cγ), 130.1 (Cδ‘), 126.9 (Cζ), 125.2 (Cε‘), 125.2 (Cε), 78.2 (*C*(CH_3)$_3$), 54.8 (Cα), 51.7 (OMe), 33.5 (Cβ), 28.1 (C(*C*H_3)$_3$), 27.9 (Cδ-*C*H-CH_3) 24.0 (Cδ-CH-*C*H_3), 23.7 (Cδ-CH-*C*H_3) ppm. HRMS (ESI+): Calcd. for [$C_{18}H_{27}NO_4Na$] $[M+Na]^+$: 344.1832, found: 344.1841. TLC: R_f = 0.22 (DCM:MeOH 100:0.7).

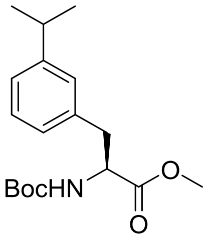

S8 [321.42]

50 °C, 826 mg, 2.57 mmol, 85%, brown oil. 1H-NMR: 600 MHz, $DMSO_{d6}$, δ = 7.26 (d, ^3J = 8.1 Hz, 1H, NH), 7.19 (t, ^3J = 7.8 Hz, 1H, Hε‘), 7.09-7.06 (m, 2H, Hδ, Hζ), 7.03 (d, ^3J = 7.5 Hz, 1H, Hδ‘), 4.17 (dt, ^3J = 9.0, 5.2 Hz, 1H, Hα), 3.60 (s, 3H, OMe), 2.96 (dd, ^2J = 13.8 Hz, ^3J = 5.0 Hz, 1H, Hβ), 2.87-2.80 (m, 2H, Hβ, ε-C*H*-CH_3), 1.32 (s, 9H, Boc), 1.18 (d, ^3J = 6.9 Hz, 6H, ε-CH-C*H*$_3$) ppm. 13C-NMR: 150 MHz, $DMSO_{d6}$, δ = 172.6 (*C*OOMe), 155.3 (Boc-CO), 148.2 (Cε), 137.4 (Cγ), 128.1 (Cε‘), 127.0 (Cδ), 126.5 (Cδ‘), 124.3 (Cζ), 78.2 (*C*(CH_3)$_3$), 55.0 (Cα), 51.7 (OMe), 36.5 (Cβ), 33.3 (Cε-*C*H-CH_3),

28.1 (C(CH_3)$_3$), 23.9 (Cε-CH-CH_3), 23.9 (Cε-CH-CH_3) ppm. HRMS (ESI+): Calcd. for [$C_{18}H_{27}NO_4Na$] [M+Na]$^+$: 344.1832, found: 344.1841. TLC: R_f = 0.40 (DCM:MeOH 100:0.8).

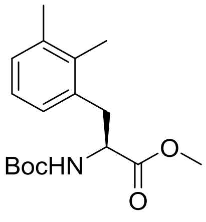

S9 [307.39]

45 °C, 750 mg, 2.44 mmol, 67%, brown oil. 1H-NMR: 300 MHz, DMSO$_{d6}$, δ = 7.31 (d, ^3J = 8.2 Hz, 1H, NH), 7.03-6.96 (m, 3H, CHarom) 4.09 (dd, ^3J = 8.3 Hz, 5.0 Hz, 1H, Hα), 3.60 (s, 3H, OMe), 3.04 (dd, ^2J = 14.0 Hz, ^3J = 5.0 Hz, 1H, Hβ), 2.84 (dd, ^2J = 13.6 Hz, ^3J = 9.5 Hz, 1H, Hβ), 2.22 (s, 3H, ε-CH_3), 2.17 (s, 3H, δ-CH_3), 1.32 (s, 9H, Boc) ppm. 13C-NMR: 75 MHz, DMSO$_{d6}$, δ = 172.7 (*C*OOMe), 155.4 (*C*OO*t*Bu), 136.3 (Cγ), 135.5 (Cε), 134.5 (Cδ), 128.2 (Cζ), 127.9 (Cε‘), 124.9 (Cδ‘), 78.2 (*C*-(CH_3)$_3$), 54.2 (Cα), 51.7 (OMe), 34.6 (Cβ), 28.1 (C-(*C*H_3)$_3$), 20.3 (ε-CH_3), 14.6 (δ-CH_3) ppm. HRMS (ESI+): Calcd. for [$C_{17}H_{25}NO_4Na$] [M+Na]$^+$: 331.1709, found: 331.1712. TLC: R_f = 0.42 ($CHCl_3$).

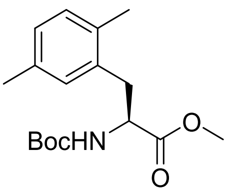

S10 [307.39]

45 °C 895 mg, 2.91 mmol, 80%, beige solid. 1H-NMR: 300 MHz, DMSO$_{d6}$, δ = 7.31 (d, ^3J = 8.1 Hz, 1H, NH), 7.02 (d, ^3J = 7.5 Hz, 1H, Hε), 6.94 (s, 1H, Hδ‘), 6.92 (d, ^3J = 7.5 Hz, 1H, Hζ), 4.15 (dd, ^3J = 8.4 Hz, 5.2 Hz, 1H, Hα), 3.60 (s, 3H, OMe), 2.97 (dd, ^2J = 14.2 Hz, ^3J = 5.2 Hz, 1H, Hβ), 2.80 (dd, ^2J = 13.6 Hz, ^3J = 9.5 Hz, 1H, Hβ), 2.22 (s, 6H, δ-CH_3, ε‘-CH_3), 1.32 (s, 9H, Boc) ppm. 13C-NMR: 75 MHz, DMSO$_{d6}$, δ = 172.9 (*C*OOMe), 155.6 (*C*OO*t*Bu), 135.7 (Cγ), 134.5 (Cε‘), 133.0 (Cδ), 130.4 (Cε), 130.1 (Cδ‘), 127.3 (Cζ), 78.4 (*C*-(CH_3)$_3$), 53.9 (Cα), 51.0 (OMe), 34.2 (Cβ), 28.3 (C-(*C*H_3)$_3$), 20.7 (Cε‘-CH_3), 18.6 (Cδ-CH_3) ppm. HRMS (ESI+): Calcd. for [$C_{17}H_{25}NO_4Na$] [M+Na]$^+$: 330.1676, found: 330.1676. TLC: R_f = 0.31 ($CHCl_3$).

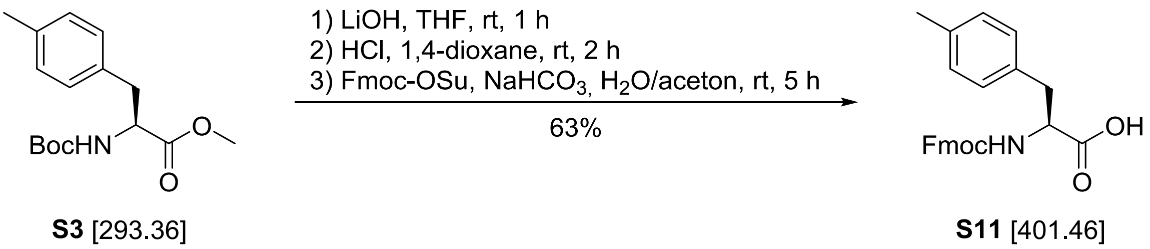

S3 [293.36] **S11** [401.46]

A round bottom flask was charged with derivate **S3** (370 mg, 1.27 mmol. 1.00 eq), 6.5 mL THF and lithium hydroxide (1M in water, 6.5 mL, 6.50 mmol, 5.12 eq). After completion of the reaction the pH was adjusted to pH=2 and extracted with ethyl acetate (3x). The organic phase was dried over magnesium sulfate, filtrated and the solvent removed in vacuo. Afterwards hydrogen chloride in 1,4-dioxane (4M, 2.0 mL, 8.00 mmol, 6.30 eq) was added and the solution stirred for 2 h. Removal of the solvent yielded the unprotected amino acid as a beige solid. The solid was dissolved in 5 mL water and charged with NaHCO3 (213 mg, 2.54 mmol, 2.00 eq). After this a solution of Fmoc-OSu (428 mg, 1.27 mmol, 1.00 eq) in 5 mL acetone was added slowly. The protection was completed after 5 h and the mixture acidified to pH=2. Extraction was conducted with ethyl acetate (3x), followed by drying of the organic phases over magnesium sulfate. The solvent was removed in vacuo and column chromatography using DCM:MeOH:AcOH furnished Fmoc-ζ-methyl-l-phenylalanine (322 mg, 0.80 mmol, 63% over 3 steps) as a colorless solid.

1H-NMR: 500 MHz, DMSO$_{d6}$, δ = 7.88 (d, ^3J = 7.6 Hz, 2H, Fmoc-CH$_{arom}$), 7.67 (d, ^3J = 8.6 Hz, 1H, NH), 7.64 (t, ^3J = 7.4 Hz, 2H, Fmoc-CH$_{arom}$), 7.41 (dt, ^3J = 7.5, 2.5 Hz, 2H, Fmoc-CH$_{arom}$),

7.31 (dt, ^3J = 7.5 Hz, ^4J = 1.0 Hz, 1H, Fmoc-CH_{arom}), 7.28 (dt, ^3J = 7.5 Hz, ^2J = 1.0 Hz, 1H, Fmoc-CH_{arom}), 7.15 (d, ^3J = 8.0 Hz, 2H, H2), 7.07 (d, ^3J = 8.0 Hz, 2H, H3), 4.22-4.11 (m, 4H, Fmoc-CH_2, Fmoc-CH, Hα), 3.03 (dd, ^2J = 14.0 Hz, ^3J = 4.6 Hz, 1H, Hβ), 2.83 (dd, ^2J = 14.0 Hz, ^3J = 10.3 Hz, 1H, Hβ), 2.25 (s, 3H, ζ-CH_3) ppm. 13C-NMR: 75 MHz, $DMSO_{d6}$, δ = 173.3 (COOH), 155.9 (Fmoc-CO), 143.7, 140.7 (Fmoc-C_{arom}), 135.3 (C4), 134.8 (C1), 128.9 (C2), 128.7 (C3), 127.6, 127.0, 125.2, 121.0 (Fmoc-CH_{arom}), 65.6 (Fmoc-CH_2), 55.6 (Cα), 46.6 (Fmoc-CH), 36.1 (Cβ), 20.6 (CH3) ppm. HRMS (ESI+): Calcd. for [$C_{25}H_{23}NO_4Na$] $[M+Na]^+$: 424.1519, found: 424.1524. TLC: R_f = 0.40 (DCM:MeOH:AcOH 20:1:0.05).

According to this method the following derivatives have been prepared:

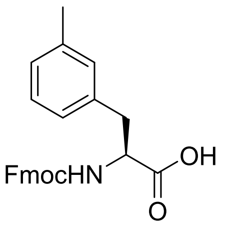

S12 [401.46]

570 mg, 1.42 mmol, 86%, colorless solid. 1H-NMR: 300 MHz, $DMSO_{d6}$, δ = 12.72 (s, 1H, COOH), 7.88 (d, ^3J = 7.5 Hz, 2H, Fmoc-CH_{arom}), 7.71 (d, ^3J = 8.5 Hz, 1H, NH), 7.66 (t, ^3J = 6.9 Hz, 2H, Fmoc-CH_{arom}), 7.41 (t, ^3J = 7.6 Hz, 2H, Fmoc-CH_{arom}), 7.30 (dq, ^3J = 7.7 Hz, ^4J = 1.0 Hz, 2H, Fmoc-CH_{arom}), 7.16 (t, ^3J = 7.7 Hz, 1H, Hε‘), 7.07 (s, 1H, Hδ), 7.03 (d, ^3J = 6.2 Hz, 1H, Hδ‘), 7.01 (d, ^3J = 7.6 Hz, 1H, Hζ), 4.23-4.10 (m, 4H, Fmoc-CH_2, Fmoc-CH, Hα), 3.05 (dd, ^2J = 13.8 Hz, ^3J = 4.2 Hz, 1H, Hβ), 2.83 (dd, ^2J = 13.8 Hz, ^3J = 10.5 Hz, 1H, Hβ), 2.25 (s, 3H, ε-CH_3) ppm. 13C-NMR: 75 MHz, $DMSO_{d6}$, δ = 173.5 (COOH), 155.8 (Fmoc-CO), 143.7, 140.7, 140.6 (Fmoc-C_{arom}), 138.0 (Cγ), 137.1 (Cε), 129.7 (Cδ), 128.0 (Cε‘), 127.6, 127.0 (Fmoc-CH_{arom}), 126.9 (Cζ), 126.1 (Cδ‘) 125.3, 125.2, 120.0 (Fmoc-CH_{arom}), 65.6 (Fmoc-CH_2), 55.7 (Cα), 46.6 (Fmoc-CH), 36.6 (Cβ), 21.0 (CH3) ppm. HRMS (ESI+): Calcd. for [$C_{25}H_{23}NO_4Na$] $[M+Na]^+$: 424.1519, found: 424.1520. TLC: R_f = 0.55 (DCM:MeOH:AcOH 25:1:0.05).

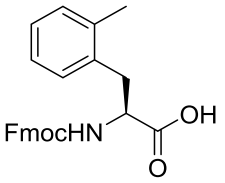

S13 [401.46]

780 mg, 1.94 mmol, 83%, off-white solid. 1H-NMR: 600 MHz, $DMSO_{d6}$, δ = 12.78 (s, 1H, COOH), 7.88 (d, ^3J = 7.7 Hz, 2H, Fmoc-CH_{arom}), 7.78 (d, ^3J = 8.7 Hz, 1H, NH), 7.66 (d, ^3J = 7.6 Hz, 1H, Fmoc-CH_{arom}), 7.64 (d, ^3J = 7.6 Hz, 1H, Fmoc-CH_{arom}), 7.41 (dt, ^3J = 7.4 Hz, ^4J = 4.2 Hz, 2H, Fmoc-CH_{arom}), 7.32 (dt, ^3J = 7.4 Hz, ^4J = 0.8 Hz, 1H, Fmoc-CH_{arom}), 7.29 (dt, ^3J = 7.4 Hz, ^4J = 0.8 Hz, 1H, Fmoc-CH_{arom}), 7.21 (dd, ^3J = 6.6 Hz, ^4J = 1.9 Hz, 1H, Hδ‘), 7.14 (dd, ^3J = 7.0 Hz, ^4J = 1.8 Hz, 1H, Hε), 7.10 (dt, ^3J = 7.3 Hz, ^4J = 1.7 Hz 1H, Hζ), 7.08 (dt, ^3J = 7.4 Hz, ^4J = 1.4 Hz, 1H, Hε‘), 4.22-4.10 (m, 4H, Fmoc-CH_2, Fmoc-CH, Hα), 3.12 (dd, ^2J = 14.2 Hz, ^3J = 4.4 Hz, 1H, Hβ), 2.86 (dd, ^2J = 14.1 Hz, ^3J = 10.4 Hz, 1H, Hβ), 2.30 (s, 3H, δ-CH_3) ppm. 13C-NMR: 75 MHz, $DMSO_{d6}$, δ = 173.4 (COOH), 155.9 (Fmoc-CO), 143.7, 140.6 (Fmoc-C_{arom}), 136.1 (Cγ), 136.0 (Cδ), 130.0 (Cε), 129.7 (Cδ‘), 127.6, 127.0 (Fmoc-CH_{arom}), 126.4 (Cε‘), 125.5 (Cζ), 125.2, 125.2, 120.1 (Fmoc-CH_{arom}), 65.6 (Fmoc-CH_2), 54.1 (Cα), 46.5 (Fmoc-CH), 34.0 (Cβ), 18.8 (Cδ-CH_3) ppm. HRMS (ESI+): Calcd. for [$C_{25}H_{23}NO_4Na$] $[M+Na]^+$: 424.1519, found: 424.1543. TLC: R_f = 0.28 (Cyc:EtOAc:AcOH 10:7:0.05).

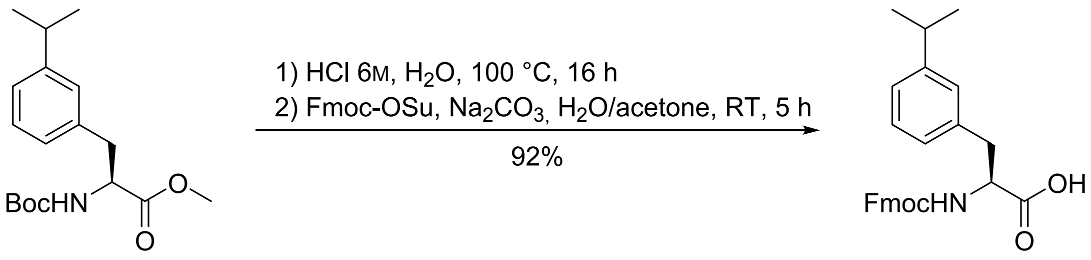

S7 [321.42] **S14** [429.52]

A round bottom flask was charged with ε-*iso*-propyl-L-Phenylalanine methyl ester **S7** (604 mg, 1.88 mmol, 1.00 eq) and 5. 00mL hydrogen chloride (6M, 30 mmol, 16.0 eq). The suspension was heated to 100 °C and stirred for 16 h. Removal of the solvent in vacuo yielded a residue that was titrated with diethyl ether and dissolved in 20 mL water. The solution was cooled to 0 °C and Fmoc-OSu (621 mg, 1.84 mmol, 0.98 eq) in 20 mL acetone was added slowly, followed by adjustment of the pH-value to 9 with 10% sodium carbonate solution. This was followed by removal of the cooling bath and stirring of the solution for 5 h. Completion of the reaction was monitored by TLC. Acidification to pH=2 was accomplished by addition of 2M hydrogen chloride, followed by precipitation of the crude product. The precipitate was filtered and washed with water (3x) dried in vacuo. Column chromatography furnished Fmoc-ε-*iso*-propyl-L-Phenylalanine **S14** (743 mg, 1.73 mmol, 92%) as a colorless solid.

1H-NMR: 500 MHz, $DMSO_{d6}$, δ = 7.87 (d, ^3J = 7.6 Hz, 2H, Fmoc-CH_{arom}), 7.71 (d, ^3J = 8.4 Hz, 1H, NH), 7.65 (t, ^3J = 7.5 Hz, 2H, Fmoc-CH_{arom}), 7.40 (ddt, ^3J = 7.5 Hz, ^4J = 3.0, 0.8 Hz 2H, Fmoc-CH_{arom}), 7.30 (dt, ^3J = 7.5 Hz, ^4J = 1.0 Hz, 1H, Fmoc-CH_{arom}), 7.27 (dt, ^3J = 7.5 Hz, ^4J = 1.0 Hz, 1H, Fmoc-CH_{arom}), 7.18 (t, ^3J = 7.7 Hz, 1H, Hε'), 7.15 (s, 1H, Hδ), 7.08-7.05 (m, 2H, Hδ', Hζ), 4.23-4.14 (m, 4H, Fmoc-CH_2, Fmoc-CH, Hα), 3.06 (dd, ^2J = 13.9 Hz, ^3J = 4.3 Hz, 1H, Hβ), 2.86 (dd, ^2J = 13.8 Hz, ^3J = 10.6 Hz, 1H, Hβ), 2.83 (sept, ^3J = 6.9 Hz, 1H, ε-C*H*-CH_3) 1.16 (dd, ^3J = 6.9 Hz, ^4J = 4.7 Hz, 6H, ε-CH-$(CH_3)_2$) ppm. 13C-NMR: 75 MHz, $DMSO_{d6}$, δ = 173.3 (COOH), 155.9 (Fmoc-CO), 148.1 (Cε), 143.7, 143.7, 140.6 (Fmoc-C_{arom}), 137.8 (Cγ), 128.1 (Cε'), 127.6 (Fmoc-CH_{arom}), 127.1 (Cδ), 127.0 (Fmoc-CH_{arom}), 126.4 (Cδ'), 125.2, 125.2 (Fmoc-CH_{arom}), 124.2 (Cζ), 120.0 (Fmoc-CH_{arom}), 65.6 (Fmoc-CH_2), 55.4 (Cα), 46.6 (Fmoc-CH), 36.5 (Cβ), 33.3 (*C*$(CH_3)_2$), 23.8 (C$(CH_3)_2$) ppm. HRMS (ESI+): Calcd. for [$C_{27}H_{27}NO_4Na$] $[M+Na]^+$: 452.1832, found: 452.1844. TLC: R_f = 0.36 (DCM:MeOH:AcOH 25:1:0.05).

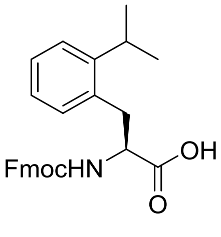

S15 [429.52]

862 mg, 2.08 mmol, 79%, colorless solid. 1H-NMR: 500 MHz, $DMSO_{d6}$, δ = 12.80 (s, 1H, COOH), 7.88 (d, ^3J = 7.6 Hz, 2H, Fmoc-CH_{arom}), 7.80 (d, ^3J = 8.6 Hz, 1H, NH), 7.67 (d, ^3J = 7.4 Hz, 1H, Fmoc-CH_{arom}), 7.64 (d, ^3J = 7.4 Hz, 1H, Fmoc-CH_{arom}), 7.40 (ddt, ^3J = 7.5 Hz, ^4J = 4.6, 0.8 Hz 2H, Fmoc-CH_{arom}), 7.32 (dt, ^3J = 7.5 Hz, ^4J = 1.0 Hz, 1H, Fmoc-CH_{arom}), 7.30-7.26 (m, 2H, Fmoc-CH_{arom}, Hε), 7.22-7.16 (m, 2H, Hδ', Hζ), 7.06 (dt, ^3J = 7.5 Hz, ^4J = 1.3 Hz, 1H, Hε'), 4.23-4.14 (m, 3H, Fmoc-CH_2, Fmoc-CH), 4.11 (dt, ^3J = 9.3, 4.3 Hz, 1H, Hα), 3.25-3.17 (m, 2H, δ-C*H*-$(CH_3)_2$, Hβ), 2.85 (dd, ^2J = 14.0 Hz, ^3J = 10.6 Hz, 1H, Hβ), 1.19 (d, ^3J = 7.0 Hz, 3H, δ-CH-(C*H*$_3$)$_2$), 1.17 (d, ^3J = 7.0 Hz, 3H, δ-CH-(C*H*$_3$)$_2$) ppm. 13C-NMR: 75 MHz, $DMSO_{d6}$, δ = 173.4 (COOH), 155.9 (Fmoc-CO), 146.7 (Cδ), 143.7, 143.7, 140.6 (Fmoc-C_{arom}), 134.4 (Cγ), 130.3 (Cδ'), 127.6, 127.0 (Fmoc-CH_{arom}), 126.9 (Cζ), 125.2 (Cε), 125.2 (Cε'), 125.2, 120.0 (Fmoc-CH_{arom}), 65.6 (Fmoc-CH_2), 55.2 (Cα), 46.5 (Fmoc-CH), 33.6 (Cβ), 27.8 (*C*-$(CH_3)_2$), 24.1 (C(*C*H$_3$)$_2$), 23.7 (C(*C*H$_3$)$_2$) ppm. HRMS (ESI+): Calcd. for [$C_{27}H_{27}NO_4Na$] $[M+Na]^+$: 452.1832, found: 452.1843 ppm. TLC: R_f = 0.37 (DCM:MeOH:AcOH 25:1:0.05).

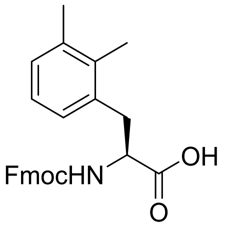

S16 [415.49]

650 mg, 1.56 mmol, 74%, colorless solid. 1H-NMR: 600 MHz, $DMSO_{d6}$, δ = 12.76 (s, 1H, COOH), 7.88 (d, ^3J = 7.6 Hz, 2H, Fmoc-CH_{arom}), 7.76 (d, ^3J = 8.6 Hz, 1H, NH), 7.67 (d, ^3J = 7.5 Hz, 1H, Fmoc-CH_{arom}), 7.64 (d, ^3J = 7.5 Hz, 1H, Fmoc-CH_{arom}), 7.41 (dt, ^3J = 7.2, 4.8 Hz 2H, Fmoc-CH_{arom}), 7.32 (dt, ^3J = 7.5 Hz, ^4J = 0.7 Hz, 1H, Fmoc-CH_{arom}), 7.30-7.26 (dt, ^3J = 7.5 Hz, ^4J = 0.7 Hz, 1H, Fmoc-CH_{arom}), 7.07 (d, ^3J = 7.3 Hz, 1H, Hζ), 7.00 (d, ^3J = 7.0 Hz, 1H, Hδ'), 6.97 (t, ^3J = 7.5 Hz, 1H, Hε'), 4.23-4.15 (m, 3H, Fmoc-CH_2, Fmoc-CH), 4.12 (dt, ^3J = 8.7, 4.4 Hz, 1H, Hα), 3.16 (dd, ^2J = 14.0 Hz, ^3J = 4.4 Hz, 1H, Hβ), 2.86 (dd, ^2J = 14.0 Hz, ^3J = 10.4 Hz, 1H, Hβ), 2.22 (s, 3H, ε-CH_3), 2.19 (s, 3H, δ-CH_3) ppm. 13C-NMR: 75 MHz, $DMSO_{d6}$, δ = 173.5 (COOH), 155.9 (Fmoc-CO), 143.7, 140.6 (Fmoc-C_{arom}), 136.3 (Cγ), 135.9 (Cε), 134.5 (Cδ), 128.1 (Cζ), 127.9 (Cε'), 127.6, 127.0 (Fmoc-CH_{arom}), 125.2 (Cδ'), 125.2, 120.0 (Fmoc-CH_{arom}), 65.6 (Fmoc-CH_2), 54.5 (Cα), 46.5 (Fmoc-CH), 34.7 (Cβ), 20.3 (Cε-CH_3), 14.6 (Cδ-CH_3) ppm. HRMS (ESI+): Calcd. for [$C_{26}H_{25}NO_4Na$] $[M+Na]^+$: 438.1676, found: 438.1680 ppm. TLC: R_f = 0.36 (DCM:MeOH:AcOH 25:1:0.05).

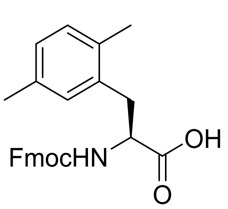

S17 [415.49]

854 mg, 2.06 mmol, 76%, colorless solid. 1H-NMR: 600 MHz, $DMSO_{d6}$, δ = 12.77 (s, 1H, COOH), 7.88 (d, ^3J = 7.6 Hz, 2H, Fmoc-CH_{arom}), 7.77 (d, ^3J = 8.6 Hz, 1H, NH), 7.67 (d, ^3J = 7.5 Hz, 1H, Fmoc-CH_{arom}), 7.64 (d, ^3J = 7.5 Hz, 1H, Fmoc-CH_{arom}), 7.41 (dt, ^3J = 7.3 Hz, ^3J = 4.9 Hz 2H, Fmoc-CH_{arom}), 7.31 (dt, ^3J = 7.5 Hz, ^4J = 0.7 Hz, 1H, Fmoc-CH_{arom}), 7.276 (dt, ^3J = 7.5 Hz, ^4J = 0.7 Hz, 1H, Fmoc-CH_{arom}), 7.04 (s, 1H, Hδ'), 7.01 (d, ^3J = 7.7 Hz, 1H, Hε), 6.91 (d, ^3J = 7.7 Hz, 1H, Hζ), 4.21-4.14 (m, 4H, Fmoc-CH_2, Fmoc-CH, Hα), 3.08 (dd, ^2J = 14.0 Hz, ^3J = 4.6 Hz, 1H, Hβ), 2.82 (dd, ^2J = 14.0 Hz, ^3J = 10.3 Hz, 1H, Hβ), 2.25 (s, 3H, ε'-CH_3), 2.18 (s, 3H, δ-CH_3) ppm. 13C-NMR: 75 MHz, $DMSO_{d6}$, δ = 173.5 (COOH), 155.9 (Fmoc-CO), 143.7, 140.7, 140.6 (Fmoc-C_{arom}), 135.9 (Cγ), 134.2 (Cε'), 132.8 (Cδ), 130.4 (Cε), 129.9 (Cδ'), 127.6, 127.0 (Fmoc-CH_{arom}), 127.0 (Cζ), 125.2, 125.2, 120.1 (Fmoc-CH_{arom}), 65.6 (Fmoc-CH_2), 54.2 (Cα), 46.6 (Fmoc-CH), 34.1 (Cβ), 20.6 (Cε'-CH_3), 18.4 (Cδ-CH_3) ppm. HRMS (ESI+): Calcd. for [$C_{26}H_{25}NO_4Na$] $[M+Na]^+$: 439.1710, found: 439.1709. TLC: R_f = 0.35 (DCM:MeOH:AcOH 25:1:0.05).

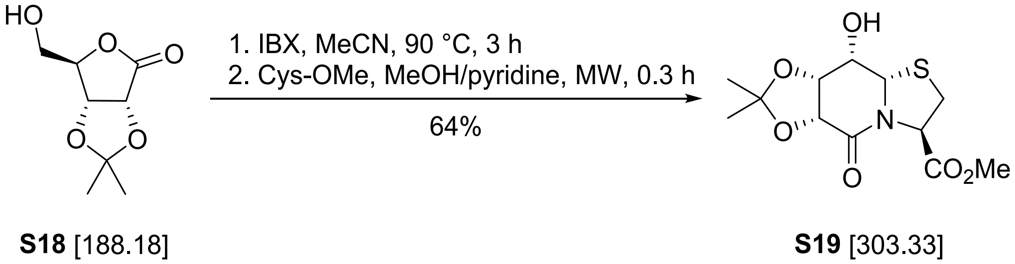

S18 [188.18] **S19** [303.33]

The procedure was adapted from Geyer et al. PNAS October 26, 2010 107 (43) 18336-18341, with microwave irradiation the reaction time can be drastically reduced. A flask was charged with alcohol **S18** (8.00 g, 42.5 mmol, 1.00 eq), 70.0 mL acetonitrile and IBX (15.5 g, 55.3 mmol, 1.30 eq) and heated to 90 °C for 3 h. The solution was cooled down to 0 °C for 1 h, filtrated and the supernatant washed with 30 mL acetonitrile. The solvent was removed in vacuo and the residue dissolved in pyridine (4.00 mL) and methanol (30.0 mL), followed by degassing with argon extrusion. After addition of cysteine methyl ester hydrochloride (8.76 g, 51.0 mmol, 1.20 eq) the solution was degassed again. The reaction was performed in a microwave (20 min, 75W, 90 °C) and after completion the solvent was removed in vacuo. The residue was dissolved in 20.0 mL dichloromethane and 20.0 mL hydrogen chloride (1m), the

phases were separated, and the aqueous phase was extracted with dichloromethane (3x). The combined organic phases were washed with saturated sodium hydrogen carbonate solution and dried over magnesium sulfate, filtrated and finally the solvent removed in vacuo. The bicyclic structure **S19** (8.25 g, 27.2 mmol, 64%) could be obtained as a yellow solid. The following steps to the final Fmoc-protected building block were taken from the literature.[22]

Results and Discussion

Purity and pre folding comparison of 3YY1 vs ZFV-1

As mentioned in the main article, introduction of Hot=Tap as a β-turn mimic, replacing Glu7 and Gly8 results in improved crude purity, higher purity after RP-HPLC, and higher solubility. To illustrate this, we displayed the RP-HPLC chromatograms below. Furthermore, the increased solubility could originate from a pre-folding of the β-hairpin due to the β-turn introducing effect from Hot=Tap. The 1H-NMR spectra show a nicely ordered structure which can be almost completely assigned using 2D-NMR techniques for ZFV-1 but not for 3YY1.

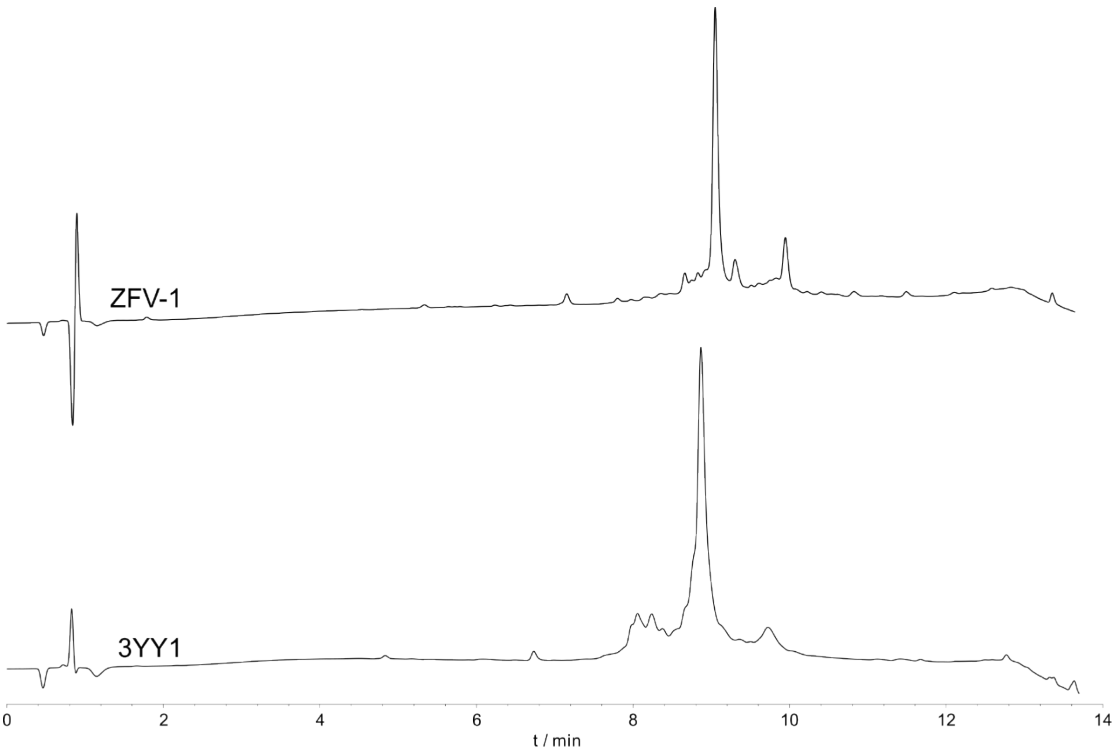

Figure S1: Comparison of the crude RP-HPLC chromatogram shows a slightly improved purity of ZFV-1 in comparison to 3YY1, since separation of the side products were facilitated.

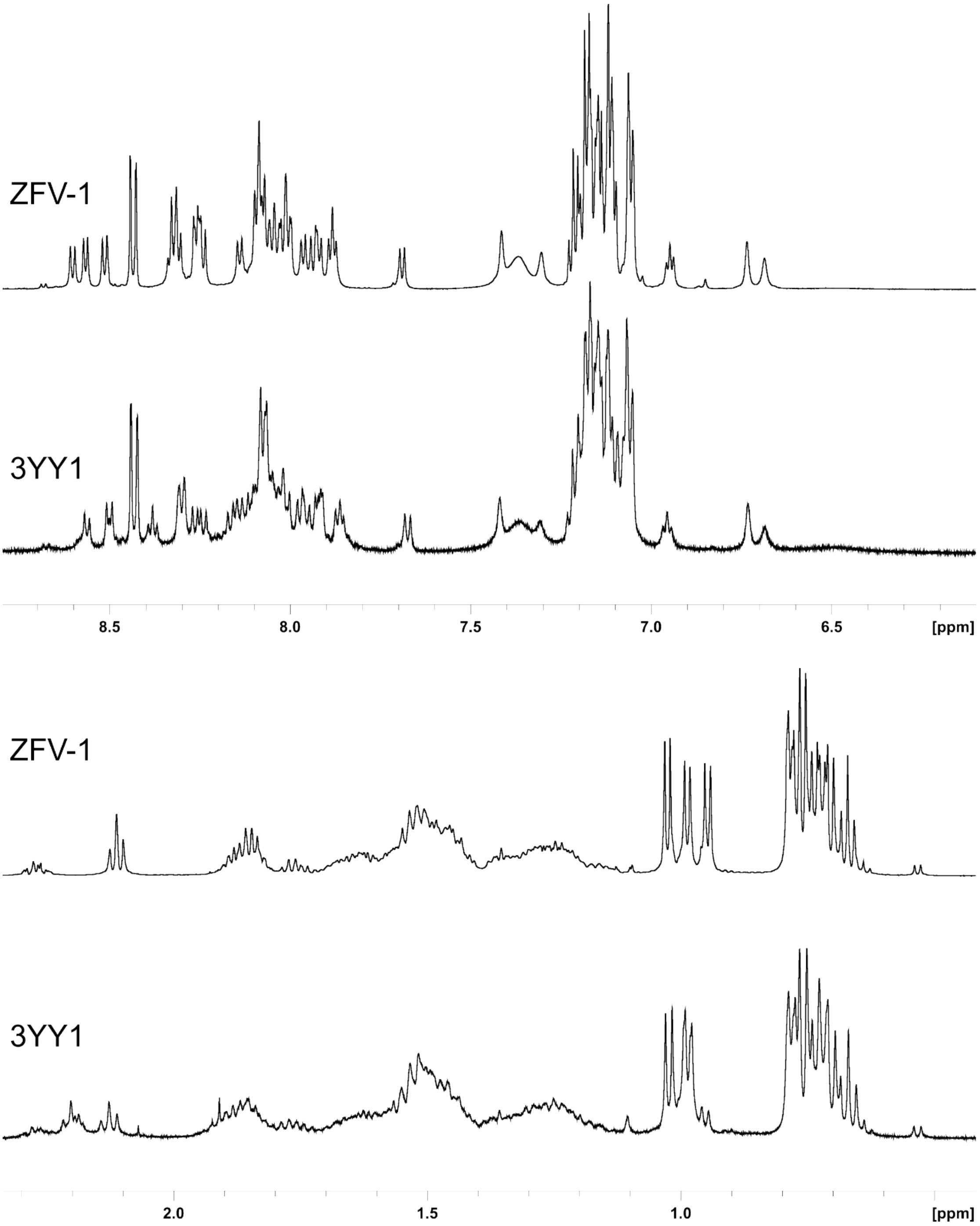

Figure S2: Comparison of pre folding between 3YY1 and ZFV-1. The upper spectra highlights the amidic region of both apo-peptides (600 MHz, 300 K, H_2O/D_2O 9:1, pH = 3.0). At the same concentration ZFV-1 not only exhibits a higher signal to noise ratio but also a higher dispersion. The lower spectra depict the aliphatic region where the differences in pre folding can be observed in the dispersion of the threonine methyl groups (1.0 ppm).

Important NOE-signals to define the X13 orientation

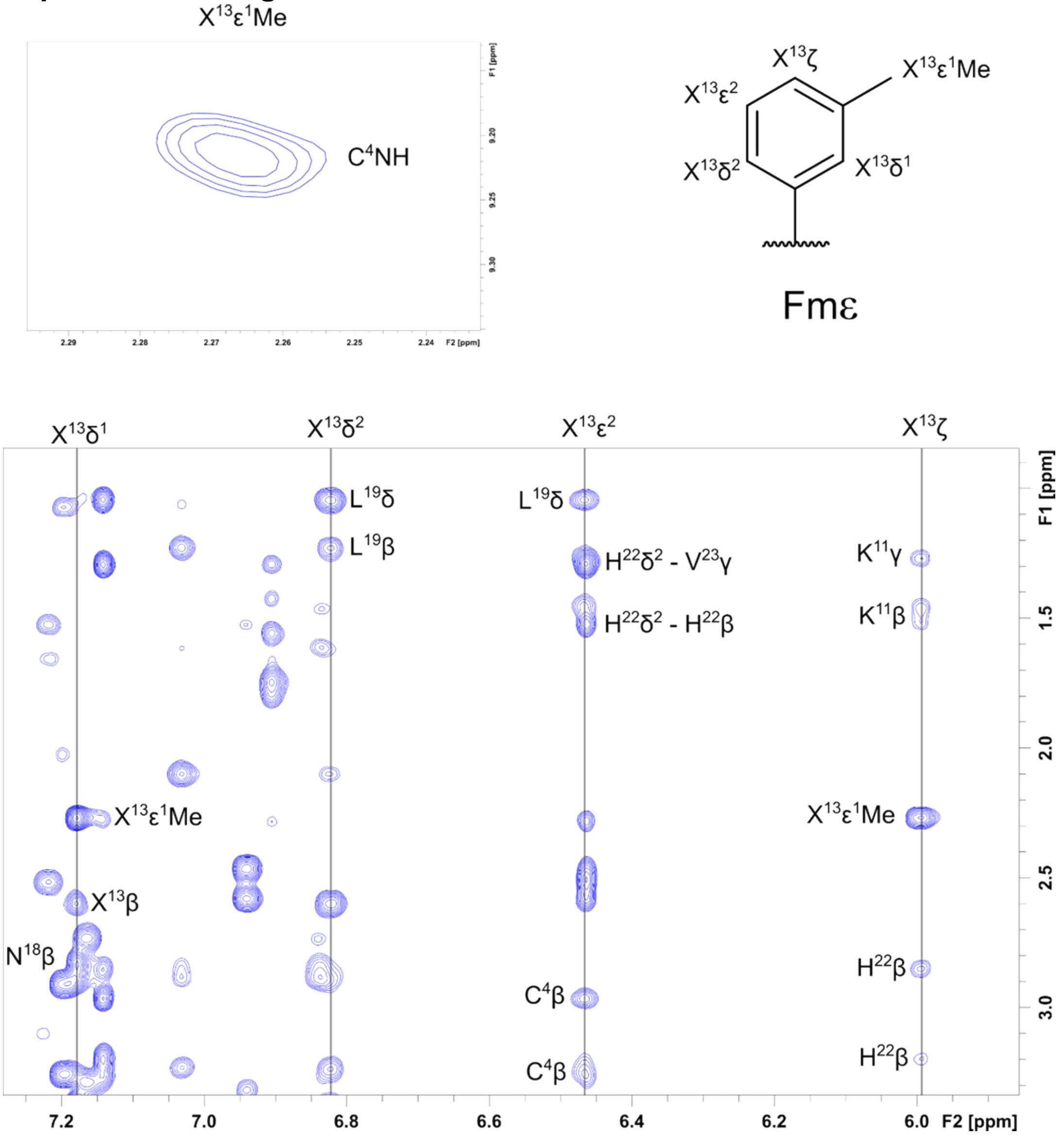

Figure S3: Key NOE-Signals for ZFV-2. The aromatic signals define the orientation of $Fm\varepsilon^{13}$, with the methyl group facing outwards oft he hydrophobic core. Only the contact of $Fm\varepsilon^{13}\varepsilon^{1}Me$ to $C^{4}NH$ indicates the other rotamer.

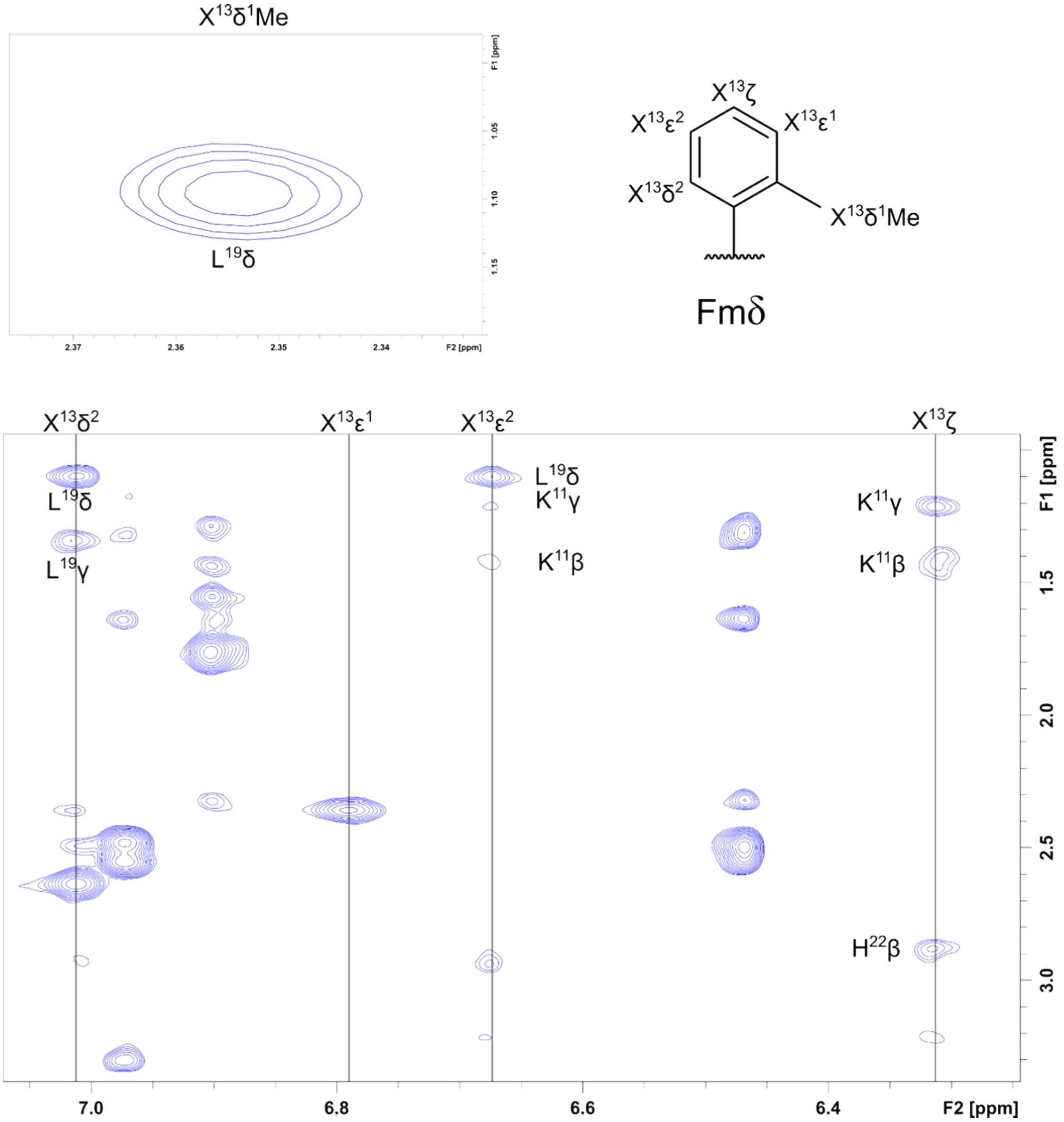

Figure S4: Key NOE-Signals for ZFV-3. The aromatic signals define the orientation of Fmδ13, with the methyl group facing outward oft he hydrophobic core. Only the contact of Fmδ13δ1Me to L19δ indicates the other rotamer.

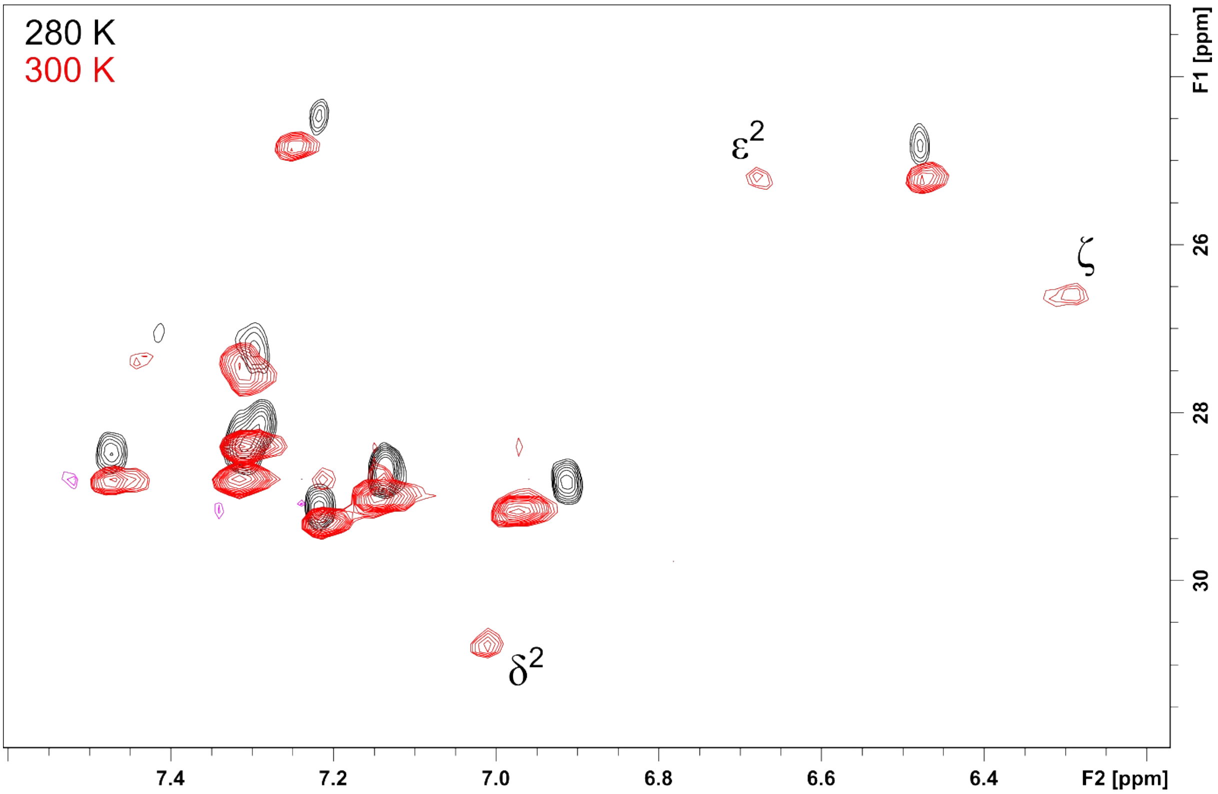

Figure S 5: Aromatic region of the 1H-13C-HSQC of ZFV-3 at different temperatures. The aromatic side chain signals of Fmδ13 are highlighted.

Analytical Data for zinc finger peptides

3YY1
$P^{1}F^{2}Q^{3}C^{4}T^{5}F^{6}E^{7}G^{8}C^{9}G^{10}K^{11}R^{12}F^{13}S^{14}L^{15}D^{16}F^{17}N^{18}L^{19}K^{20}T^{21}H^{22}V^{23}K^{24}I^{25}H^{26}T^{27}G^{28}$

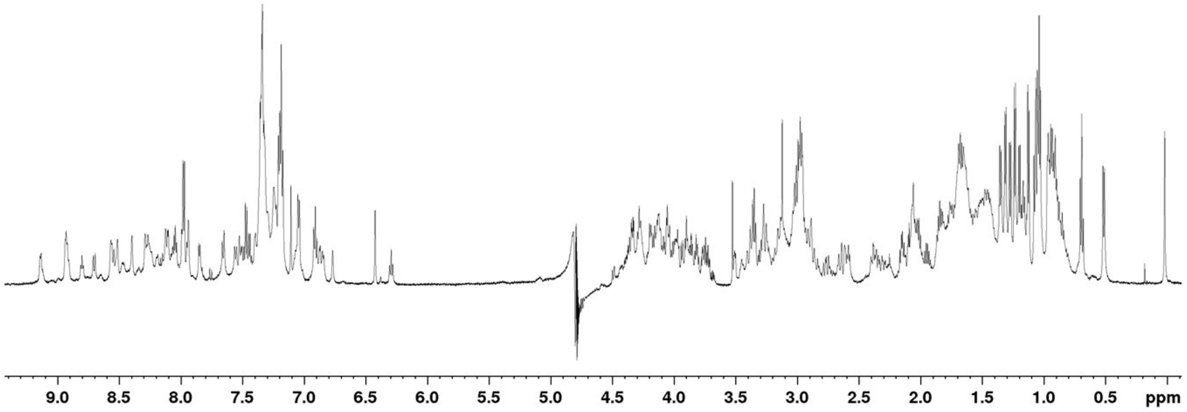

^{1}H-NMR: 600 MHz, 300K, H_2O/D_2O 9:1, 1.5 eq Zn^{2+}, pH = 6.0, n.d = not determinable, - = not applicable.

Amino acid	δ(NH) / ppm	δ(α) / ppm	δ(β) / ppm	δ(γ) / ppm	δ other / ppm
Pro^{1}	n.b.	4.26	2.35, 1.81	1.98	δ: 3.32
Phe^{2}	n.b.	4.80	3.24, 2.89	-	δ: 7.19
					ε, ζ: 7.45
Gln^{3}	8.91	5.07	2.09, 1.81	2.26, 2.10	ε: n.b.
Cys^{4}	9.12	4.55	3.33, 2.95	-	-
Thr^{5}	8.53	4.46	4.48	1.25	-
Phe^{6}	8.92	4.09	2.56, 2.36	-	δ: 7.03
					ε, ζ: 7.32
Glu^{7}	8.38	3.87	1.82, 1.73	2.13	-
Gly^{8}	8.79	4.15, 3.73	-	-	-
Cys^{9}	7.97	4.62	3.24, 2.95	-	-
Gly^{10}	8.26	4.04, 4.00	-	-	-
Lys^{11}	8.09	3.96	1.50-1.41	1.32-1.26	δ: 1.45-1.37
					ε: 3.02
Arg^{12}	7.92	4.99	1.62, 1.54	1.50, 1.42	δ: 3.09
					ε: 7.19
Phe^{13}	8.68	4.82	3.37, 2.62	-	δ: 7.17
					ε: 6.89
					ζ: 6.28
Ser^{14}	7.17	4.62	3.10, 3.07	-	-
Leu^{15}	7.54	4.86	1.59, 1.45	1.45	δ: 1.03
Asp^{16}	8.55	3.44	2.07, 2.03	-	-
Phe^{17}	8.49	4.11	3.28, 2.75	-	δ: 7.16
					ε, ζ: 7.33
Asn^{18}	6.83	4.43	2.88, 2.81	-	δ: n.b.
Leu^{19}	7.05	3.21	1.61	1.61	δ: 1.05-1.02
Lys^{20}	8.11	3.84	1.73-1.60	1.46-1.44	δ: 1.59-1.55
					ε: 2.95
Thr^{21}	7.49	3.78	3.99	1.10	-
His^{22}	7.38	4.17	3.12, 2.91	-	δ^{2}: 7.09
					ε^{1}: 7.96
Val^{23}	8.28	3.47	2.33	1.33, 1.26	-
Lys^{24}	6.86	4.10	1.75-1.64	1.56-1.50	δ: 1.79-1.74

Ile[25]	7.83	3.87	1.59	0.90, 0.80	ε: 2.96<br>$\gamma^{CH}{}_3$: 0.48<br>δ: 0.67
His[26]	7.20	4.39	2.59, 2.06	-	δ^2: 6.40<br>ε^1: 7.95
Thr[27]	7.64	4.32	4.38	1.22	-
Gly[28]	8.02	3.92, 3.71	-	-	-

LC-MS (ESI+): Calcd. for $[C_{145}H_{223}N_{40}O_{39}S_2]$ $[M+3H]^{3+}$: 1071.2050, found: 1071.2056

RP-HPLC: tr = 9.15 min (10% - 40% MeCN in 10min, 0.45 mL/min).

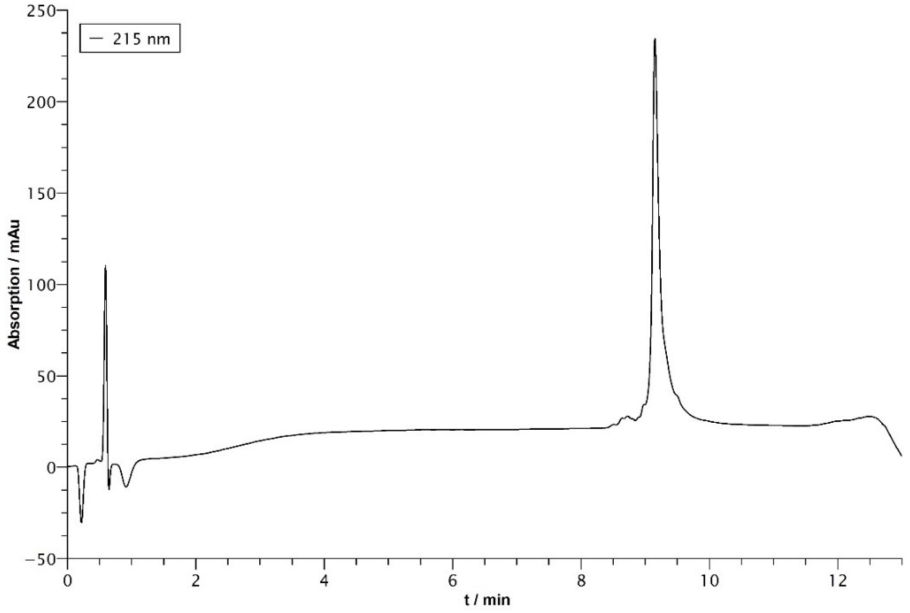

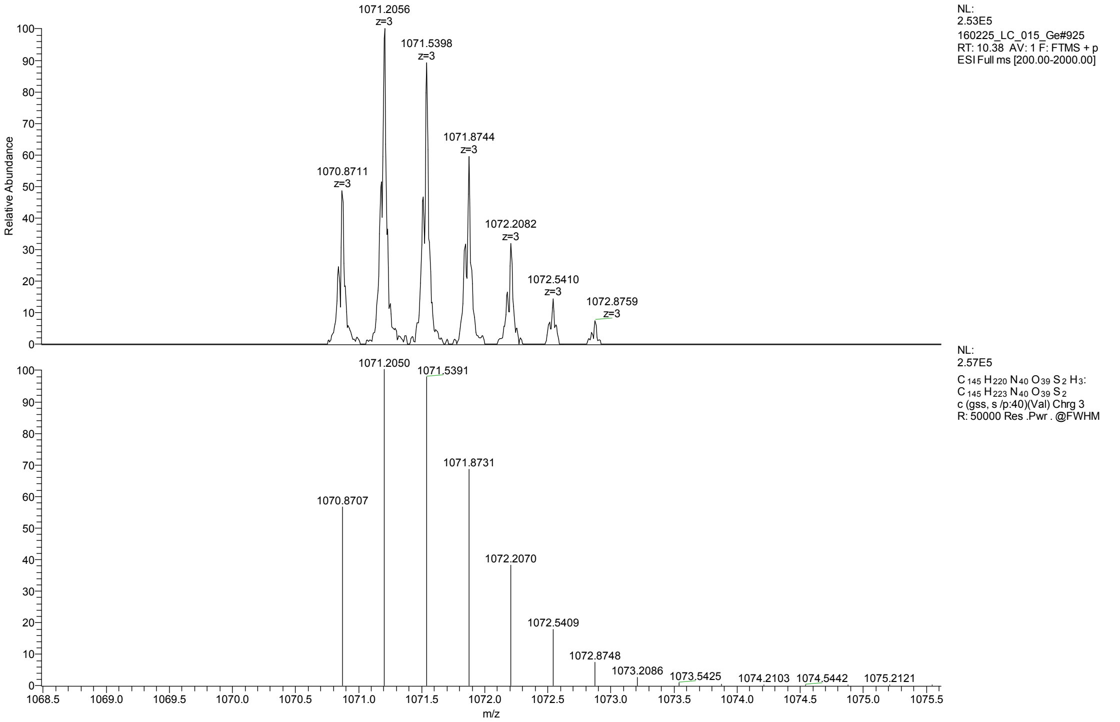

ZFV-1

P^{1}F^{2}Q^{3}C^{4}T^{5}F^{6}**Hot^{7}Tap^{8}**C^{9}G^{10}K^{11}R^{12}F^{13}S^{14}L^{15}D^{16}F^{17}N^{18}L^{19}K^{20}T^{21}H^{22}V^{23}K^{24}I^{25}H^{26}T^{27}G^{28}

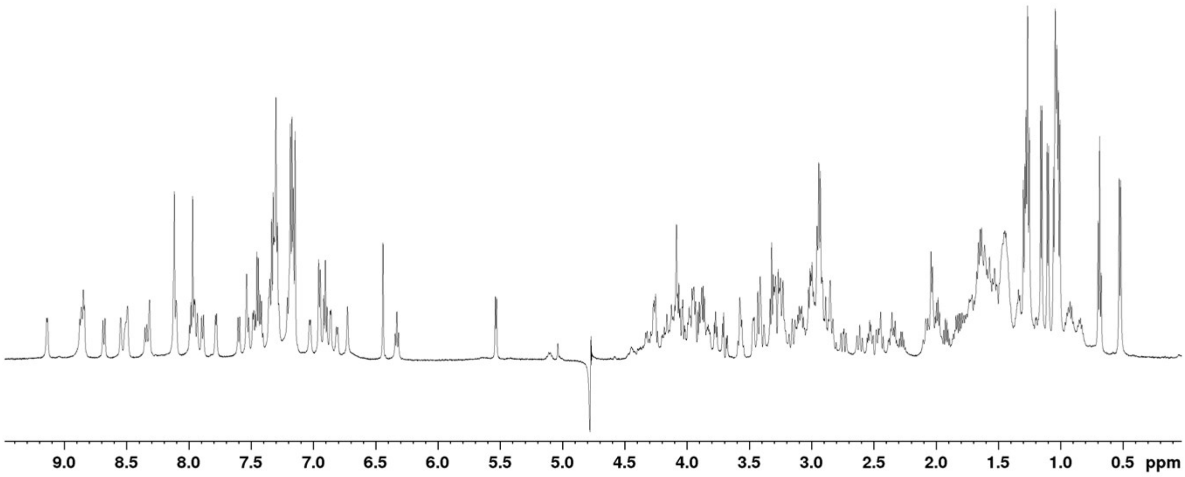

^{1}H-NMR: 600 MHz, 300K, H_2O/D_2O 9:1, 1.5 eq Zn^{2+}, pH = 5.9, n.b = nicht bestimmbar, - = nicht vorhanden.

Amino acid	δ(NH) / ppm	δ(α) / ppm	δ(β) / ppm	δ(γ) / ppm	δ other / ppm
Pro^{1}	n.b.	4.24	2.33, 1.81	1.98, 1.91	3.31
Phe^{2}	n.b.	4.64	3.25, 2.87	-	δ: 7.19
					ε, ζ: 7.46
Gln^{3}	8.86	5.11	1.99, 1.84	2.36, 2.06	ε: n.b.
Cys^{4}	9.14	4.46	3.25, 2.94	-	-
Thr^{5}	8.35	4.62	4.70	1.28	-
Phe^{6}	8.85	4.29	2.54, 2.45	-	δ: 6.95
					ε, ζ: 7.29
Hot^{7}	8.88	3.58	4.09	4.07	δ: 5.04
Tap^{8}	-	5.53	3.43, 3.01	-	-
Cys^{9}	8.13	4.15	4.16, 4.13	-	-
Gly^{10}	8.51	3.96, 3.88	-	-	-
Lys^{11}	7.96	3.94	1.64-1.52	1.46-1.41	δ:1.59-1.46
					ε: 2.94
Arg^{12}	7.89	5.02	1.64, 1.52	1.52, 1.43	δ:3.10
					ε: n.b.
Phe^{13}	8.68	4.79	3.40, 2.61	-	δ: 7.18
					ε: 6.91
					ζ: 6.33
Ser^{14}	7.19	4.58	4.24, 4.21	-	-
Leu^{15}	7.54	4.86	1.66-1.41	1.66-1.41	δ: 1.03
Asp^{16}	8.55	3.42	2.06, 2.00	-	-
Phe^{17}	8.49	4.10	3.27, 2.73	-	δ: 7.15
					ε, ζ: 7.33
Asn^{18}	6.81	4.43	2.87, 2.81	-	δ: n.b.
Leu^{19}	7.03	3.23	2.08, 1.19	1.59	δ: 1.07-1.01
Lys^{20}	8.11	3.83	1.76-1.69	1.44-1.33	δ: 1.76-1.58
					ε: 3.19
Thr^{21}	7.49	3.77	3.99	3.99	-
His^{22}	7.36	4.18	3.16, 2.91	-	δ^{2}: 7.15
					ε^{1}: 7.97

Val[23]	8.32	3.46	2.27	1.27	-
Lys[24]	6.87	4.08	1.76-1.68	1.53-1.43	δ: 1.76-1.66<br>ε: 2.95
Ile[25]	7.79	3.87	1.64	0.94, 0.85	γ^{CH_3}: 0.53<br>δ: 0.69
His[26]	7.20	4.38	2.48, 1.59	-	δ^2: 6.45<br>ε^1: 8.12
Thr[27]	7.60	4.27	4.34	1.16	-
Gly[28]	7.99	3.92, 3.70	-	-	-

HPLC-MS (ESI+): calcd. for $[C_{146}H_{224}N_{40}O_{39}S_3]$ $[M+4H]^{4+}$: 814.6486, found: 814.6481

RP-HPLC: tr = 9.29 min (10% - 40% MeCN in 10min, 0.45 mL/min).

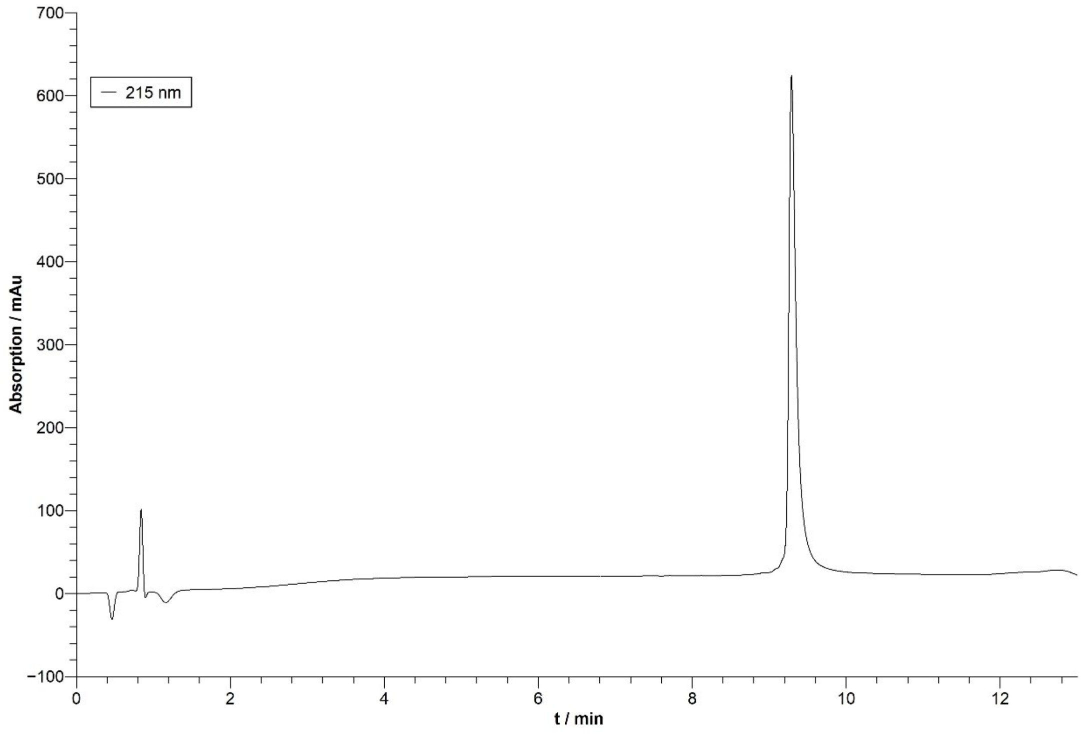

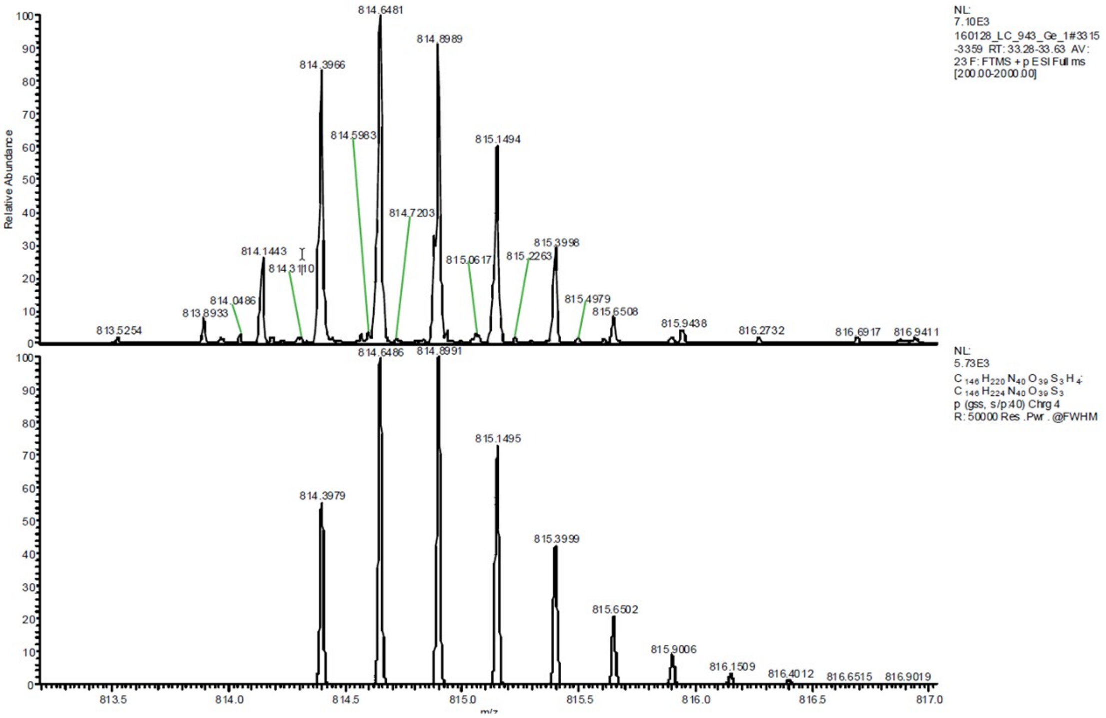

Zinc finger 3YY1 – modified with Y13

P[1]F[2]Q[3]C[4]T[5]F[6]**Hot[7]Tap[8]**C[9]G[10]K[11]R[12]**Y[13]**S[14]L[15]D[16]F[17]N[18]L[19]K[20]T[21]H[22]V[23]K[24]I[25]H[26]T[27]G[28]

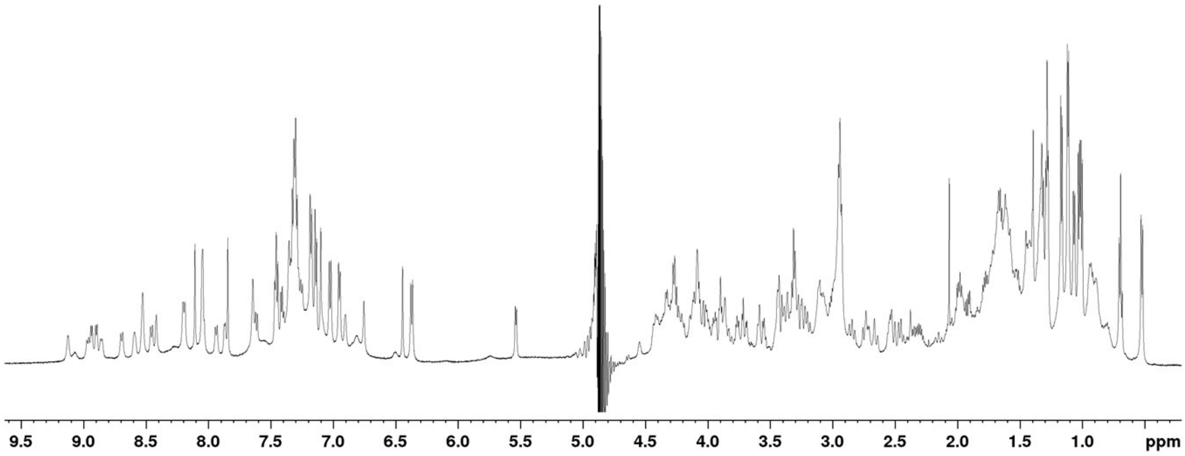

^{1}H-NMR: 600 MHz, 290K, H_2O/D_2O 9:1, 1.5 eq Zn^{2+}, pH = 6.0, n.b = nicht bestimmbar, - = nicht vorhanden.

Amino acid	δ(NH) / ppm	δ(α) / ppm	δ(β) / ppm	δ(γ) / ppm	δ other / ppm
Phe[2]	8.97	4.74	3.23, 2.84	-	δ: 7.18<br>ε, ζ: 7.47
Gln[3]	8.86	5.03	1.98, 1.85	2.38, 2.07	ε: n.b.
Cys[4]	9.13	4.43	3.19, 294	-	-
Thr[5]	8.46	4.64	4.64	1.28	-
Phe[6]	8.90	4.29	2.55, 2.43	-	δ: 6.96<br>ε, ζ: 7.29
Hot[7]	8.94	3.57	4.09	4.07	δ: 5.05
Tap[8]	-	5.54	3.42, 3.01	-	-
Cys[9]	8.19	4.13	3.29	-	-
Gly[10]	8.59	4.02, 3.85	-	-	-

Lys11	8.05	3.92	1.67	1.44, 1.33	δ: 1.57-1.50<br>ε: 2.93
Arg12	7.94	5.06	1.62	1.52, 1.41	δ: 3.08<br>ε: n.b.
Tyr13	8.70	4.74	3.37, 2.53	-	δ: 7.03<br>ε: 6.37
Ser14	n.b.	n.b.	n.b.	-	-
Leu15	7.64	4.82	1.64-1.55	1.39	δ: 1.01
Asp16	8.52	3.44	1.93	-	-
Phe17	8.53	4.11	3.26, 2.74	-	δ: 7.14<br>ε, ζ: 7.36-7.26
Asn18	6.82	4.42	2.76-2.61	-	δ: n.b.
Leu19	7.18	3.09	1.61, 1.52	1.53	δ: 1.39
Lys20	8.21	3.86	1.74	1.46, 1.36	δ: 1.72, 1.56<br>ε: 2.95
Thr21	7.46	3.77	4.00	1.11	-
His22	7.35	4.21	3.37, 3.09	-	δ^2: 7.10<br>ε^1: 7.85
Val23	8.42	3.43	2.29	1.31, 1.29	-
Lys24	6.91	4.08	1.85-1.65	1.58-1.42	δ: 1.85-1.65<br>ε: 2.93
Ile25	7.87	3.90	1.67	0.94, 0.80	γ^{CH_3}: 0.52<br>δ: 0.70
His26	7.25	4.39	2.49, 1.59	-	δ^2: 6.45<br>ε^1: 8.11
Thr27	7.62	4.25	4.31	1.17	-
Gly28	8.04	3.71, 3.92	-	-	-

HPLC-MS (ESI+): calcd. for [$C_{146}H_{225}N_{40}O_{40}S_3$] $[M+5H]^{5+}$: 655.1193, found: 655.1180

RP-HPLC: tr = 8.55 min (10% - 40% MeCN in 10min, 0.45 mL/min).

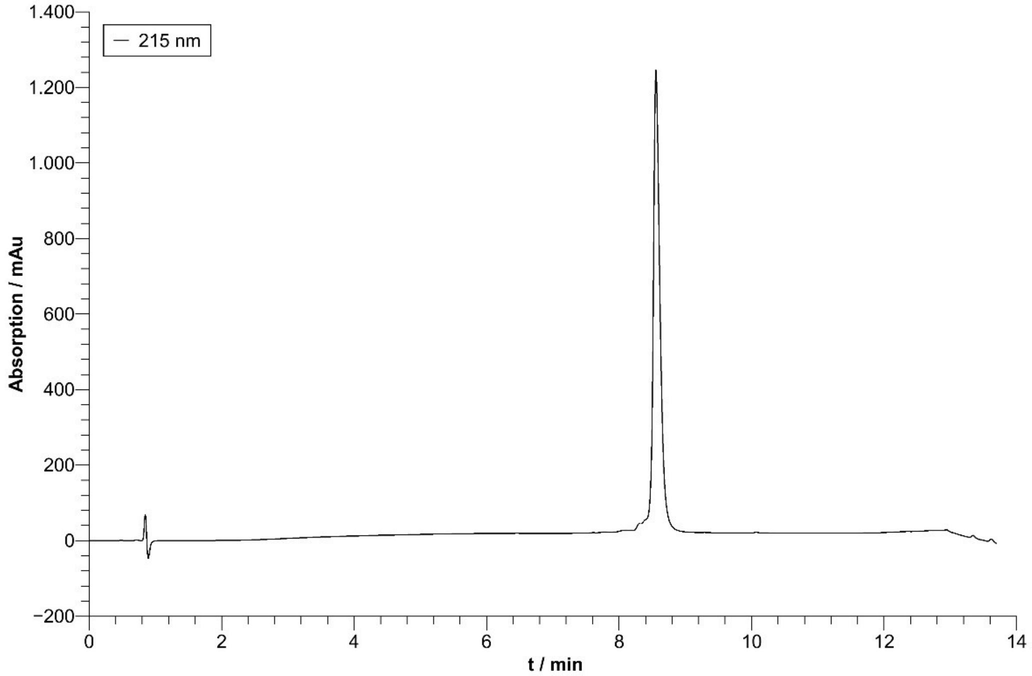

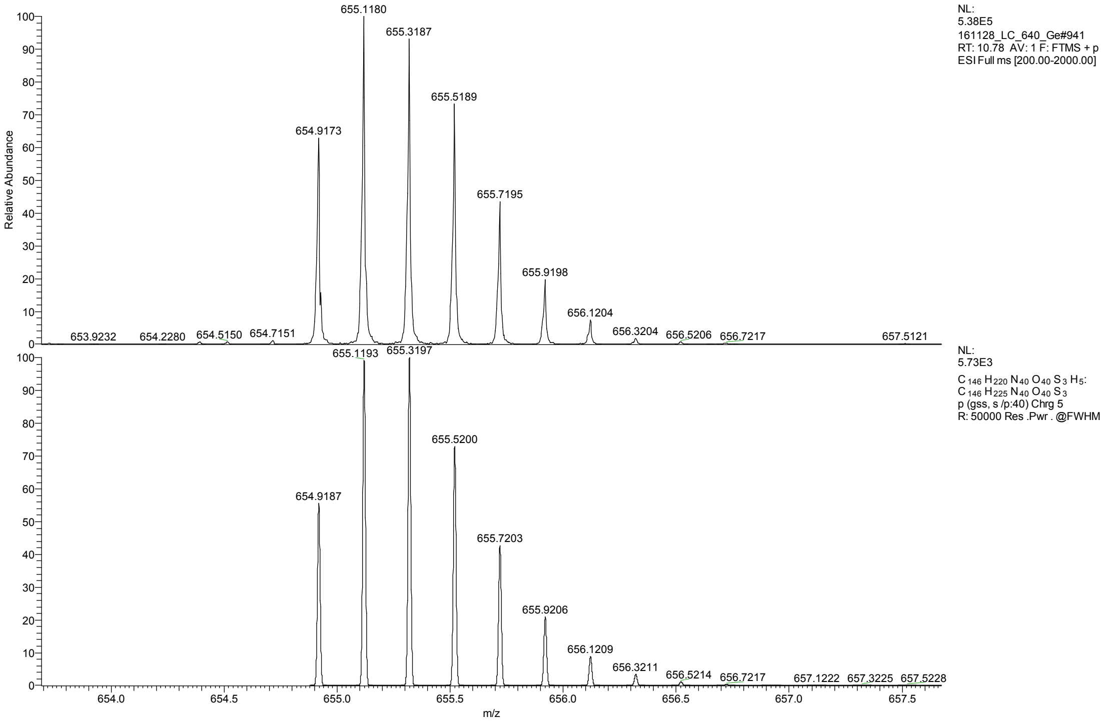

ZFV-4

P[1]F[2]Q[3]C[4]T[5]F[6]**Hot[7]Tap[8]**C[9]G[10]K[11]R[12]**X[13]**S[14]L[15]D[16]F[17]N[18]L[19]K[20]T[21]H[22]V[23]K[24]I[25]H[26]T[27]G[28]

X^{13}:

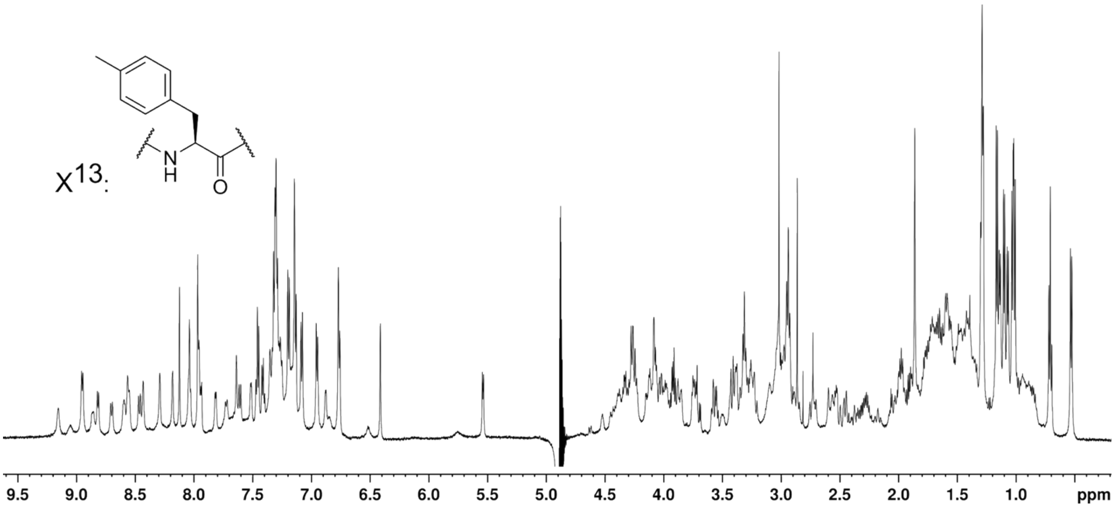

^{1}H-NMR: 600 MHz, 290K, H_2O/D_2O 9:1, 1.5 eq Zn^{2+}, pH = 6.8, n.b = nicht bestimmbar, - = nicht vorhanden.

Amino acid	δ(NH) / ppm	δ(α) / ppm	δ(β) / ppm	δ(γ) / ppm	δ other / ppm
Pro[1]	n.b.	4.26	2.33, 1.78	2.00, 1.91	δ: 3.33
Phe[2]	8.95	4.78	3.25, 2.87	-	δ: 7.20<br>ε, ζ: 7.45
Gln[3]	8.84	5.00	2.00, 1.84	2.38, 2.05	ε: n.b.
Cys[4]	9.14	4.45	3.24, 2.97	-	-
Thr[5]	8.44	4.62	4.64	1.29	-

Phe6	8.82	4.29	2.53, 2.45	-	δ: 6.96<br>ε, ζ: 7.30
Hot7	8.94	3.59	4.10	4.10	δ: 5.06
Tap8	-	5.55	3.43, 3.02	-	-
Cys9	8.17	4.14	3.29	-	-
Gly10	8.59	4.00, 3.87	-	-	-
Lys11	7.95	4.01	1.59	1.47, 1.38	δ: 1.49<br>ε: 2.88
Arg12	7.94	5.08	1.56	1.41	δ: 3.09<br>ε: 7.18
X13	8.70	4.72	3.43, 2.57	-	δ: 7.08<br>ε: 6.77<br>ζ-Me: 1.87
Ser14	n.b.	4.53	4.07, 4.04	-	-
Leu15	7.72	n.b.	1.64	1.44	δ: 1.05
Asp16	8.57	3.51	1.98	-	-
Phe17	8.53	4.12	3.27, 2.75	-	δ: 7.15<br>ε, ζ: 7.32
Asn18	6.86	4.40	2.72, 2.59	-	δ: n.b.
Leu19	7.15	3.74	2.17	1.40	δ: 1.07
Lys20	8.28	3.87	1.76	1.42	δ: 1.58<br>ε: 2.95
Thr21	7.50	3.76	3.98	1.12	-
His22	7.28	4.25	3.35, 2.89	-	δ^2: 7.16<br>ε^1: 7.98
Val23	8.42	3.39	2.27	1.30	-
Lys24	6.88	4.07	1.75	1.55, 1.42	δ: 1.70<br>ε: 2.95
Ile25	7.81	3.94	1.72	0.86	γ^{CH_3}: 0.54<br>δ: 0.71
His26	7.27	4.39	1.72	-	δ^2: 6.42<br>ε^1: 8.13
Thr27	7.61	4.28	4.34	1.17	-
Gly28	8.03	3.92, 3.72	-	-	-

HPLC-MS (ESI+): calcd. for $[C_{147}H_{225}N_{40}O_{39}S_3]$ $[M+3H]^{3+}$: 1090.8682, found: 1090.8692

RP-HPLC: tr = 9.64 min (10% - 40% MeCN in 10min, 0.45 mL/min).

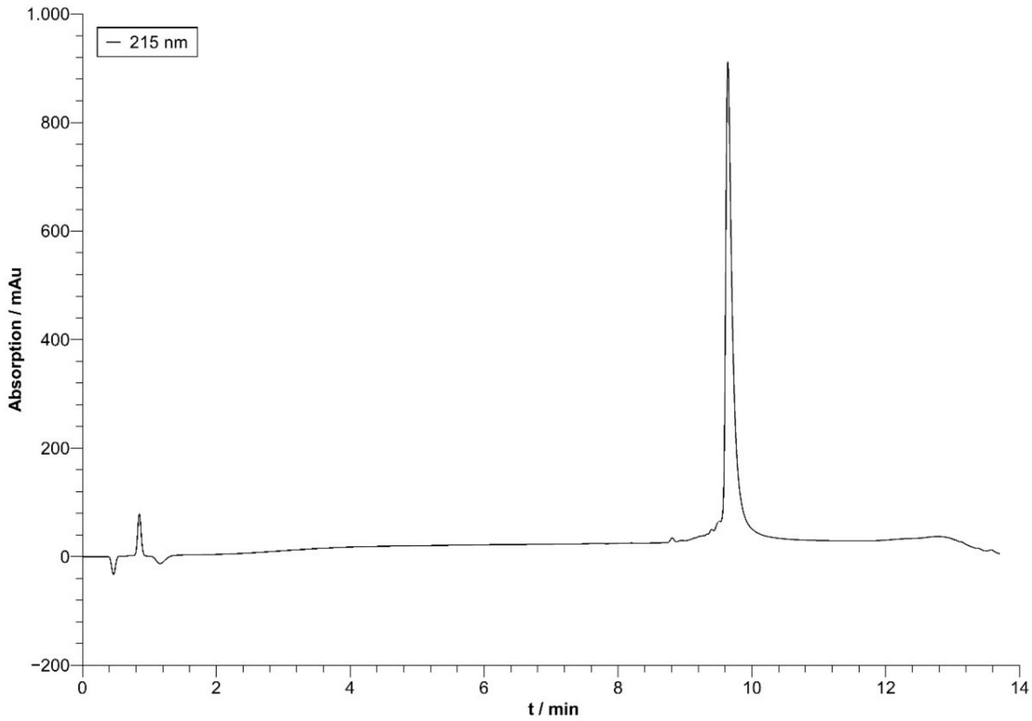

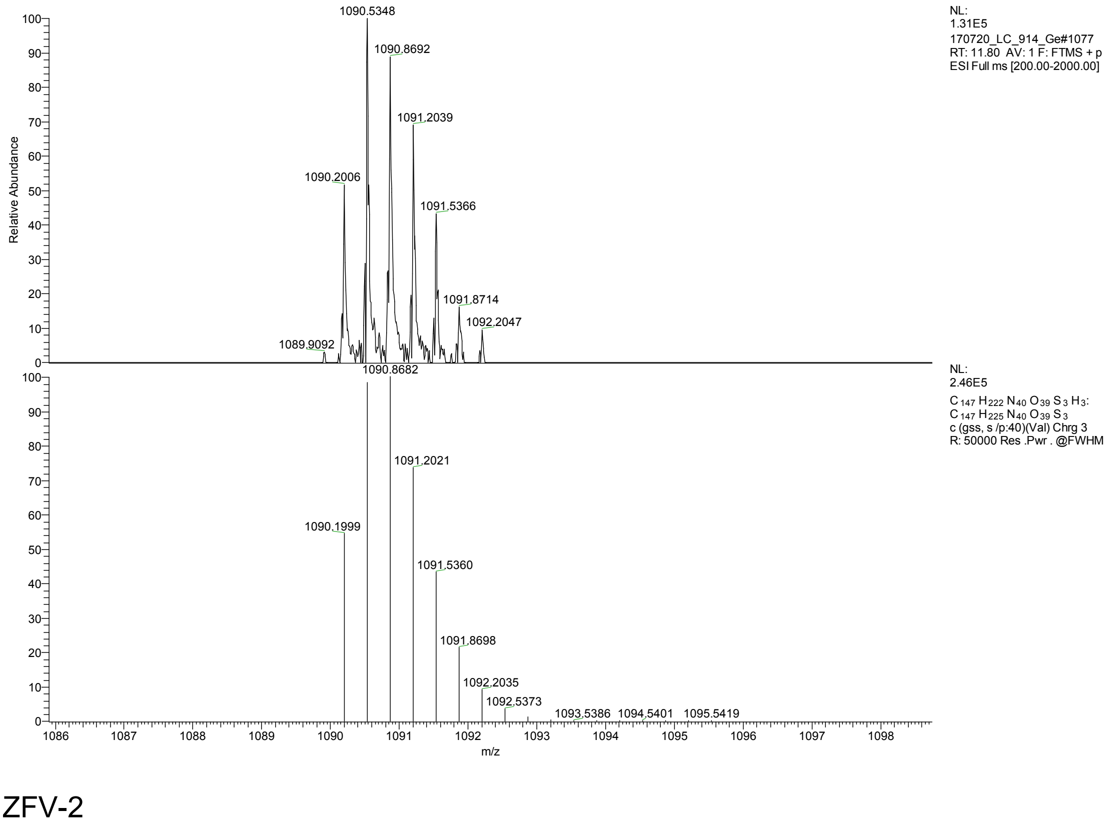

ZFV-2

P^{1}F^{2}Q^{3}C^{4}T^{5}F^{6}**Hot^{7}Tap^{8}**C^{9}G^{10}K^{11}R^{12}**X^{13}**S^{14}L^{15}D^{16}F^{17}N^{18}L^{19}K^{20}T^{21}H^{22}V^{23}K^{24}I^{25}H^{26}T^{27}G^{28}

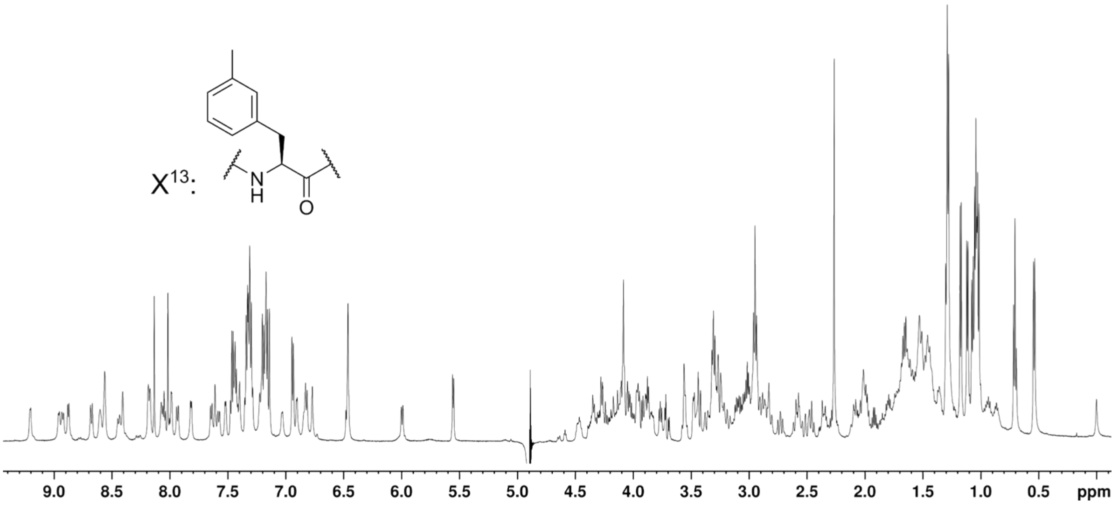

^{1}H-NMR: 600 MHz, 290K, H_2O/D_2O 9:1, 1.5 eq Zn^{2+}, pH = 6.5, n.b = nicht bestimmbar, - = nicht vorhanden.

Amino acid	δ(NH) / ppm	δ(α) / ppm	δ(β) / ppm	δ(γ) / ppm	δ other / ppm
Pro[1]	n.b.	4.24	2.35, 1.97	1.92, 1.80	δ: 3.31
Phe[2]	n.b.	4.81	3.26, 2.91	-	δ: 7.20<br>ε, ζ: n.b.
Gln[3]	8.92	5.11	2.00, 1.85	2.37, 2.08	ε: n.b.
Cys[4]	9.21	4.48	3.27, 2.97	-	-
Thr[5]	8.44	4.64	4.64	1.29	-
Phe[6]	8.88	4.30	2.58, 2.46	-	δ: 6.94<br>ε, ζ: 7.31
Hot[7]	8.96	3.56	4.09	4.09	δ: 5.06
Tap[8]	-	5.56	3.43, 3.01	-	-
Cys[9]	8.19	4.16	3.31	-	-
Gly[10]	8.60	3.98, 3.89	-	-	-
Lys[11]	7.99	3.96	1.51	1.37, 1.27	δ: 1.45<br>ε: 3.03
Arg[12]	7.93	5.03	1.65	1.52, 1.44	δ: 3.09<br>ε: n.b.
X[13]	8.68	4.79	3.37, 2.61	-	δ^1: 7.18<br>δ^2: 6.82<br>ε^1-Me: 2.26<br>ε^2: 6.47<br>ζ: 6.00
Ser[14]	n.b.	4.60	4.07	-	-
Leu[15]	7.58	4.87	1.68, 1.62	1.46	δ: 1.04
Asp[16]	8.57	3.45	2.03	-	-
Phe[17]	8.56	4.12	3.29, 2.73	-	δ: 7.17<br>ε, ζ: 7.33
Asn[18]	6.84	4.47	2.87, 2.84	-	δ: 7.61, 6.77
Leu[19]	7.03	3.23	2.10, 1.23	1.62	δ: 1.07, 1.04
Lys[20]	8.17	3.84	1.73	1.35	δ: 1.57, 1.47<br>ε: 2.95
Thr[21]	7.52	3.77	4.03	1.12	-
His[22]	7.44	4.19	3.20, 2.85	-	δ^2: 7.14<br>ε^1: 8.02
Val[23]	8.41	3.48	2.28	1.29	-
Lys[24]	6.91	4.10	1.76	1.56, 1.42	δ: 1.67<br>ε: 2.95

Ile[25]	7.82	3.88	1.66	0.94, 0.87	γ^{CH_3}: 0.54<br>δ: 0.71
His[26]	7.22	4.37	2.52, 1.52	-	δ^2: 6.46<br>ε^1: 8.14
Thr[27]	7.64	4.27	4.35	1.18	-
Gly[28]	8.05	3.93, 3.71	-	-	-

HPLC-MS (ESI+): calcd. for [$C_{147}H_{225}N_{40}O_{39}S_3$] $[M+3H]^{3+}$: 1090.8684, found: 1090.8720

RP-HPLC: tr = 8.78 min (10% - 40% MeCN in 10min, 0.45 mL/min).

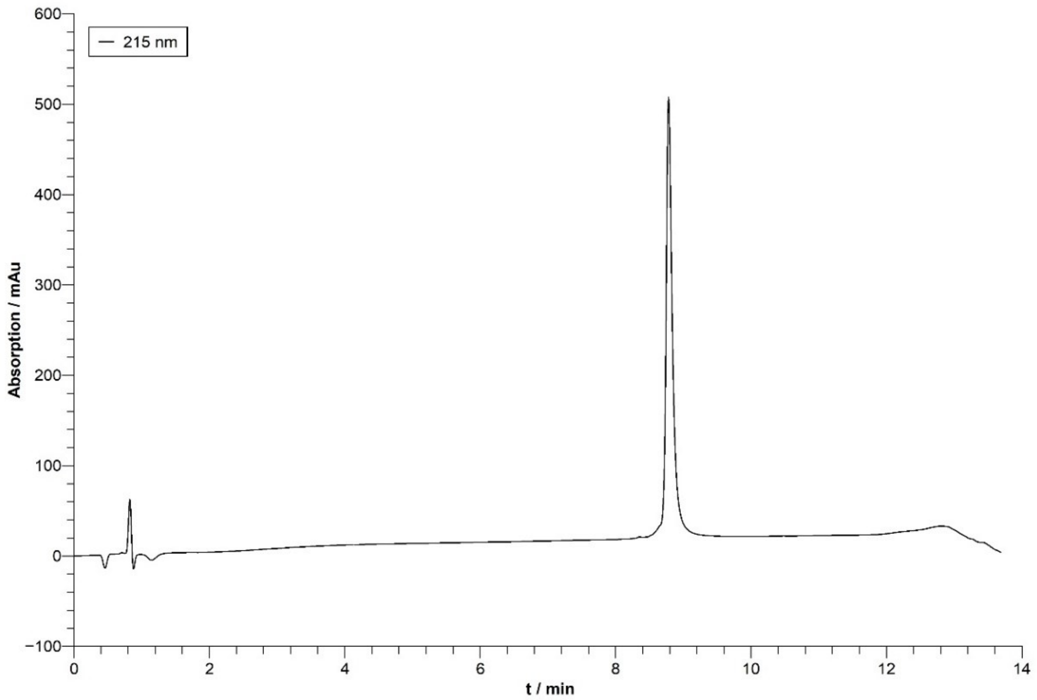

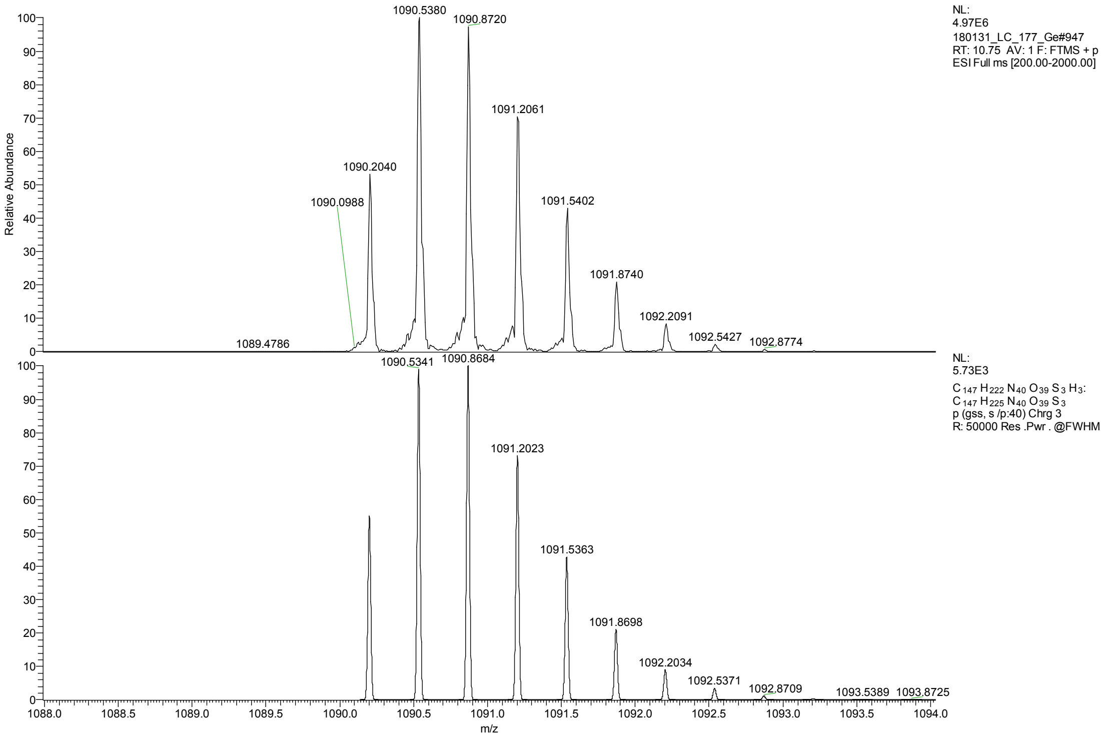

ZFV-3

P^{1}F^{2}Q^{3}C^{4}T^{5}F^{6}**Hot^{7}Tap^{8}**C^{9}G^{10}K^{11}R^{12}**X^{13}**S^{14}L^{15}D^{16}F^{17}N^{18}L^{19}K^{20}T^{21}H^{22}V^{23}K^{24}I^{25}H^{26}T^{27}G^{28}

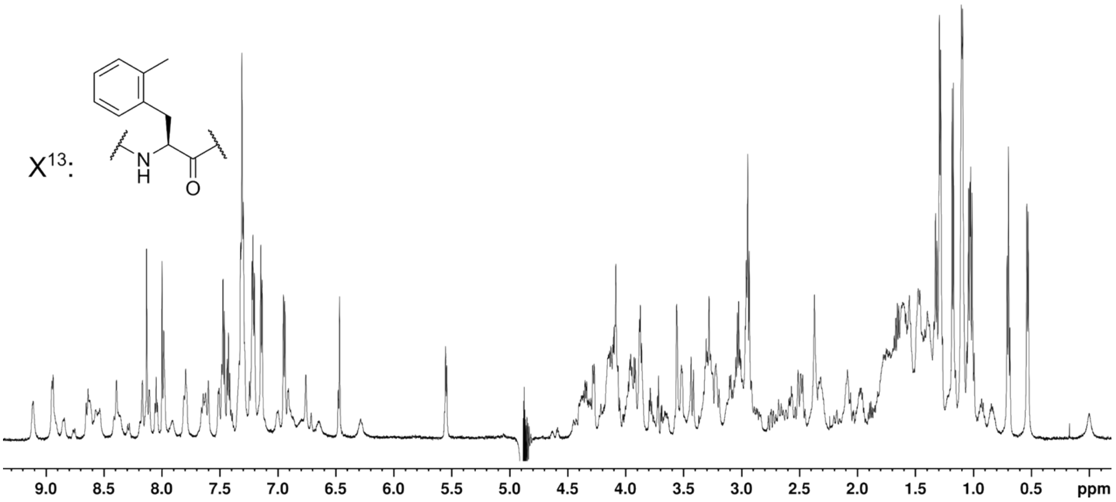

^{1}H-NMR: 600 MHz, 300K, H_2O/D_2O 9:1, 1.5 eq Zn^{2+}, pH = 6.5, n.b = nicht bestimmbar, - = nicht vorhanden.

Amino acid	δ(NH) / ppm	δ(α) / ppm	δ(β) / ppm	δ(γ) / ppm	δ other / ppm
Pro^{1}	n.b.	4.24	2.24, 1.98	1.90, 1.80	δ: 3.30
Phe^{2}	n.b.	n.b.	n.b.	-	δ: 7.21
					ε, ζ: 7.46
Gln^{3}	8.77	5.06	1.97, 1.84	2.35, 2.06	ε: n.b.
Cys^{4}	9.05	4.43	3.20, 2.94	-	-
Thr^{5}	8.27	4.62	4.62	1.29	-
Phe^{6}	8.92	4.31	2.55, 2.48	-	δ: 6.97
					ε, ζ: 7.32
Hot^{7}	8.87	3.58	4.08	4.09	δ: 5.05
Tap^{8}	-	5.54	3.43, 3.02	-	-
Cys^{9}	8.11	4.15	3.28	-	-
Gly^{10}	8.56	3.96, 3.88	-	-	-
Lys^{11}	7.94	3.93	1.41	1.21	δ: 1.55
					ε: 3.03
Arg^{12}	7.74	5.03	1.62, 1.50	1.42	δ: 3.09
					ε: n.b.
X^{13}	8.62	4.99	3.49, 2.63	-	δ^{1}-Me: 2.35
					δ^{2}: 7.01
					ε^{1}: 6.79
					ε^{2}: 6.67
					ζ: 6.31
Ser^{14}	n.b.	4.60	4.08	-	-
Leu^{15}	7.62	4.88	1.62	1.47	δ: 1.03
Asp^{16}	8.25	3.10	n.b.	-	-
Phe^{17}	8.45	4.16	3.29, 2.77	-	δ: 7.15
					ε, ζ: 7.31
Asn^{18}	6.89	4.38	2.66, 2.49	-	δ: 7.53, 6.73
Leu^{19}	7.15	3.66	2.15, 1.69	1.34	δ: 1.09
Lys^{20}	8.05	3.88	1.76	1.44	δ: 1.66
					ε: 2.96
Thr^{21}	7.49	3.81	3.99	1.10	-
His^{22}	7.49	4.19	3.22, 2.88	-	δ^{2}: 7.24
					ε^{1}: 7.99
Val^{23}	8.34	3.53	2.32	1.32, 1.27	-

Lys[24]	6.90	4.11	1.76	1.49	δ: 1.67<br>ε: 2.96
Ile[25]	7.76	3.88	1.64	0.94, 0.84	γ^{CH_3}: 0.54<br>δ: 0.70
His[26]	7.20	4.40	2.50, 1.63	-	δ^2: 6.47<br>ε^1: 8.13
Thr[27]	7.63	4.28	4.34	1.17	-
Gly[28]	8.00	3.93, 3.71	-	-	-

HPLC-MS (ESI+): calcd. for [$C_{147}H_{225}N_{40}O_{39}S_3$] $[M+3H]^{3+}$: 1090.8682, found: 1090.8678

RP-HPLC: tr = 8.79 min (10% - 40% MeCN in 10min, 0.45 mL/min).

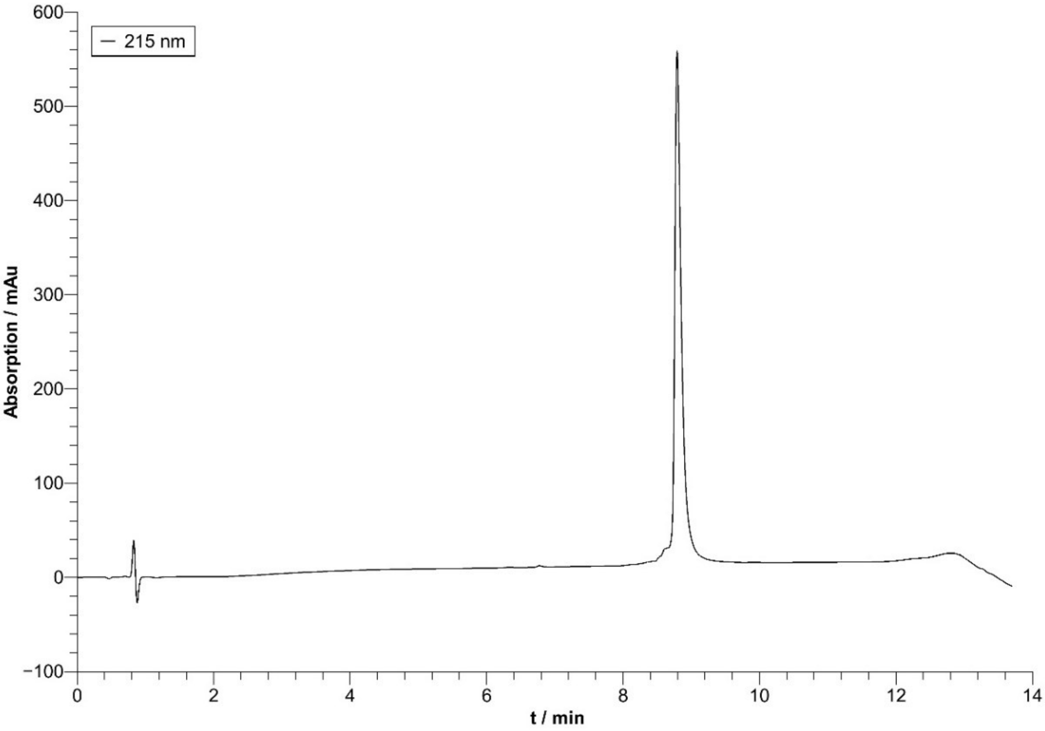

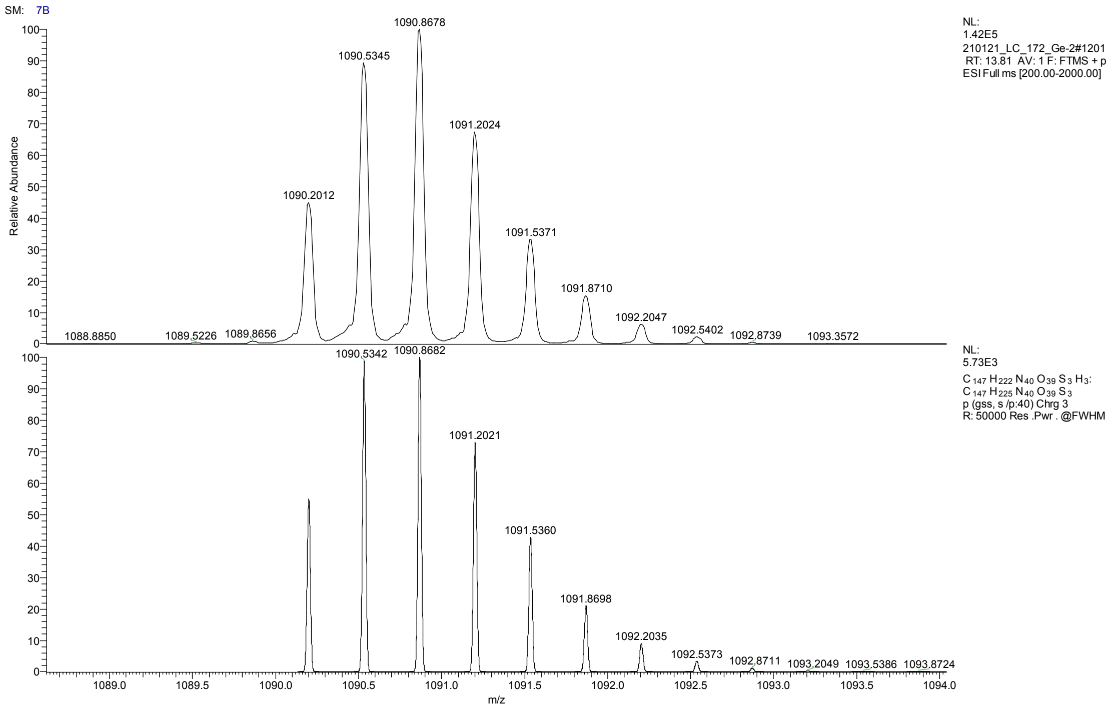

ZFV-6

P[1]F[2]Q[3]C[4]T[5]F[6]**Hot[7]Tap[8]**C[9]G[10]K[11]R[12]**X[13]**S[14]L[15]D[16]F[17]N[18]L[19]K[20]T[21]H[22]V[23]K[24]I[25]H[26]T[27]G[28]

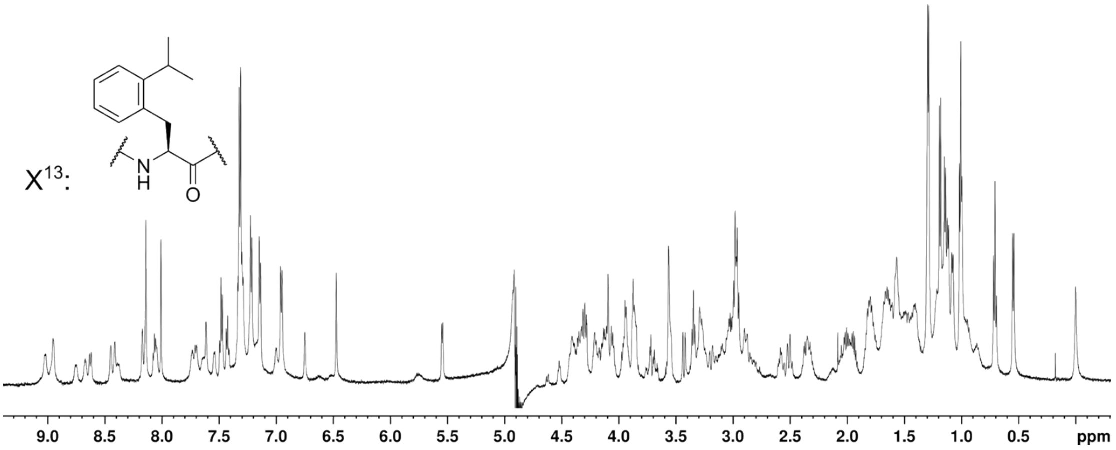

The substitution of F13 with FID leads, after the Addition of 1.5 eq Zinc to a defined structure with characteristic zinc finger-type signals. Unfortunately, the signals from L15-N18 were not detectable due to line broadening. Still 70% of the signals could be assigned.

^{1}H-NMR: 600 MHz, 290K, H_2O/D_2O 9:1, 1.5 eq Zn^{2+}, pH = 6.5, n.d = not detectable, - = nicht vorhanden.

Amino acid	δ(NH) / ppm	δ(α) / ppm	δ(β) / ppm	δ(γ) / ppm	δ other / ppm
Pro^1	n.b.	4.32	2.37, 2.01	1.95, 1.81	δ: 3.35
Phe^2	8.97	4.74	3.20, 2.88	-	δ: 7.22<br>ε, ζ: 8.75
Gln^3	8.75	4.99	1.91	2.36, 2.05	ε: n.b.
Cys^4	9.02	4.43	3.16, 2.91	-	-
Thr^5	8.38	4.63	4.63	1.29	-
Phe^6	9.02	4.33	2.60, 2.51	-	δ: 6.96<br>ε, ζ: 7.33
Hot^7	8.95	3.57	4.10	4.09	δ: 5.07
Tap^8	-	5.55	3.43, 3.02	-	-
Cys^9	8.17	4.16	3.29	-	-
Gly^{10}	8.67	3.96, 3.87	-	-	-
Lys^{11}	8.04	3.93	1.58	1.45, 1.34	δ: 1.67<br>ε: 2.95
Arg^{12}	7.64	5.09	1.57	1.47, 1.40	δ: 3.07<br>ε: n.b.
X^{13}	8.63	5.01	3.71, 2.68	-	δ^1-*i*Pr: n.b.<br>δ^2: n.b.<br>ε^1, ε^2, ζ: n.b.
Ser^{14}	n.b.	4.52	4.09	-	-
Leu^{15}	n.b.	n.b.	n.b.	n.b.	δ: n.b.
Asp^{16}	n.b.	n.b.	n.b.	-	-
Phe^{17}	n.b.	n.b.	n.b.	-	δ, ε, ζ: n.b.
Asn^{18}	n.b.	n.b.	n.b.	-	δ: n.b.
Leu^{19}	7.17	3.09	1.57	1.48	δ: 1.40
Lys^{20}	8.14	3.95	1.81	1.41	δ: 1.63<br>ε: 2.99
Thr^{21}	7.54	3.87	4.06	1.15	-
His^{22}	n.b.	n.b.	3.17, 2.90	-	δ^2: 7.29<br>ε^1: 8.01

Val[23]	8.41	3.55	2.33	1.30	-
Lys[24]	7.00	4.13	1.78	1.56	δ: 1.68<br>ε: 2.97
Ile[25]	7.74	3.88	1.64	0.96, 0.87	γ^{CH_3}: 0.55<br>δ: 0.71
His[26]	7.23	4.42	2.53, 1.59	-	δ^2: 6.48<br>ε^1: 8.15
Thr[27]	7.70	4.30	4.36	1.19	-
Gly[28]	8.07	3.95, 3.72	-	-	-

HPLC-MS (ESI+): calcd. for $[C_{149}H_{229}N_{40}O_{39}S_3]$ $[M+3H]^{3+}$: 1100.2120, found: 100.2138

RP-HPLC: tr = 8.14 min (10% - 50% MeCN in 10min, 0.45 mL/min).

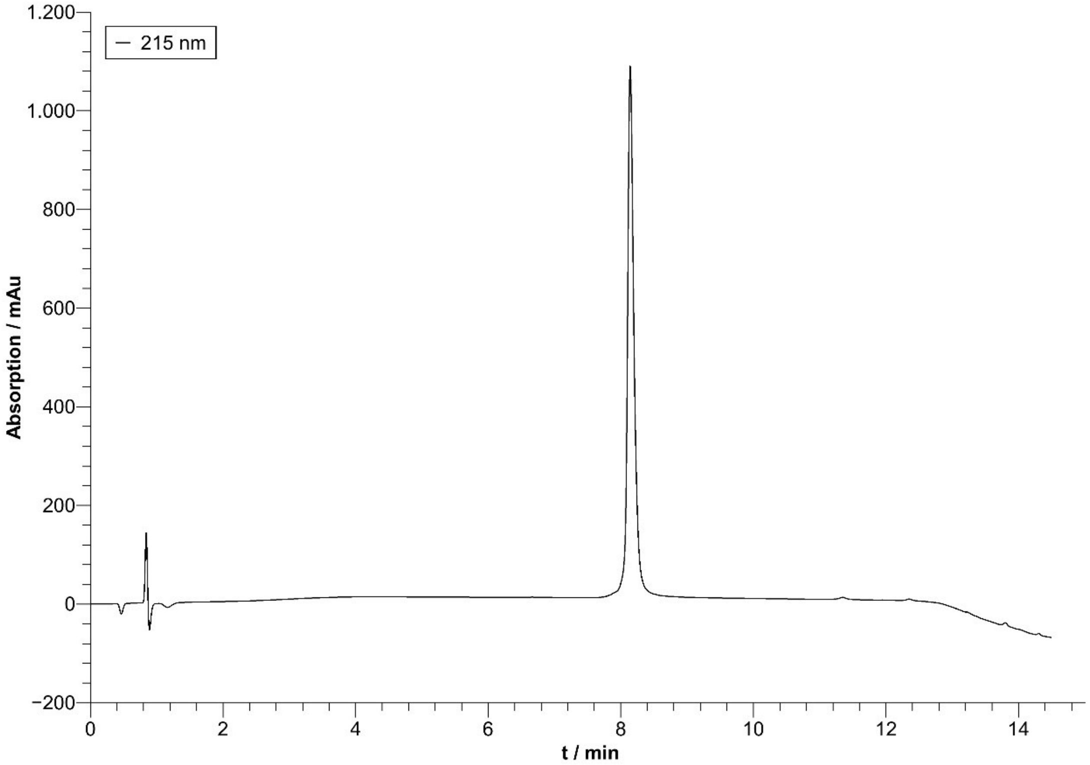

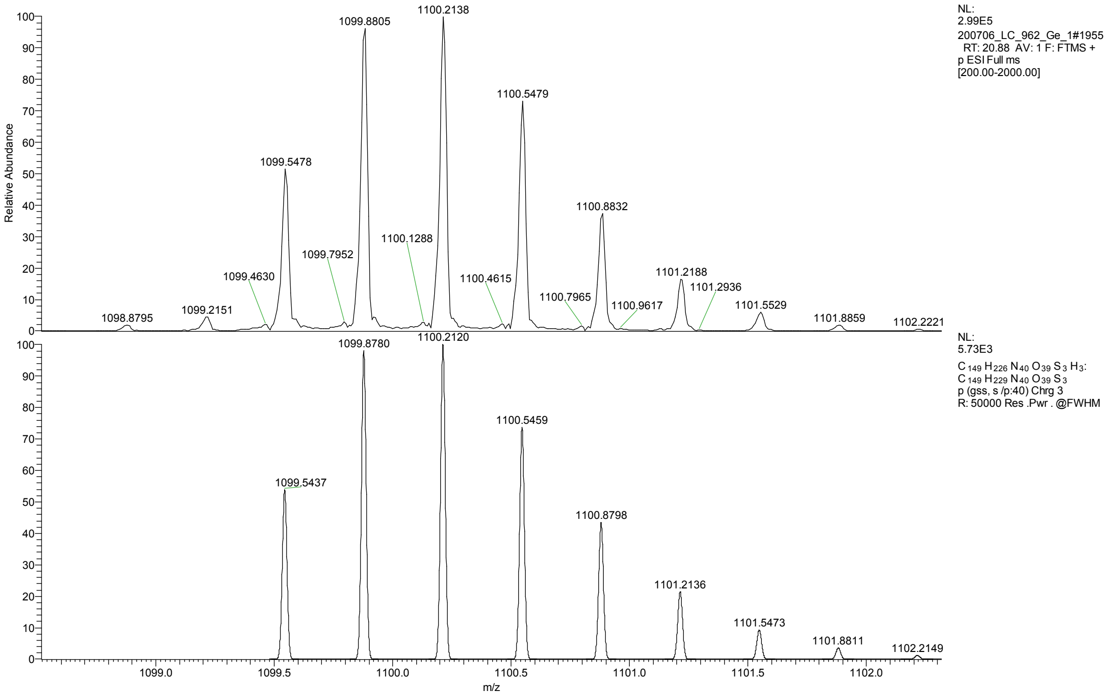

ZFV-5

P^{1}F^{2}Q^{3}C^{4}T^{5}F^{6}**Hot^{7}Tap^{8}**C^{9}G^{10}K^{11}R^{12}**X^{13}**S^{14}L^{15}D^{16}F^{17}N^{18}L^{19}K^{20}T^{21}H^{22}V^{23}K^{24}I^{25}H^{26}T^{27}G^{28}

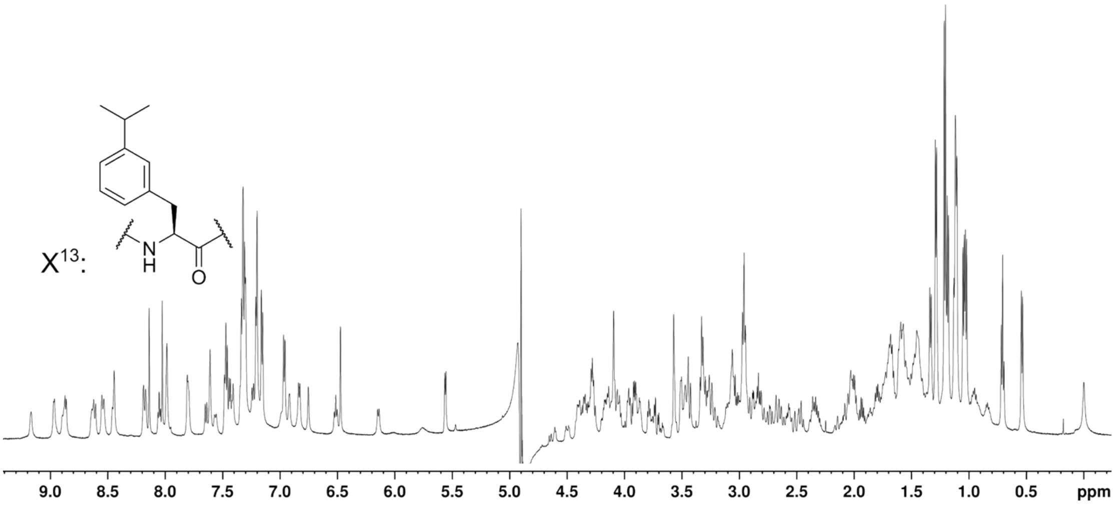

^{1}H-NMR: 600 MHz, 290K, H_2O/D_2O 9:1, 1.5 eq Zn^{2+}, pH = 6.5, n.b = nicht bestimmbar, - = nicht vorhanden.

Amino acid	δ(NH) / ppm	δ(α) / ppm	δ(β) / ppm	δ(γ) / ppm	δ other / ppm
Pro^{1}	n.b.	4.27	2.35, 1.99	1.96, 1.80	δ: 3.33
Phe^{2}	n.b.	4.78	3.25, 2.92	-	δ: 7.21<br>ε, ζ: 7.46
Gln^{3}	8.89	5.02	2.01, 1.86	2.36, 2.07	ε: n.b.
Cys^{4}	9.17	4.50	3.26, 2.96	-	-
Thr^{5}	8.45	4.65	4.65	1.27	-
Phe^{6}	8.87	4.31	2.57, 2.46	-	δ: 6.97<br>ε, ζ: 7.32
Hot^{7}	8.97	3.57	4.10	4.09	δ: 5.06
Tap^{8}	-	5.56	3.43, 3.02	-	-
Cys^{9}	8.19	4.16	3.31	-	-

Gly^{10}	8.64	3.98, 3.89	-	-	-
Lys^{11}	7.99	3.96	1.44	1.23	δ: 1.54<br>ε: 3.06
Arg^{12}	7.80	4.96	1.60	1.49, 1.41	δ: 3.08<br>ε: n.b.
X^{13}	8.61	4.87	3.46, 2.64	-	δ^{1}: 7.30<br>δ^{2}: 6.83<br>ε^{1}-CH: 2.83<br>ε^{1}-*i*Pr: 1.21<br>ε^{2}: 6.52<br>ζ: 6.15
Ser^{14}	n.b.	4.61	4.08	-	-
Leu^{15}	7.56	4.14	1.67, 1.59	1.03	δ: 1.03
Asp^{16}	8.55	3.46	2.04	-	-
Phe^{17}	8.54	4.14	3.29, 2.74	-	δ: 7.16<br>ε, ζ: 7.33
Asn^{18}	6.84	4.41	2.81, 2.67	-	δ: n.b.
Leu^{19}	6.99	3.49	2.14	1.70	δ: 1.34, 1.12
Lys^{20}	8.17	3.87	1.74	1.48, 1.36	δ: 1.58<br>ε: 2.96
Thr^{21}	7.48	3.79	4.04	1.11	-
His^{22}	7.61	4.18	3.21, 2.85	-	δ^{2}: 7.20<br>ε^{1}: 8.03
Val^{23}	8.45	3.51	2.32	1.30	-
Lys^{24}	6.92	4.10	1.75	1.56, 1.43	δ: 1.68<br>ε: 2.95
Ile^{25}	7.81	3.91	1.69	0.96, 0.83	γ^{CH_3}: 0.54<br>δ: 0.71
His^{26}	7.24	4.39	2.52, 1.56	-	δ^{2}: 6.48<br>ε^{1}: 8.14
Thr^{27}	7.65	4.28	4.35	1.18	-
Gly^{28}	8.05	3.94, 3.72	-	-	-

HPLC-MS (ESI+): calcd. for [$C_{149}H_{229}N_{40}O_{39}S_3$] $[M+3H]^{3+}$: 1100.2120, found: 1100.2364

RP-HPLC: tr = 8.24 min (10% - 50% MeCN in 10min, 0.45 mL/min).

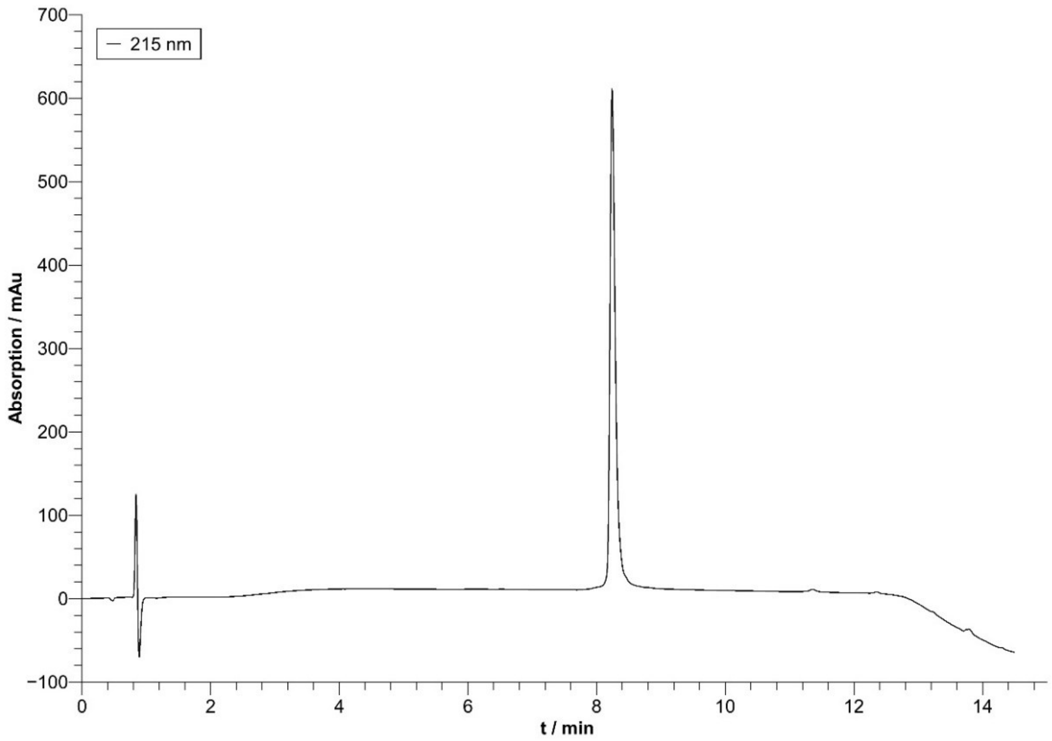

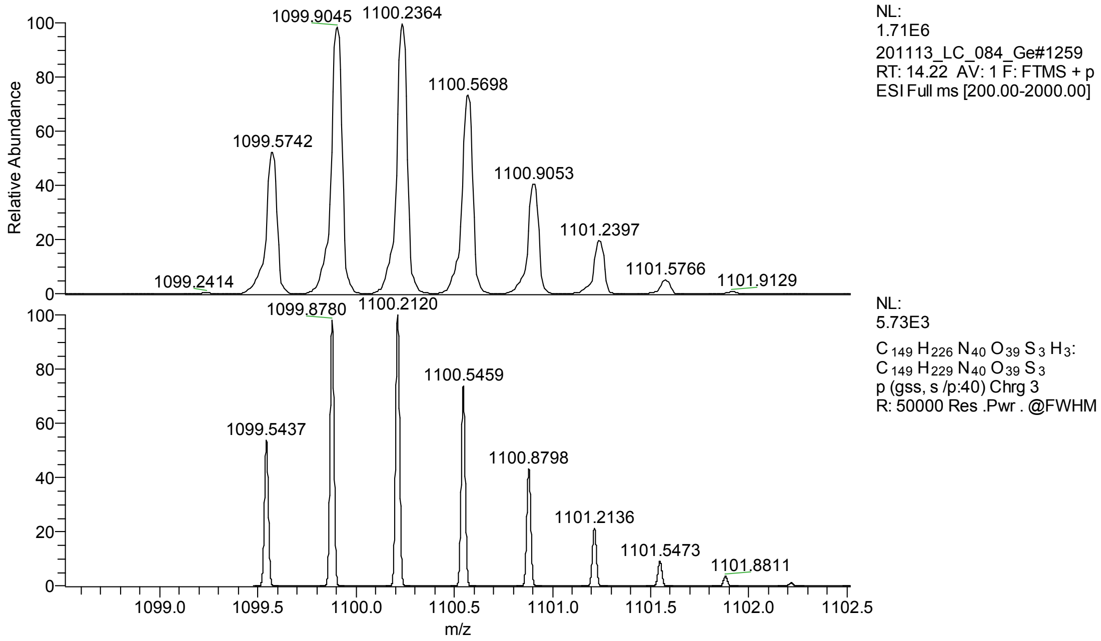

ZFV-7

$P^{1}F^{2}Q^{3}C^{4}T^{5}F^{6}$**Hot**$^{\mathbf{7}}$**Tap**$^{\mathbf{8}}$$C^{9}G^{10}K^{11}R^{12}$**X**$^{\mathbf{13}}$$S^{14}L^{15}D^{16}F^{17}N^{18}L^{19}K^{20}T^{21}H^{22}V^{23}K^{24}I^{25}H^{26}T^{27}G^{28}$

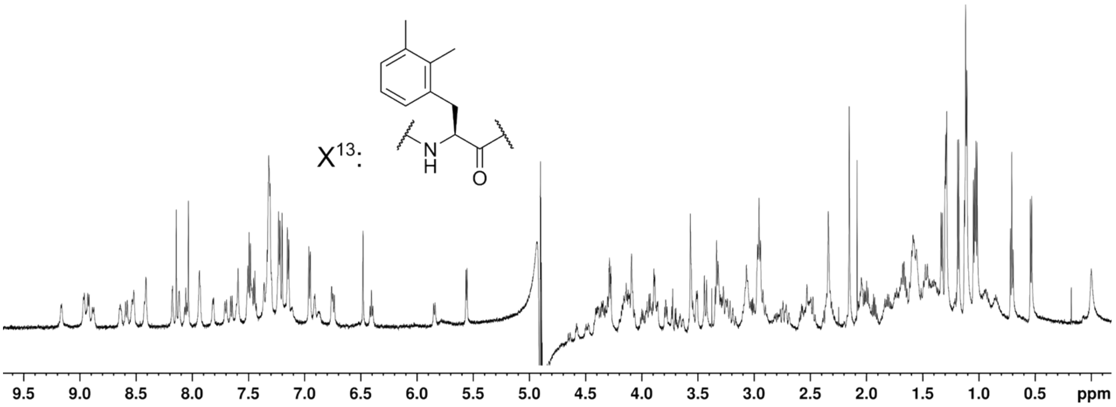

^{1}H-NMR: 600 MHz, 290K, H_2O/D_2O 9:1, 1.5 eq Zn^{2+}, pH = 6.9, n.b = nicht bestimmbar, - = nicht vorhanden.

Amino acid	δ(NH) / ppm	δ(α) / ppm	δ(β) / ppm	δ(γ) / ppm	δ other / ppm
Pro^{1}	n.b.	4.28	2.36, 1.99	1.93, 1.81	δ: 3.33
Phe^{2}	8.95	4.78	3.25, 2.92	-	δ: 7.22
					ε, ζ: 7.48
Gln^{3}	8.87	5.07	1.98, 1.84	2.35, 2.07	ε: n.b.
Cys^{4}	9.16	4.48	3.23, 2.96	-	-
Thr^{5}	8.41	4.64	4.72	1.29	-
Phe^{6}	8.92	4.31	2.58, 2.47	-	δ: 6.95
					ε, ζ: 7.31
Hot^{7}	8.96	3.56	4.09	4.08	δ: 5.05
Tap^{8}	-	5.55	3.43, 3.01	-	-
Cys^{9}	8.17	4.15	3.30	-	-
Gly^{10}	8.63	3.96, 3.88	-	-	-
Lys^{11}	7.93	3.92	1.56	1.20	δ: 1.38
					ε: 3.06
Arg^{12}	7.69	4.99	1.59	1.43	δ: 3.07
					ε: n.b.
X^{13}	8.58	4.99	3.64, 2.53	-	$δ^1$-Me: 2.33
					$ε^1$-Me: 2.15
					$δ^2$: 6.74
					$ε^2$: 6.40
					ζ: 5.84
Ser^{14}	n.b.	4.58	4.11	-	-
Leu^{15}	7.59	n.b.	1.69, 1.61	1.46	δ: 1.03
Asp^{16}	8.52	3.42	2.04	-	-
Phe^{17}	8.53	4.15	3.30, 2.74	-	δ: 7.14
					ε, ζ: 7.31
Asn^{18}	6.87	4.40	2.71, 2.51	-	δ: 7.58, 6.75
Leu^{19}	7.11	3.55	2.17	1.68	δ: 1.34, 1.11
Lys^{20}	8.11	3.87	1.74	1.48, 1.37	δ: 1.58
					ε: 2.96
Thr^{21}	7.50	3.78	3.99	1.11	-
His^{22}	7.47	4.18	3.18, 2.80	-	$δ^2$: 7.20
					$ε^1$: 8.03
Val^{23}	8.41	3.51	2.31	1.31	-
Lys^{24}	6.91	4.10	1.75	1.51	δ: 1.68
					ε: 2.95
Ile^{25}	7.81	3.89	1.66	0.93, 0.85	$γ^{CH_3}$: 0.53
					δ: 0.71
His^{26}	7.23	4.38	2.51, 1.56	-	$δ^2$: 6.48

					ε[1]: 8.14
Thr[27]	7.65	4.28	4.34	1.18	-
Gly[28]	8.05	3.93, 3.71	-	-	-

HPLC-MS (ESI+): calcd. for [$C_{148}H_{227}N_{40}O_{39}S_3$] $[M+3H]^{3+}$: 1095.5401, found: 1095.5529

RP-HPLC: tr = 7.94 min (10% - 50% MeCN in 10min, 0.45 mL/min).

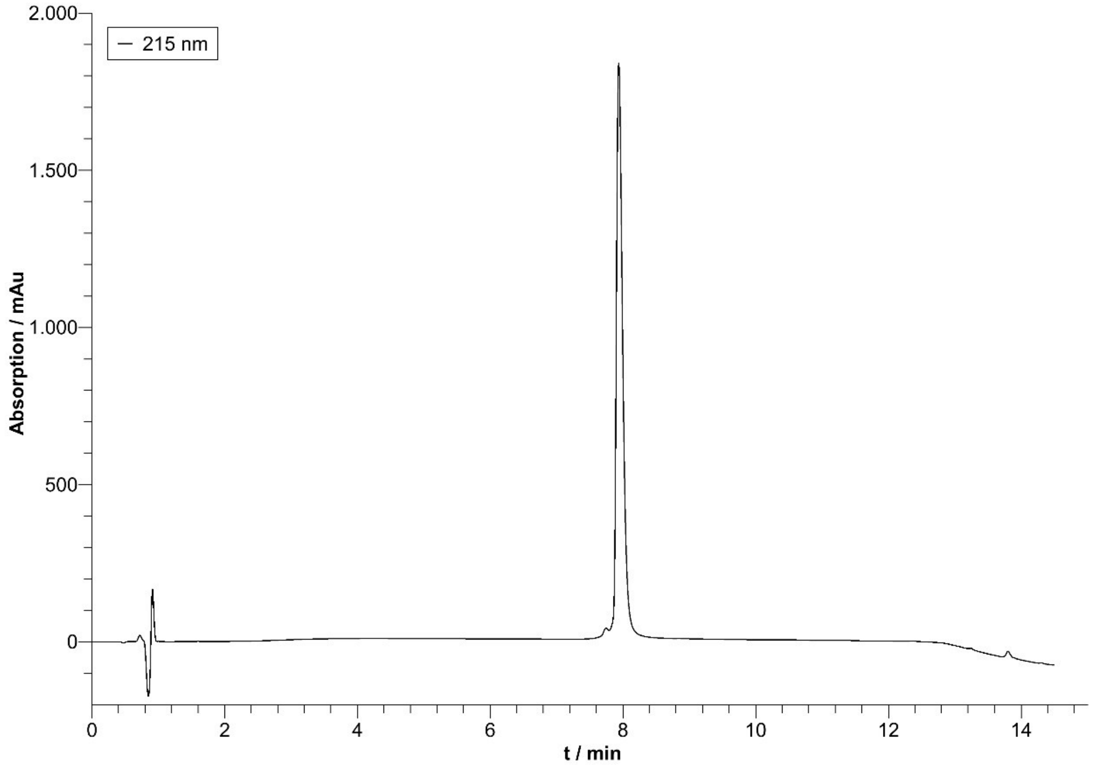

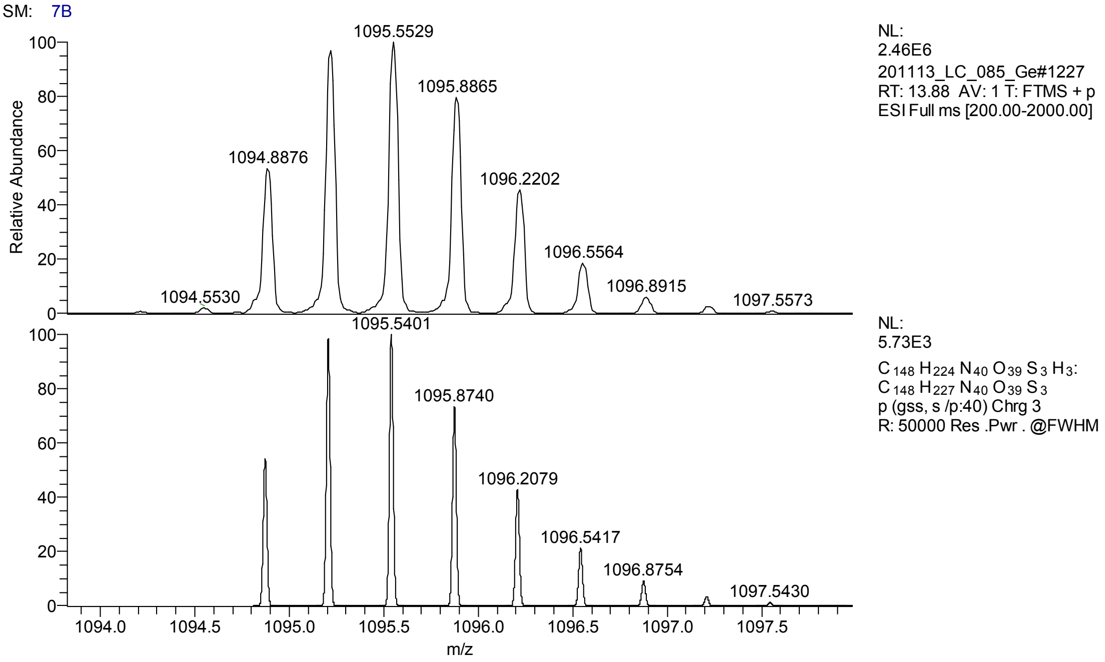

ZFV-8

P^{1}F^{2}Q^{3}C^{4}T^{5}F^{6}**Hot^{7}Tap^{8}**C^{9}G^{10}K^{11}R^{12}**X^{13}**S^{14}L^{15}D^{16}F^{17}N^{18}L^{19}K^{20}T^{21}H^{22}V^{23}K^{24}I^{25}H^{26}T^{27}G^{28}

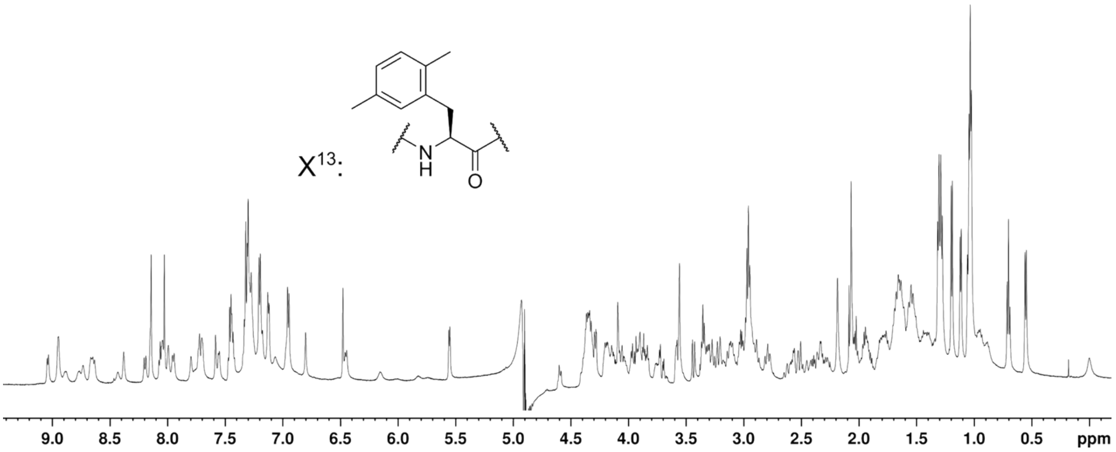

^{1}H-NMR: 600 MHz, 290K, H_2O/D_2O 9:1, 1.5 eq Zn^{2+}, pH = 6.8, n.b = nicht bestimmbar, - = nicht vorhanden.

Amino acid	δ(NH) / ppm	δ(α) / ppm	δ(β) / ppm	δ(γ) / ppm	δ other / ppm
Pro^{1}	n.b.	4.32	2.40, 2.02	1.93	δ: 3.35
Phe^{2}	8.89	4.81	3.23, 2.90	-	δ: 7.21<br>ε, ζ: 7.45
Gln^{3}	8.77	5.08	1.95, 1.83	2.36, 2.06	ε: n.b.
Cys^{4}	8.95	4.36	3.14, 2.94	-	-
Thr^{5}	8.20	4.60	4.72	1.31	-
Phe^{6}	9.04	4.34	2.57, 2.52	-	δ: 6.96<br>ε, ζ: 7.32
Hot^{7}	8.95	3.56	4.10	4.09	δ: 5.07
Tap^{8}	-	5.56	3.44, 3.03	-	-
Cys^{9}	8.16	4.16	3.28	-	-
Gly^{10}	8.67	3.91	-	-	-
Lys^{11}	8.00	3.97	1.53	1.30	δ: 1.37<br>ε: 2.97
Arg^{12}	7.95	5.09	1.68, 1.66	1.54, 1.45	δ: 3.10<br>ε: 7.22
X^{13}	8.64	n.b.	3.19, 2.79	-	δ^{1}-Me: 2.07<br>δ^{2}: 7.07<br>ε^{1}: 6.16<br>ε^{2}-Me: 2.19<br>ζ: 6.46
Ser^{14}	n.b.	4.61	4.07	-	-
Leu^{15}	7.77	n.b.	1.66, 1.56	1.34	δ: 1.03
Asp^{16}	n.b.	n.b.	n.b.	-	-
Phe^{17}	8.43	4.21	3.29, 2.80	-	δ: 7.13<br>ε, ζ: 7.28
Asn^{18}	7.05	4.38	2.62	-	δ: n.b.
Leu^{19}	7.32	3.76	2.09, 1.35	1.68	δ: 1.04
Lys^{20}	8.05	3.90	1.78	1.50, 1.39	δ: 1.62<br>ε: 2.97
Thr^{21}	7.56	3.84	4.03	1.12	-
His^{22}	7.70	4.20	3.23, 2.92	-	δ^{2}: 7.30<br>ε^{1}: 8.03
Val^{23}	8.38	3.59	2.32	1.29	-
Lys^{24}	6.97	4.13	1.78, 1.69	1.44	δ: 1.54<br>ε: 2.96

Ile[25]	7.74	3.87	1.64	0.95, 0.89	γ^{CH_3}: 0.55<br>δ: 0.71
His[26]	7.19	4.40	2.53, 1.59	-	δ^2: 6.48<br>ε^1: 8.15
Thr[27]	7.72	4.29	4.36	1.20	-
Gly[28]	8.07	3.95, 3.72	-	-	-

HPLC-MS (ESI+): calcd. for $[C_{148}H_{227}N_{40}O_{39}S_3]$ $[M+3H]^{3+}$: 1095.8741, found: 1095.8835

RP-HPLC: tr = 8.01 min (10% - 50% MeCN in 10min, 0.45 mL/min).

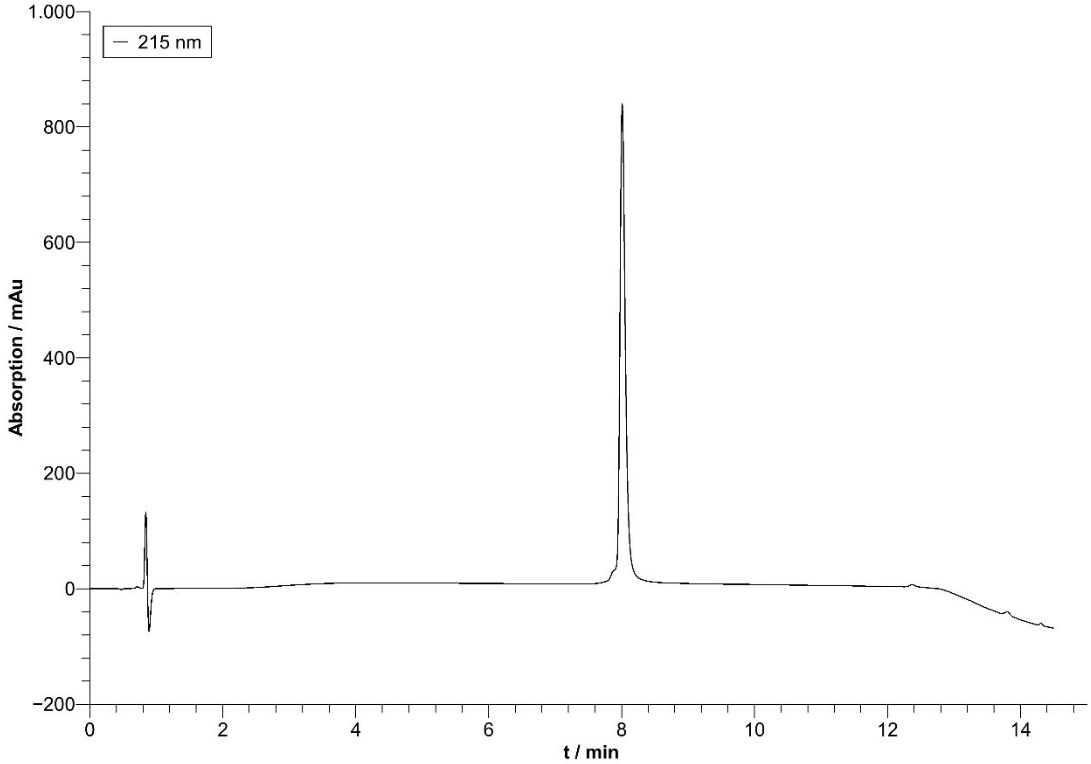

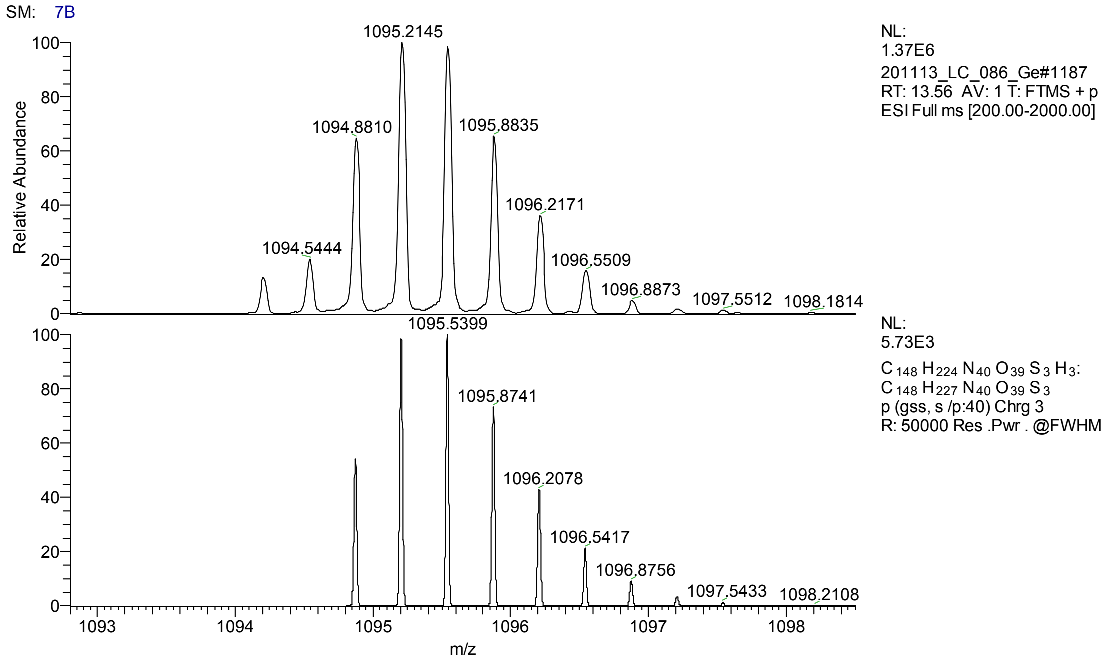

Variable temperature NMR

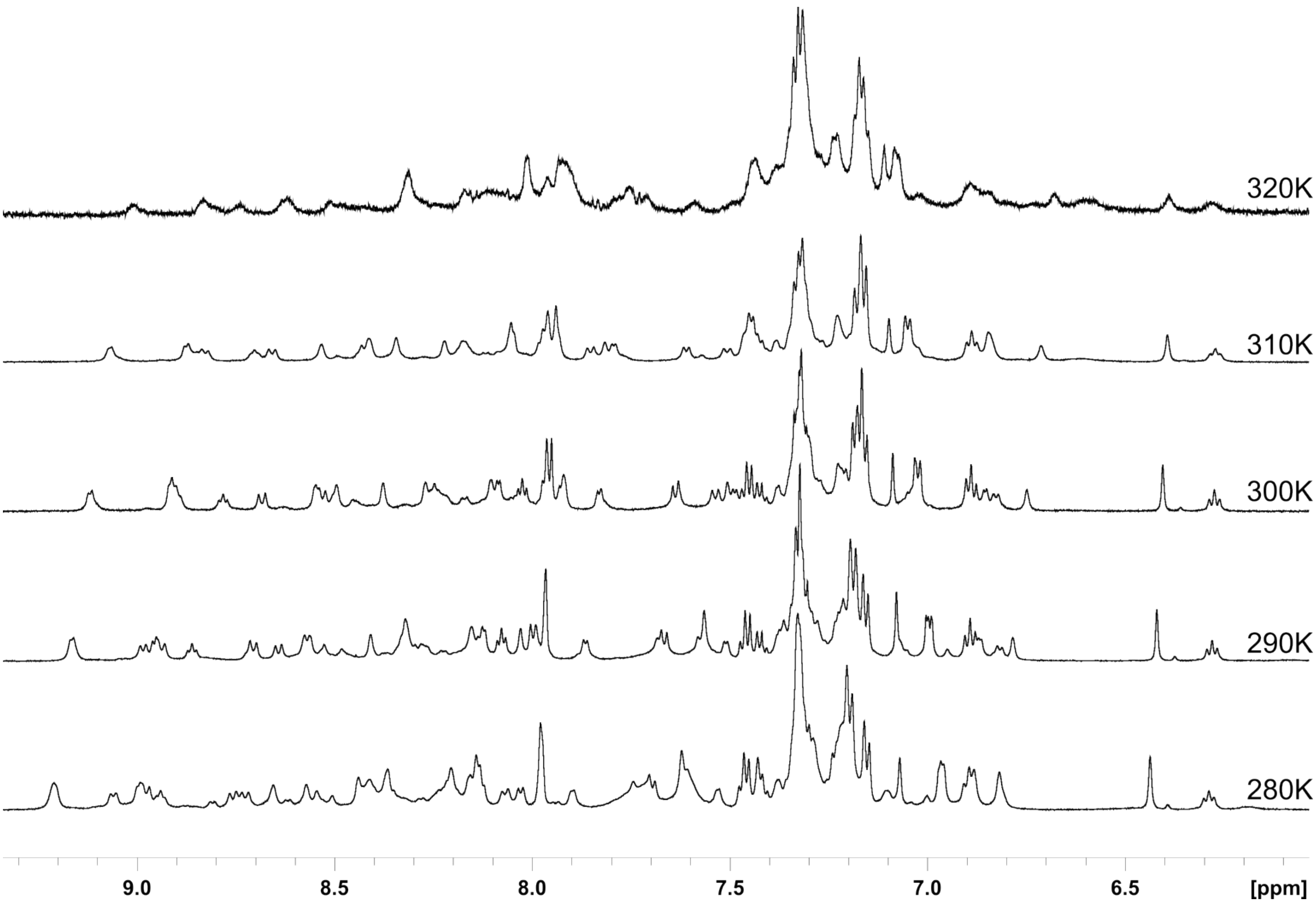

Figure S6: Section of the 1H-NMR of 3YY1, measured at different temperatures.

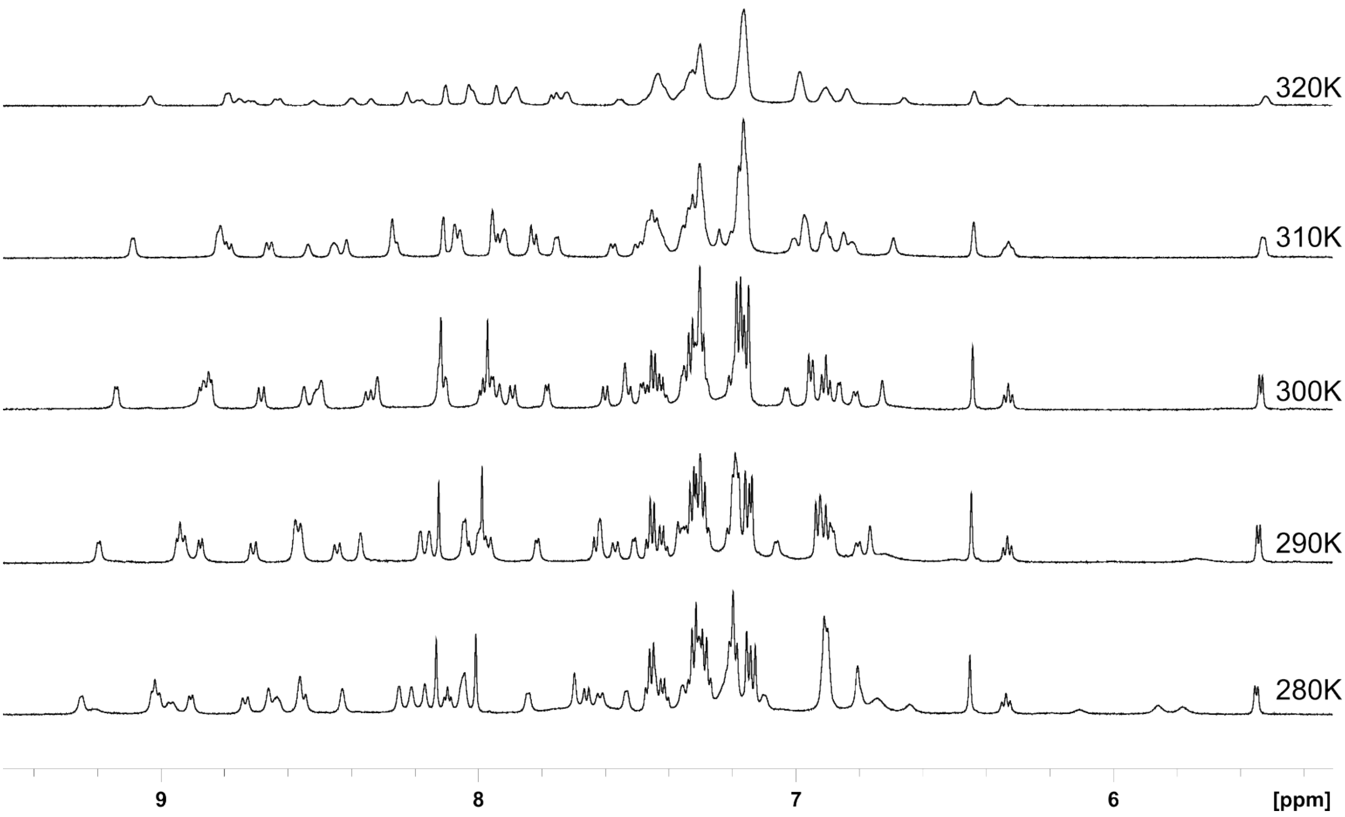

Figure S7: Section of the 1H-NMR of ZFV-1, measured at different temperatures.

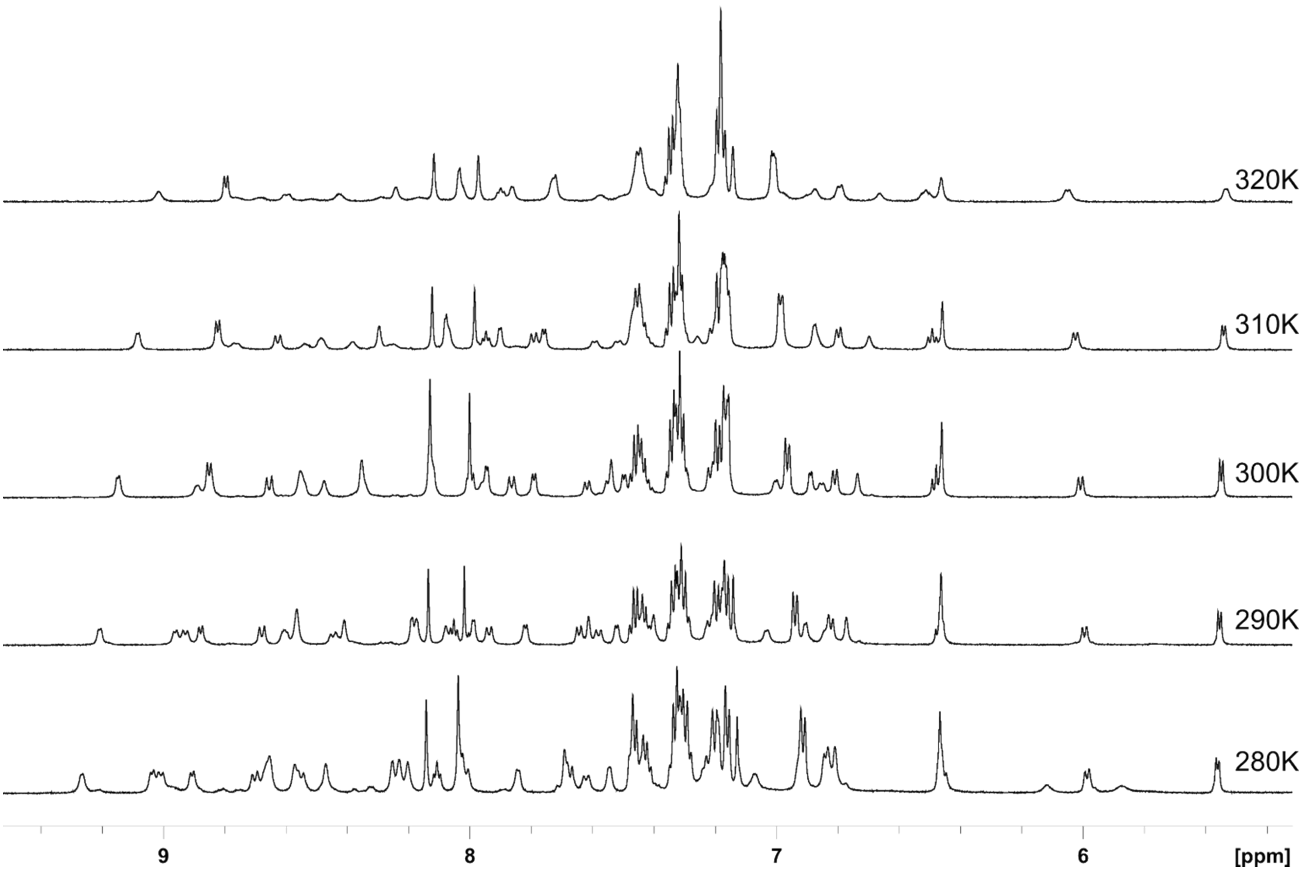

Figure S8: Section of the 1H-NMR of ZFV-2, measured at different temperatures.

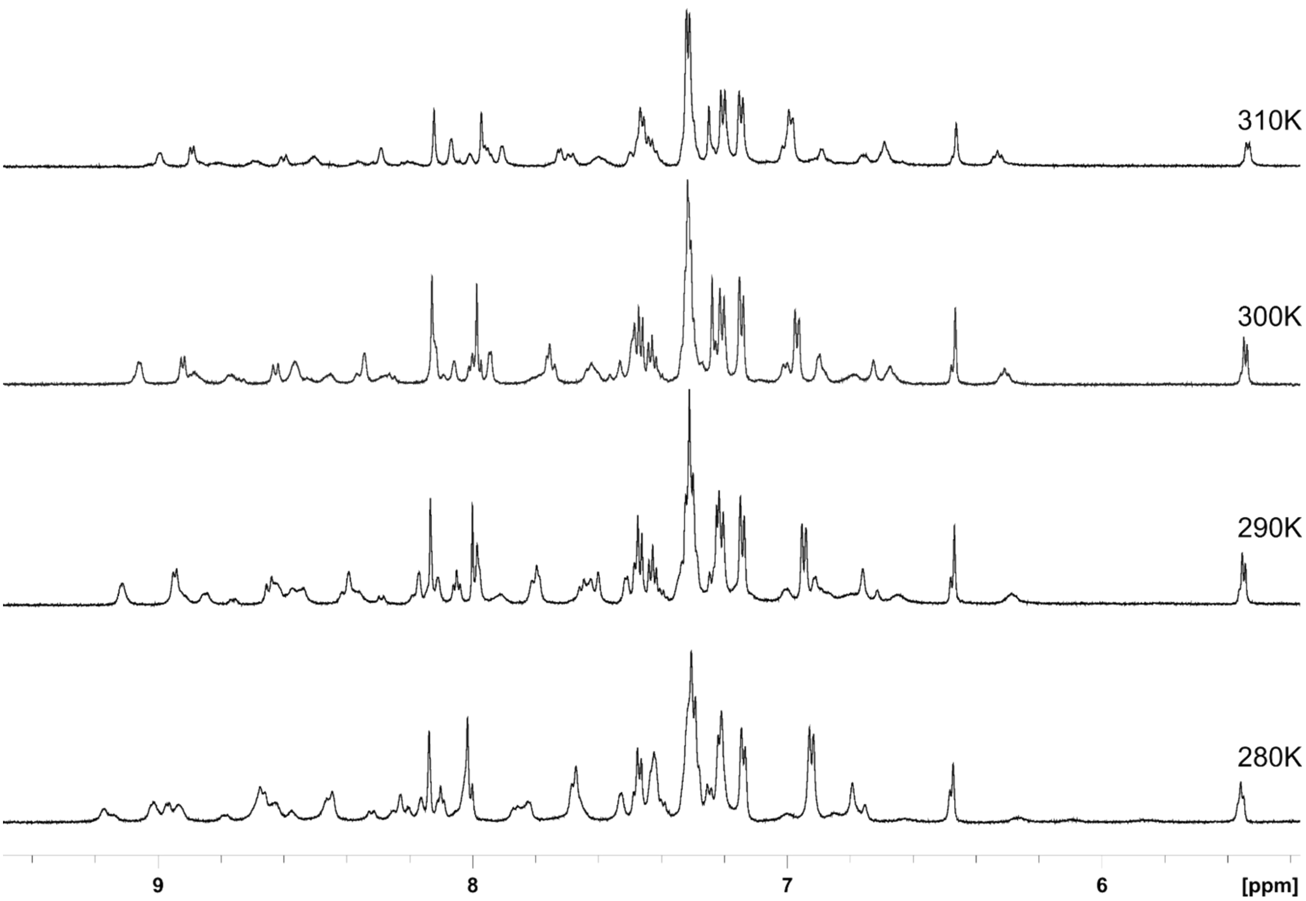

Figure S9: Section of the 1H-NMR of ZFV-3, measured at different temperatures.

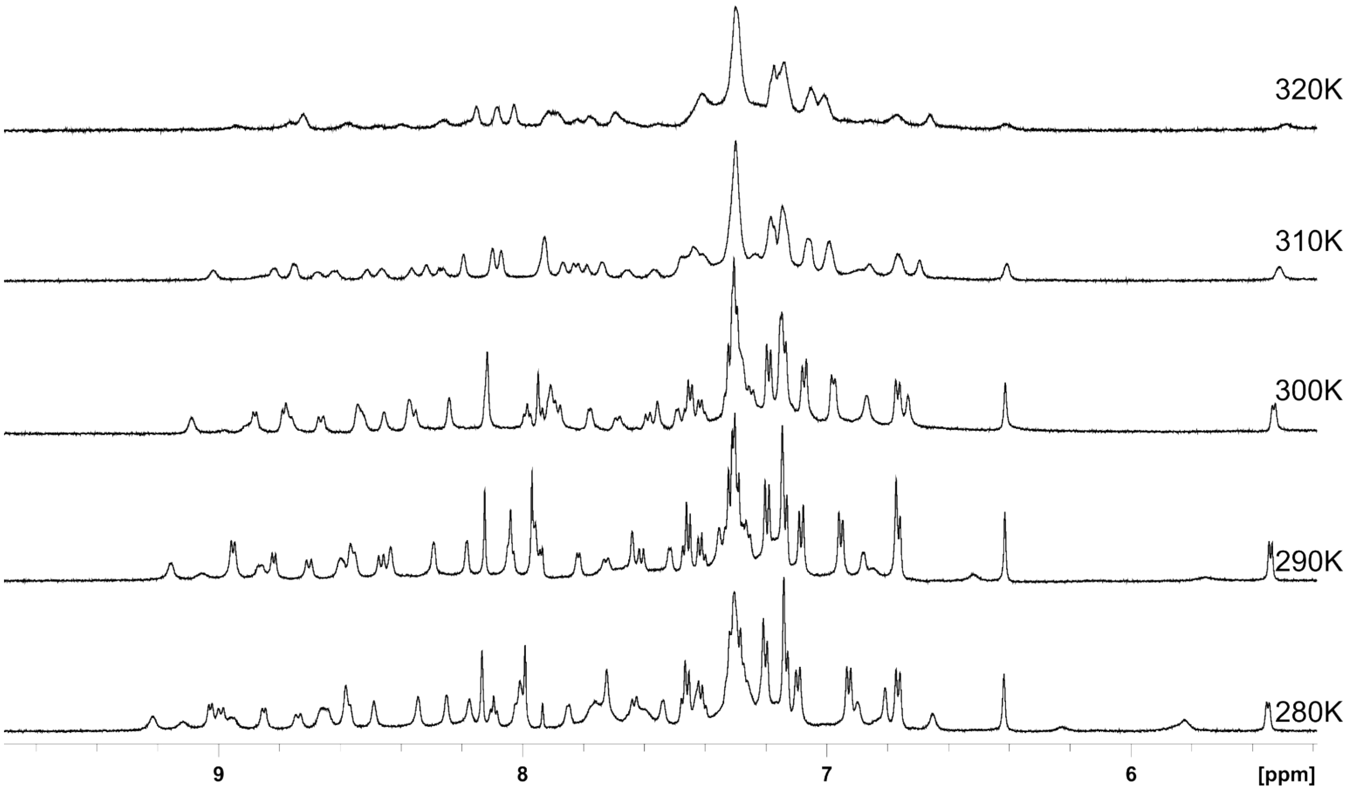

Figure S10: Section of the 1H-NMR of ZFV-4, measured at different temperatures.

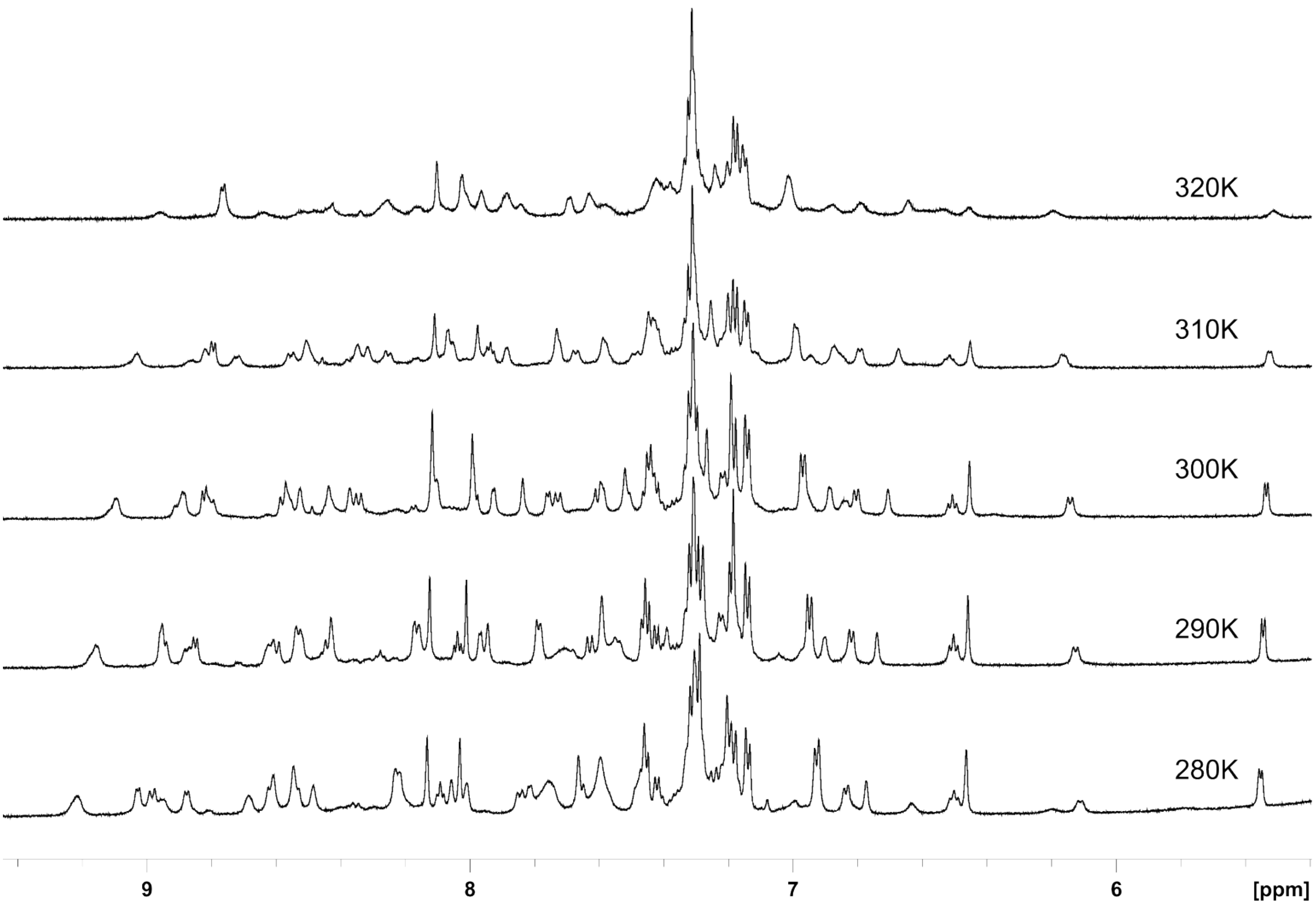

Figure S11: Section of the 1H-NMR of ZFV-5, measured at different temperatures.

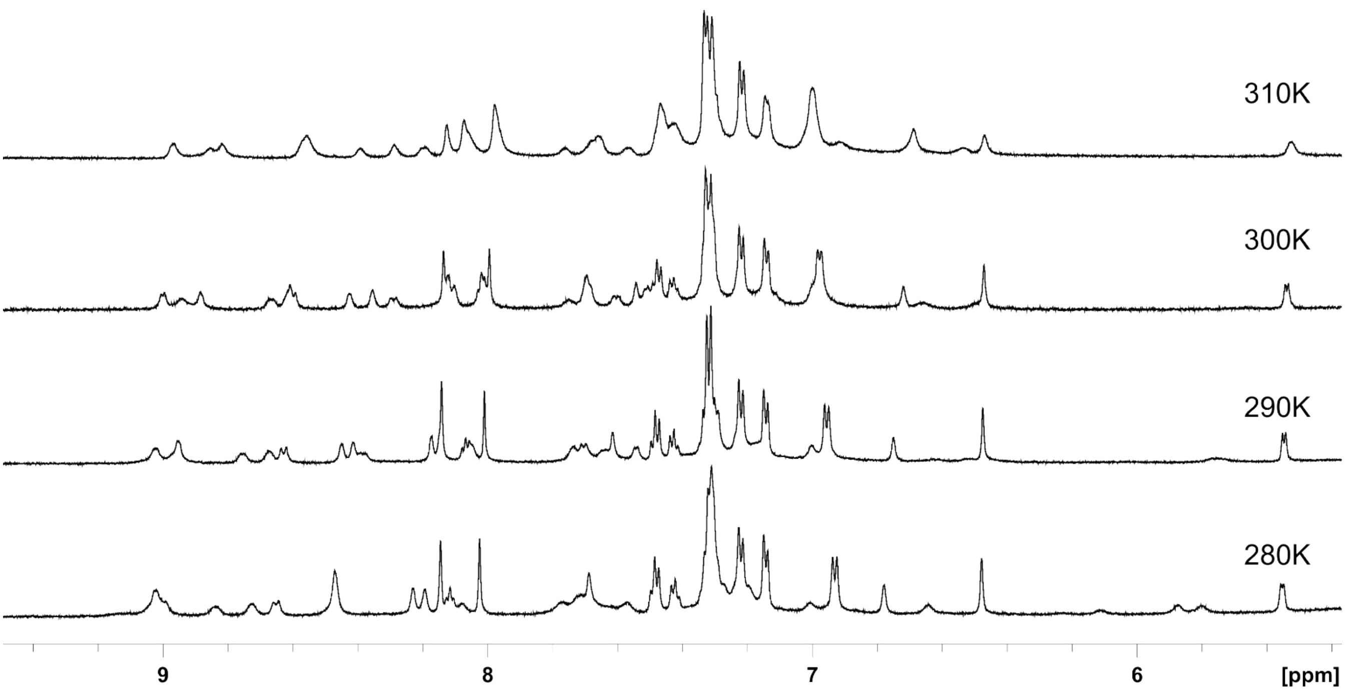

Figure S12: Section of the 1H-NMR of ZFV-6, measured at different temperatures.

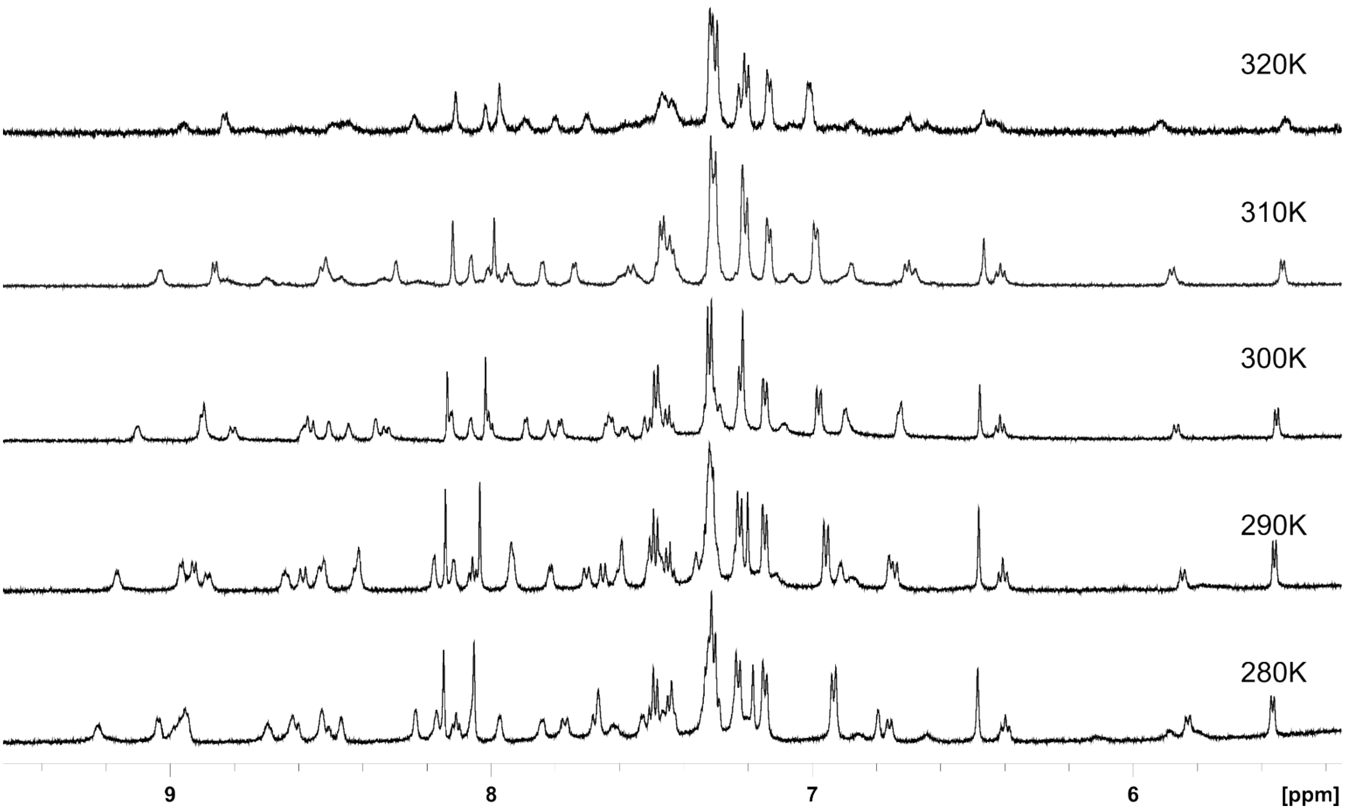

Figure S13: Section of the 1H-NMR of ZFV-7, measured at different temperatures.

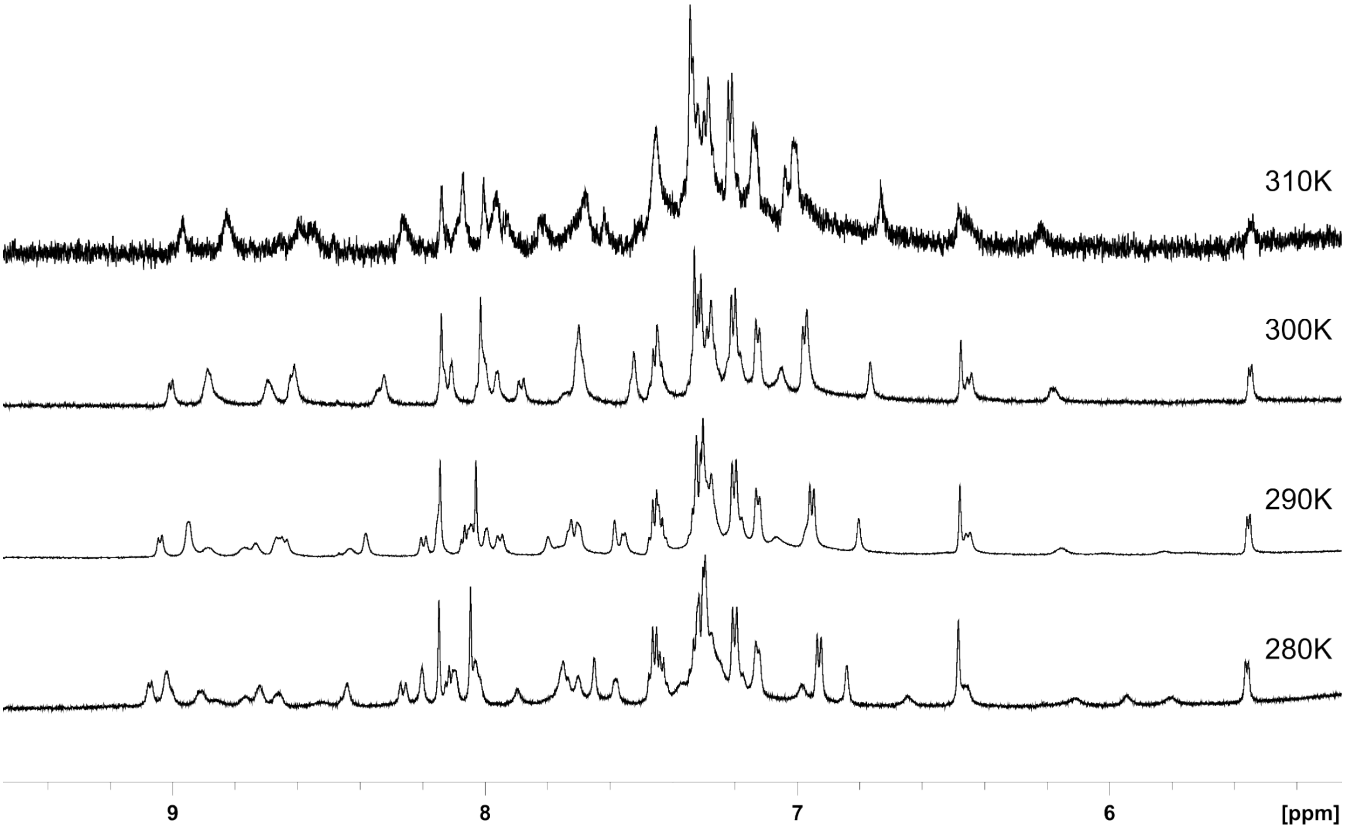

Figure S14: Section of the 1H-NMR of ZFV-8, measured at different temperatures.

ZFV-3 was again subjected to 1H-NMR-measurements at different temperatures. This time, D2O was used as a solvent to reduce signal overlap between aromatic and amide protons.

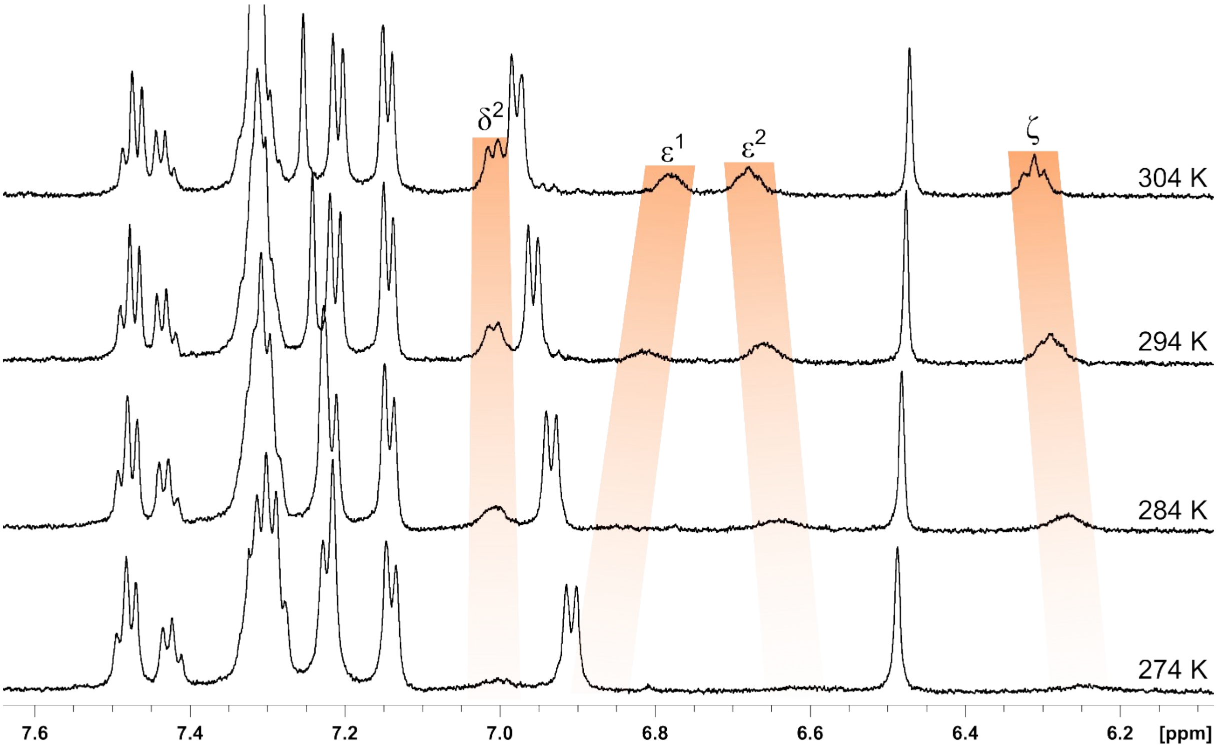

Figure S 15: Section of the 1H-NMR of ZFV-3, measured at different temperatures in D_2O. The aromatic protons of the Fmδ13-building block are highlighted in orange.

Supplementary Computational Results

NMR structure ensembles

The NMR structures were calculated as described by the protocol in the experimental procedures. The following table shows the number of calculated structures and restraints used for each zinc finger variant.

zinc finger variant	number of calculated structures during folding/refinement	number of restraints
3YY1	128/128	191
ZFV-1	256/256	205
ZFV-2	256/256	180
ZFV-3	256/256	158
ZFV-4	128/256	118
ZFV-5	128/256	142
ZFV-7	128/256	115
ZFV-8	256/256	116

For each zinc finger variant, the NMR structure ensemble after the refinement is represented. After the folding procedure, every peptide shows only one conformer regarding the side chain orientation of X13. The exception here is Fmδ13. Out of the 10 lowest energy structures, several conformers have the methyl group inside the hydrophobic core.

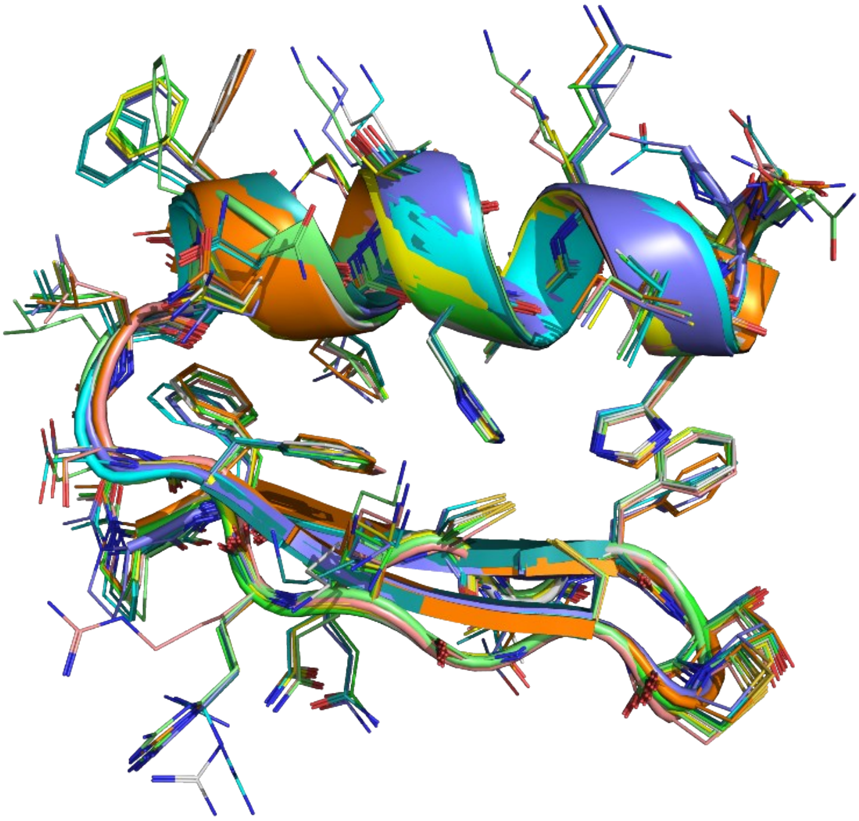

Figure S 16: NMR ensemble of ZFV-1, showing 10 lowest energy structures aligned with the first structure.

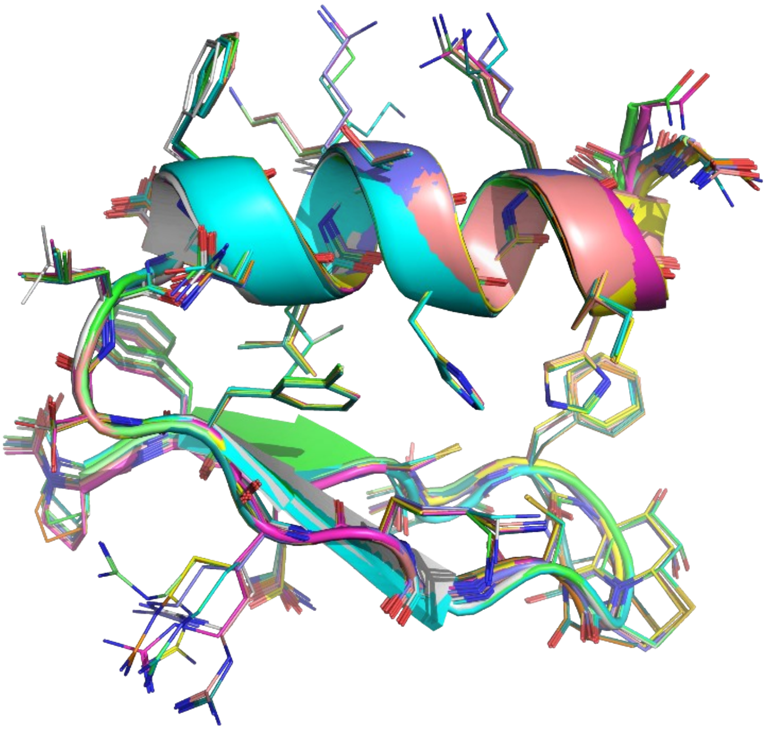

Figure S 17: NMR ensemble of ZFV-2, showing 10 lowest energy structures aligned with the first structure.

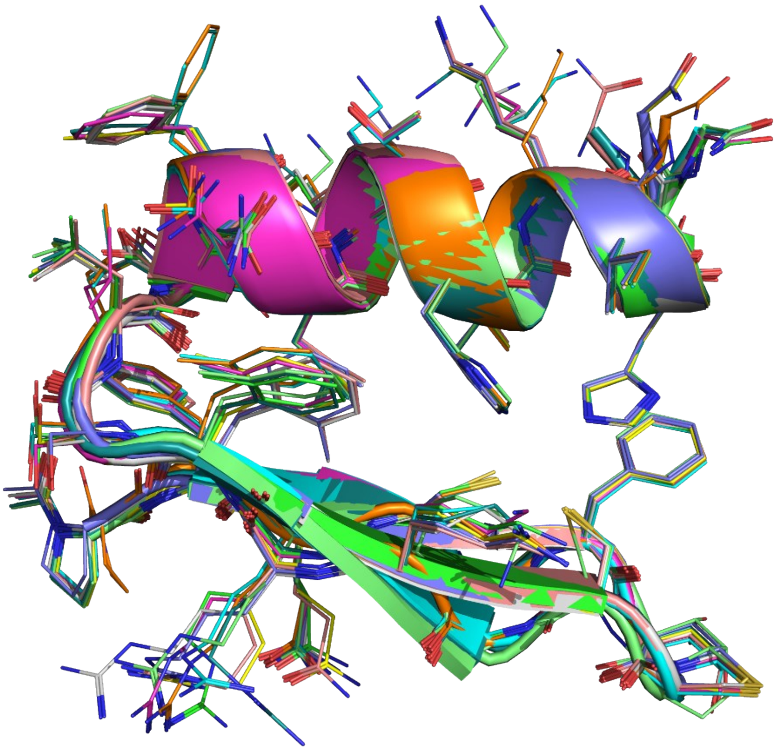

Figure S 18: NMR ensemble of ZFV-3 (folding protocol), showing 10 lowest energy structures aligned with the first structure.

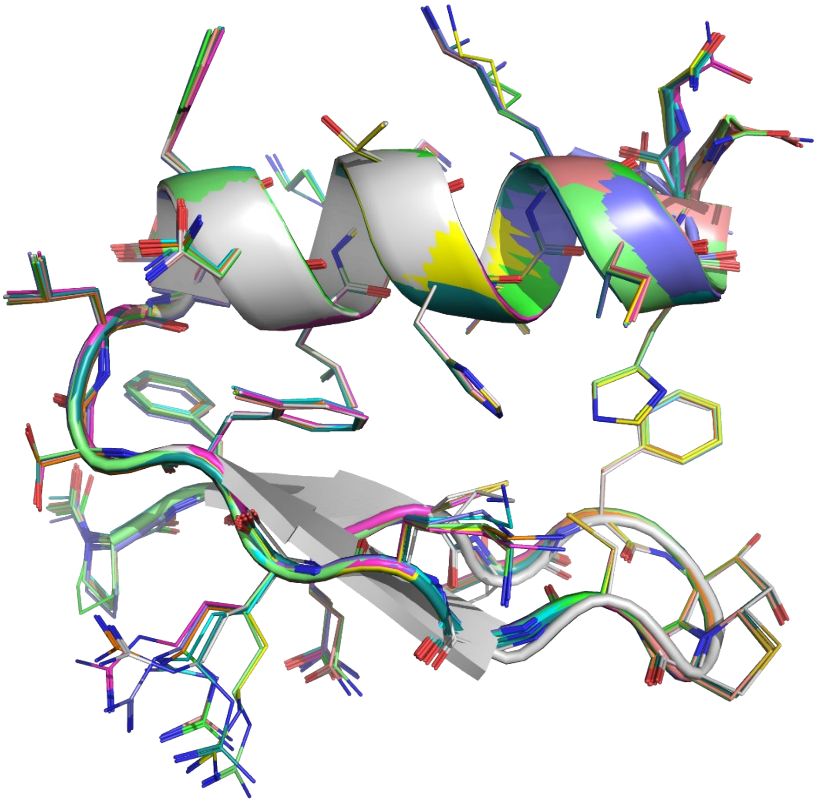

Figure S 19: NMR ensemble of ZFV-3 (refinement protocol), showing 10 lowest energy structures aligned with the first structure.

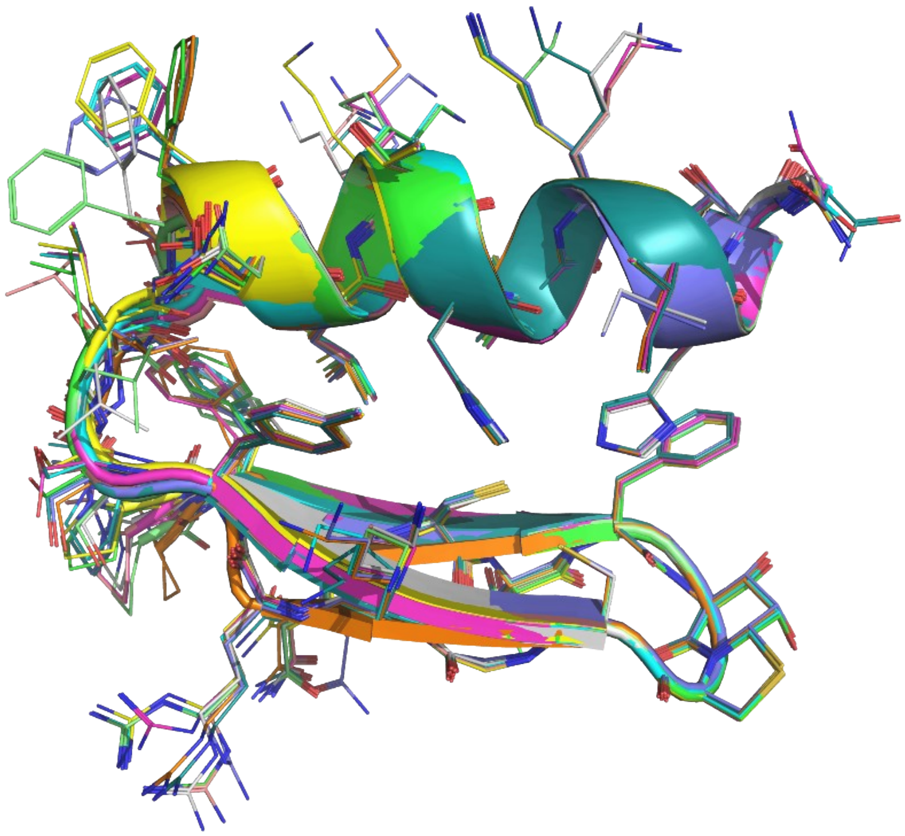

Figure S 20: NMR ensemble of ZFV-4, showing 10 lowest energy structures aligned with the first structure.

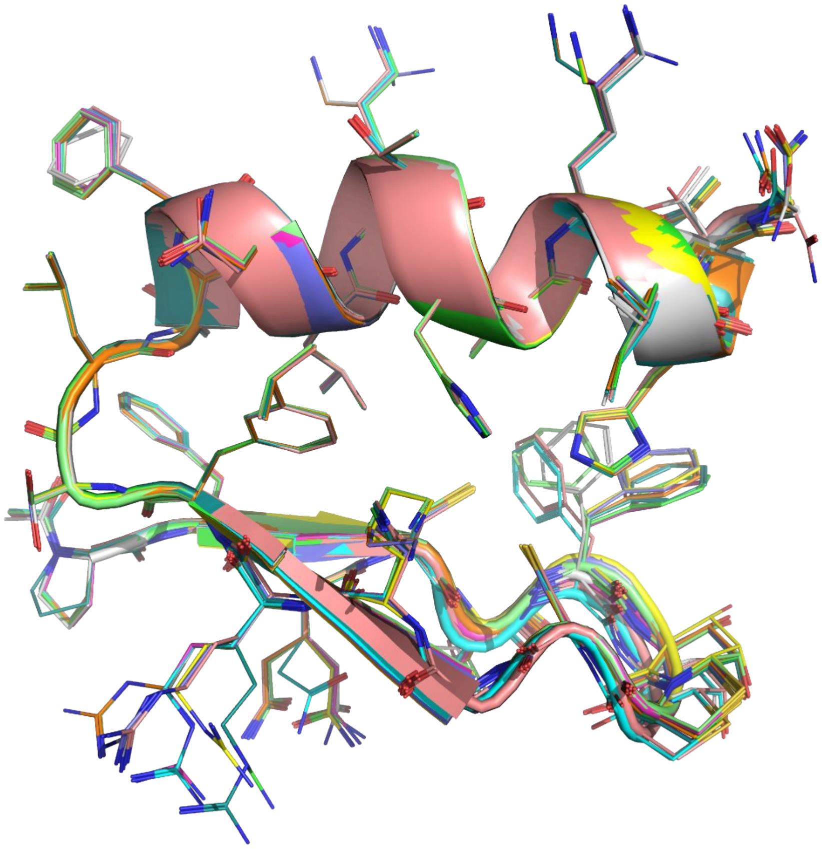

Figure S 21: NMR ensemble of ZFV-5, showing 10 lowest energy structures aligned with the first structure.

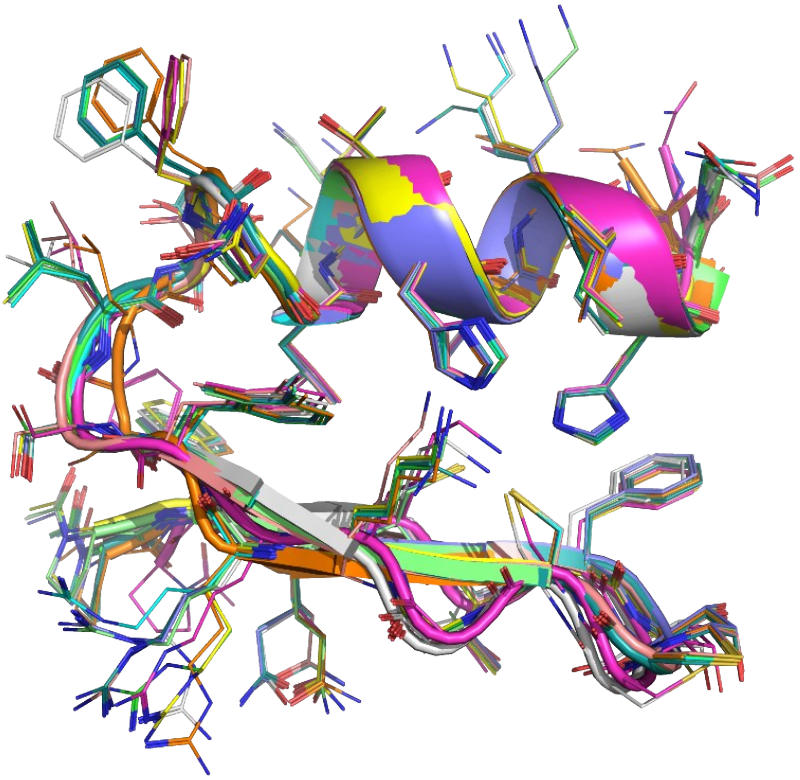

Figure S 22: NMR ensemble of ZFV-7, showing 10 lowest energy structures aligned with the first structure.

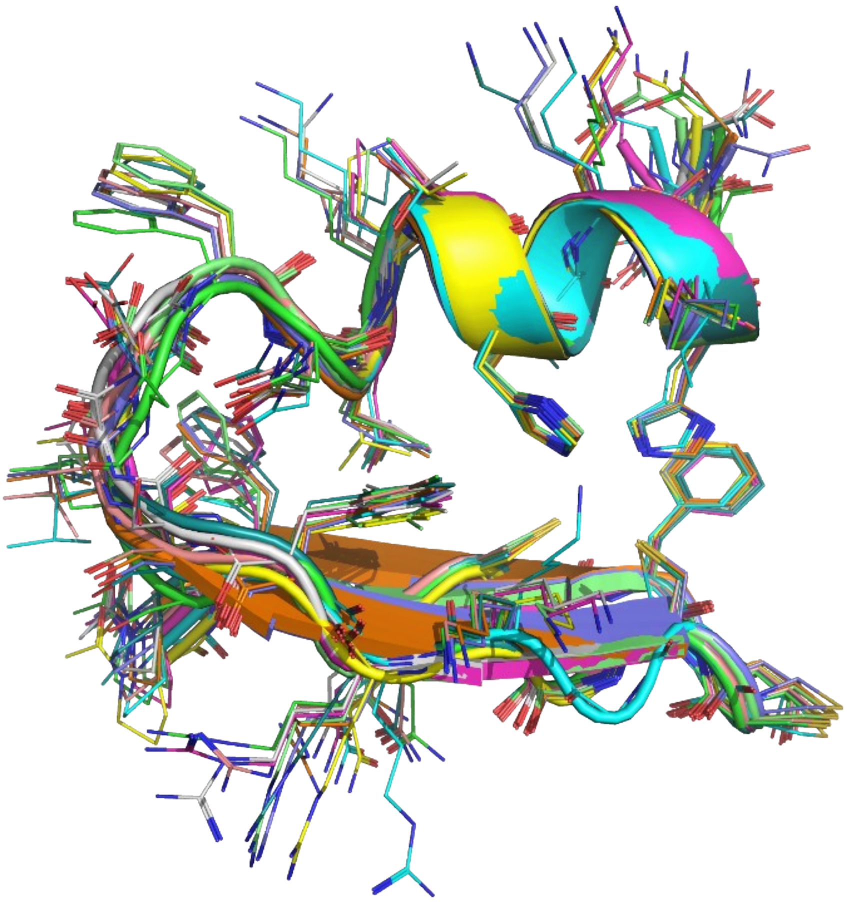

Figure S 23: NMR ensemble of ZFV-8, showing 10 lowest energy structures aligned with the first structure.

Convergence of well-tempered-Metadynamics.

One important criterion for wt-metadynamics is the convergence. One good estimation is the height of the Gaussian deployed during the simulation. As observed in Fig S14, the height of the Gaussian steadily decreases. Only small energy values were deployed after the initial huge spike. Furthermore, crossing events should still be observed, even though no Gaussian is deposited. The next criterion is the overall shape of the FES. If a wt-metadynamics has converged, no major changes apart from shifts of the minima should be detected. For every Zinc finger peptide 3YY1, ZFV-1, ZFV-2, ZFV-3, ZFV-5 and ZFV-7 those criteria were fulfilled. Only ZFV-8 showed an additional deposition of a potential just before 1500 ns, which prompted us to extend the simulation to 2000 ns. No further change of the FES from 1500-2000 ns could be observed.

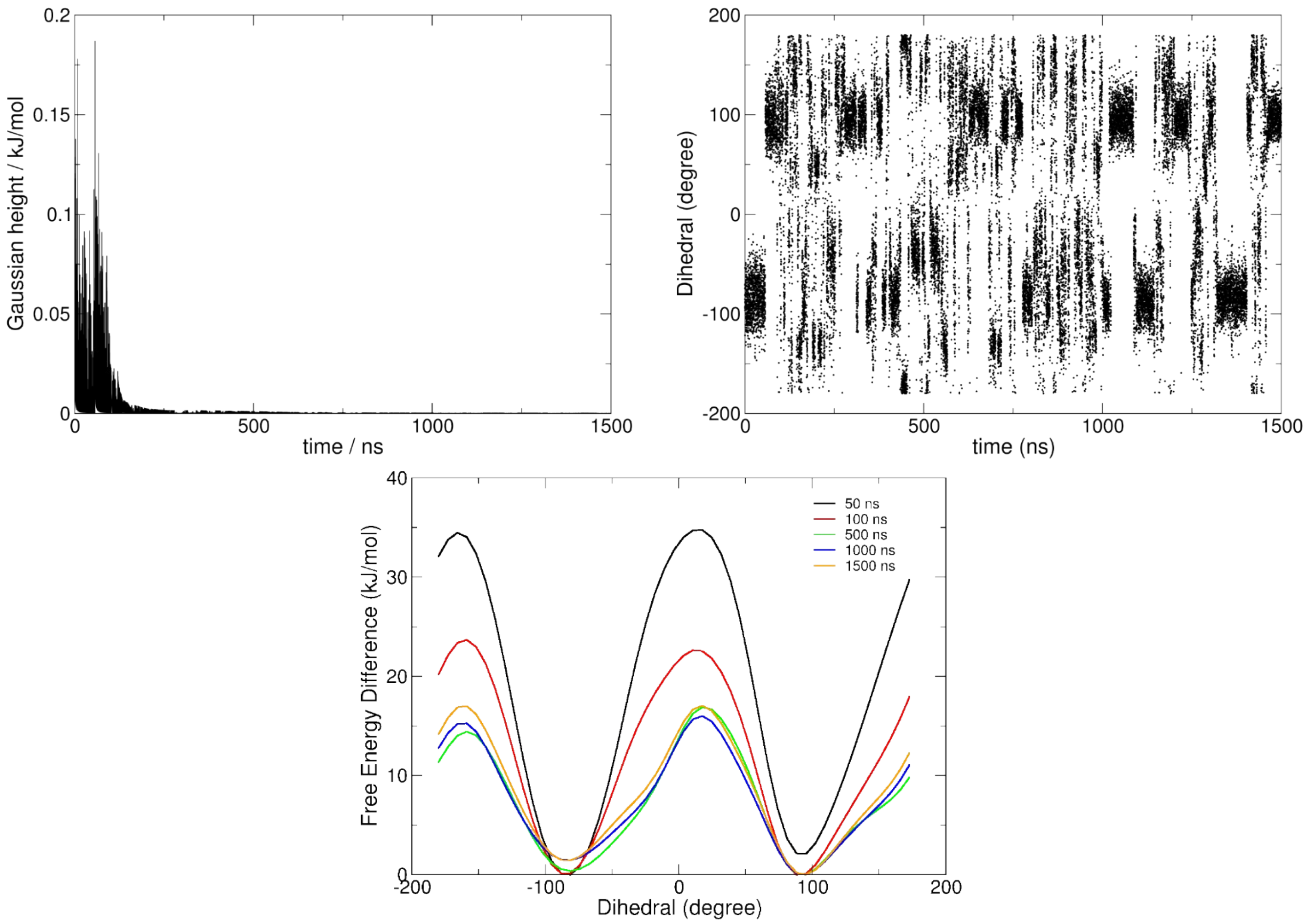

Figure S24: Free energy convergence estimation for 3YY1. Top row left: Height of the Gaussian potential deposited over the course of the wt-Metadynamics. Top row right: Evolution of the dihedral of Phe^{13} during wt-Metadynamics. Bottom row: Development of the free energy profile obtained directly from wt-Metadynamics during different time periods.

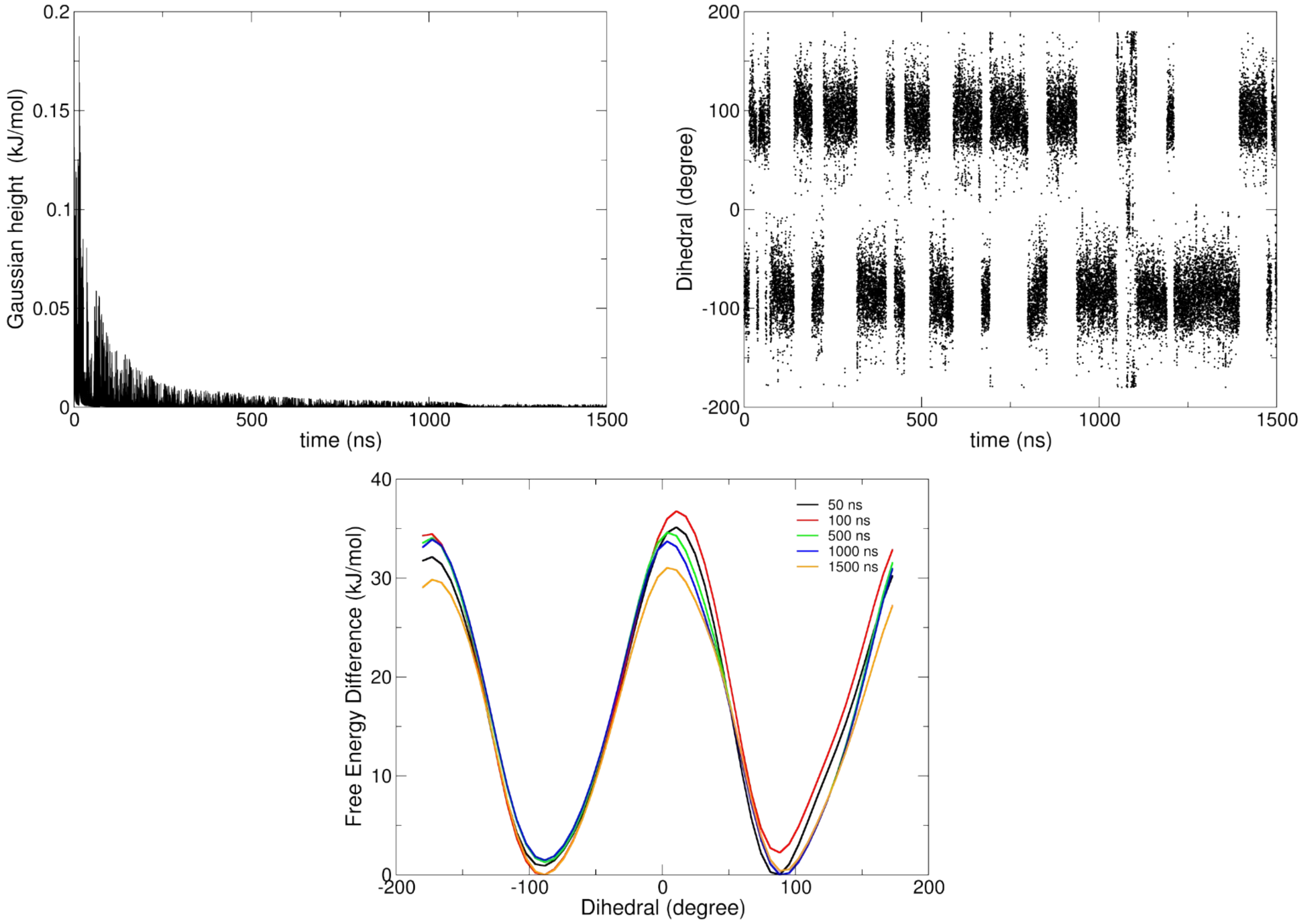

Figure S25: Free energy convergence estimation for ZFV-1. Top row left: Height of the Gaussian potential deposited over the course of the wt-Metadynamics. Top row right: Evolution of the dihedral of Phe[13] during wt-Metadynamics. Bottom row: Development of the free energy profile obtained directly from wt-Metadynamics during different time periods.

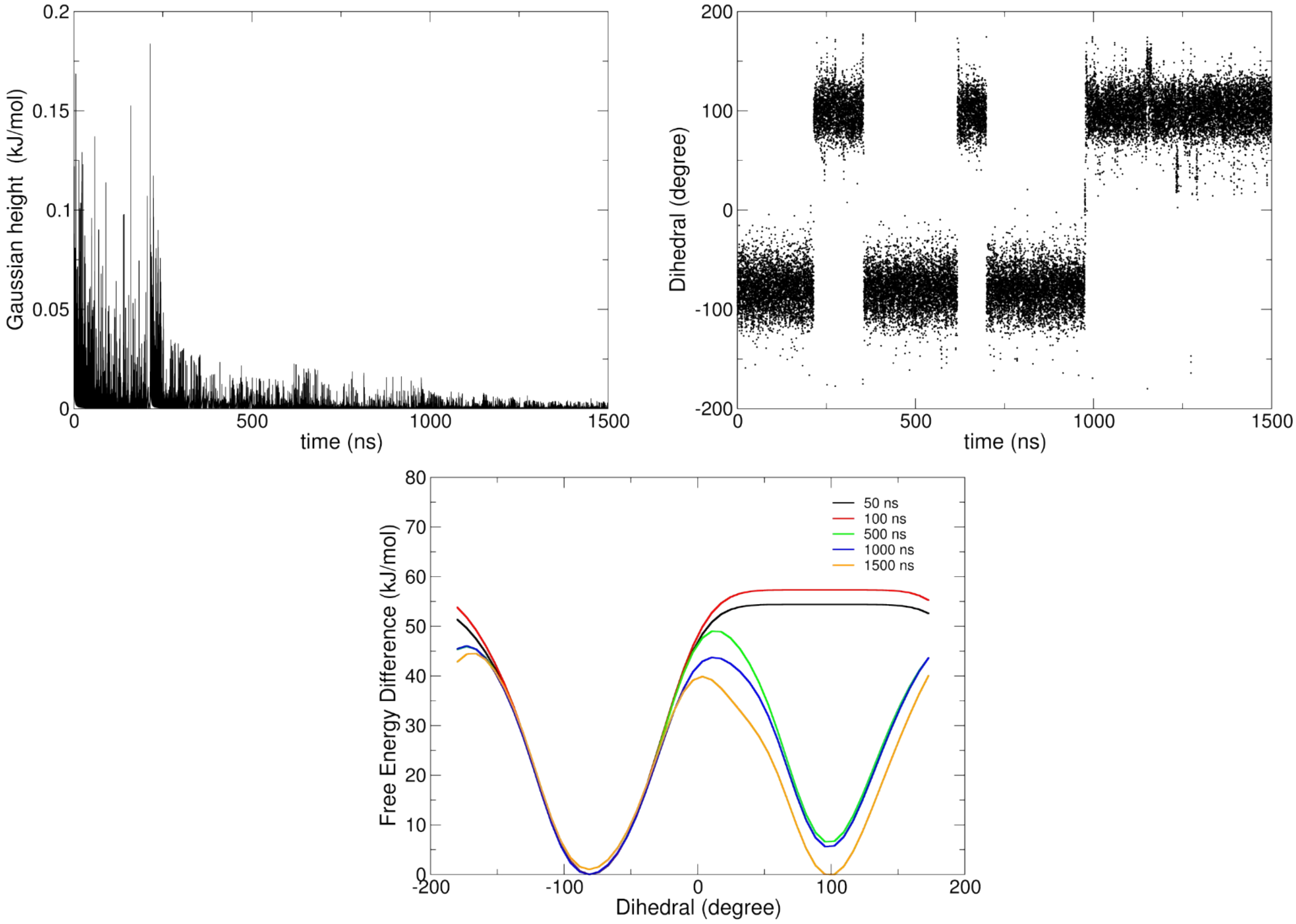

Figure S26: Free energy convergence estimation for ZFV-2. Top row left: Height of the Gaussian potential deposited over the course of the wt-Metadynamics. Top row right: Evolution of the dihedral of Phe[13] during wt-Metadynamics. Bottom row: Development of the free energy profile obtained directly from wt-Metadynamics during different time periods.

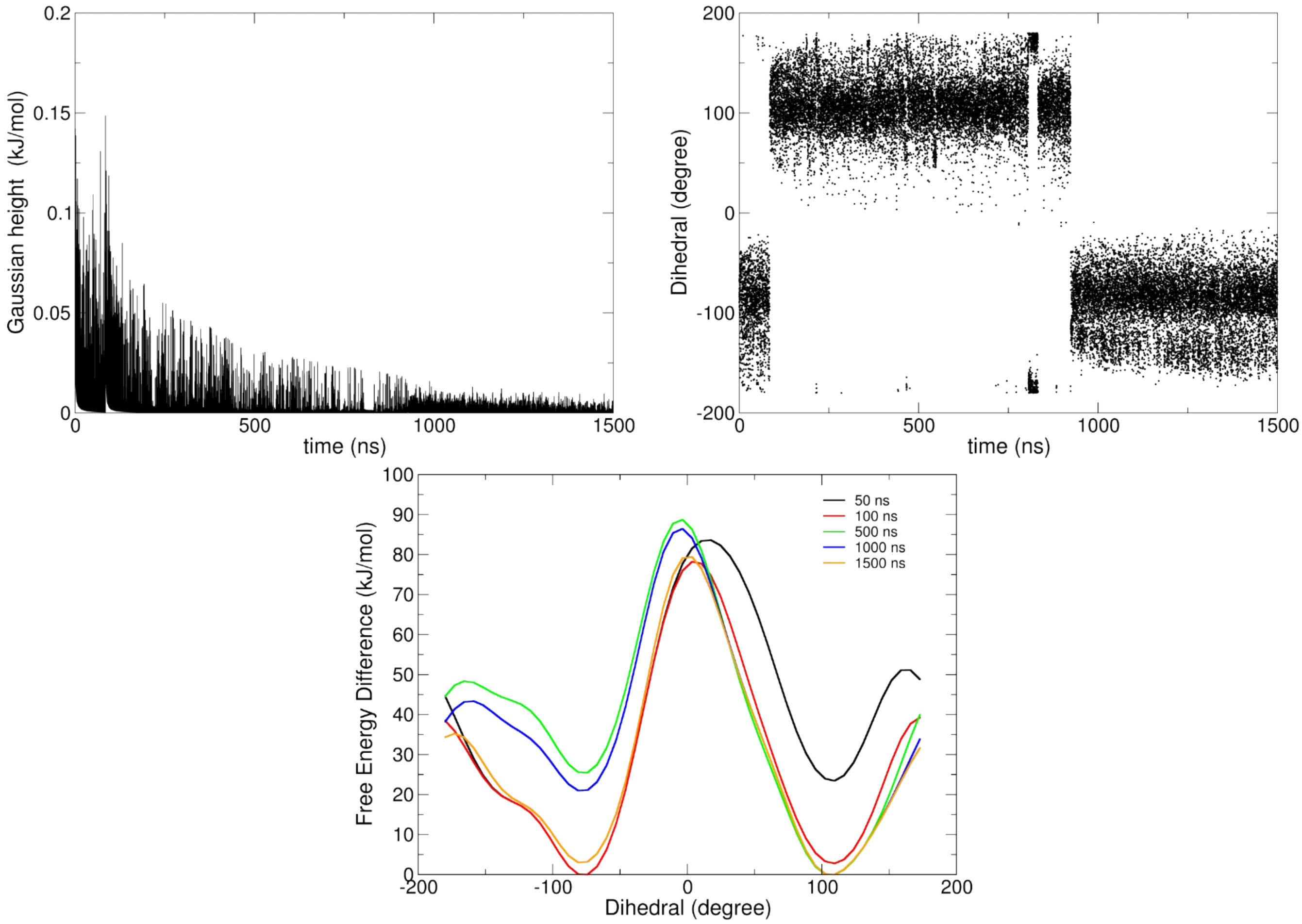

Figure S27: Free energy convergence estimation for ZFV-3. Top row left: Height of the Gaussian potential deposited over the course of the wt-Metadynamics. Top row right: Evolution of the dihedral of Phe[13] during wt-Metadynamics. Bottom row: Development of the free energy profile obtained directly from wt-Metadynamics during different time periods.

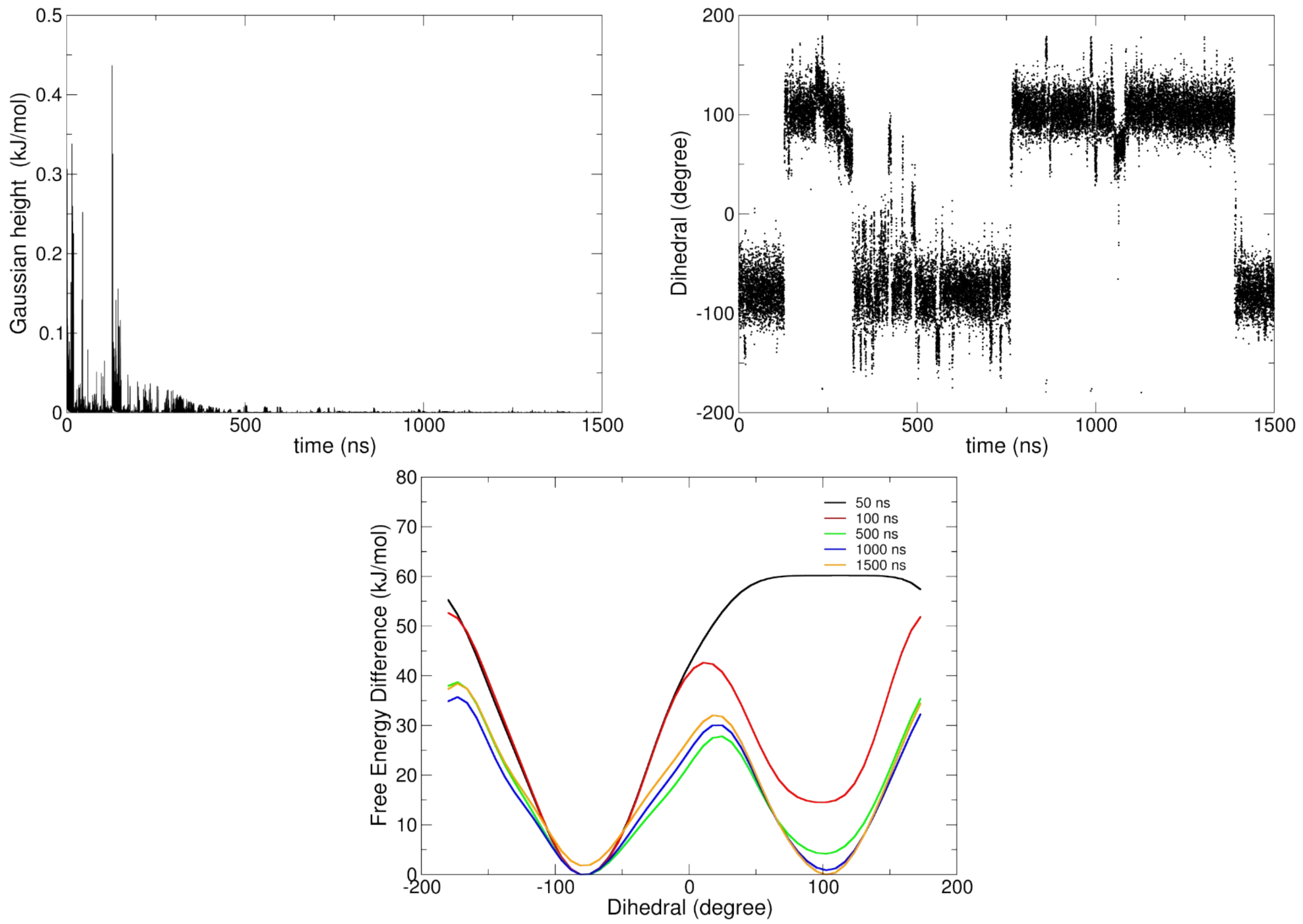

Figure S28: Free energy convergence estimation for ZFV-5. Top row left: Height of the Gaussian potential deposited over the course of the wt-Metadynamics. Top row right: Evolution of the dihedral of Phe[13] during wt-Metadynamics. Bottom row: Development of the free energy profile obtained directly from wt-Metadynamics during different time periods.

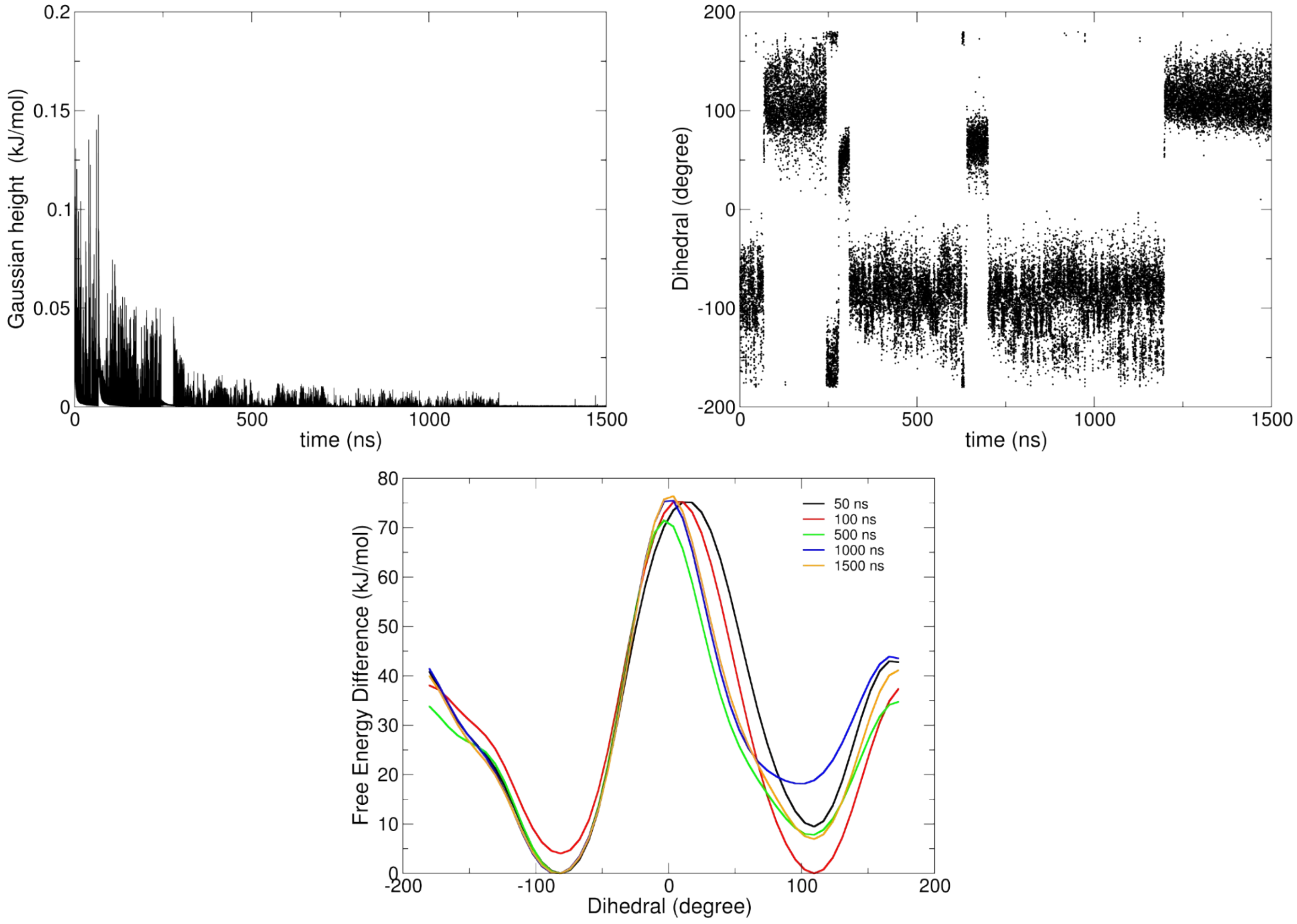

Figure S29: Free energy convergence estimation for ZFV-7. Top row left: Height of the Gaussian potential deposited over the course of the wt-Metadynamics. Top row right: Evolution of the dihedral of Phe[13] during wt-Metadynamics. Bottom row: Development of the free energy profile obtained directly from wt-Metadynamics during different time periods.

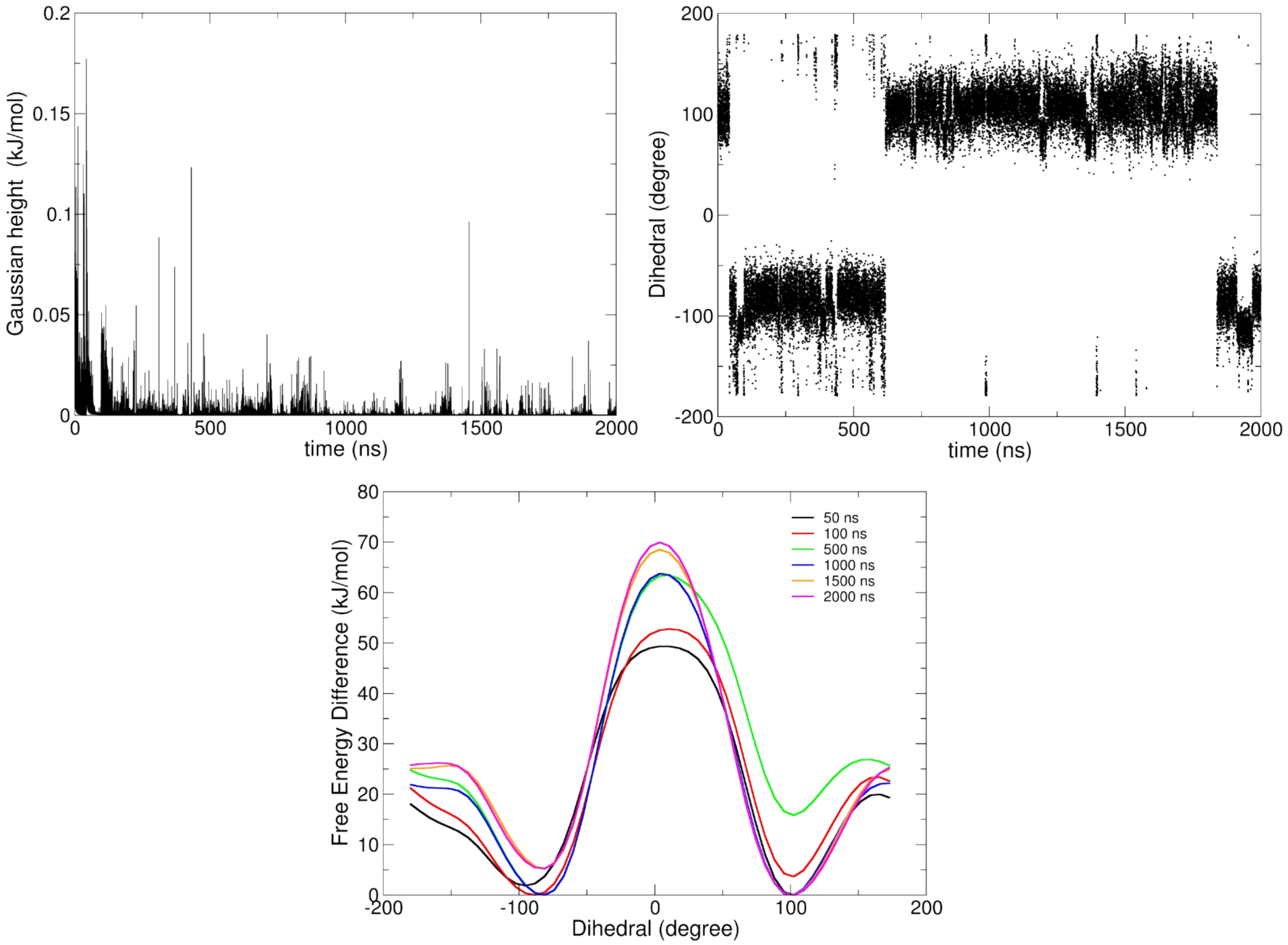

Figure S30: Free energy convergence estimation for ZFV-8. Top row left: Height of the Gaussian potential deposited over the course of the wt-Metadynamics. Top row right: Evolution of the dihedral of Phe[13] during wt-Metadynamics. Bottom row: Development of the free energy profile obtained directly from wt-Metadynamics during different time periods.

Supplementary spectroscopic Data for unnatural building blocks

1H-NMR of S3

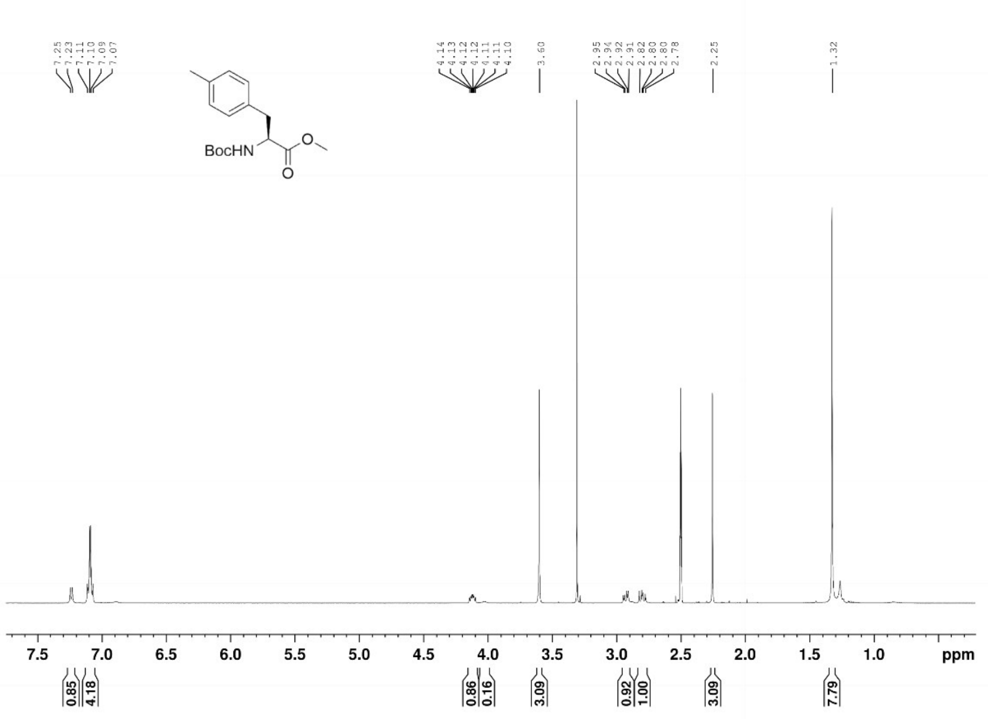

13C-NMR of S3

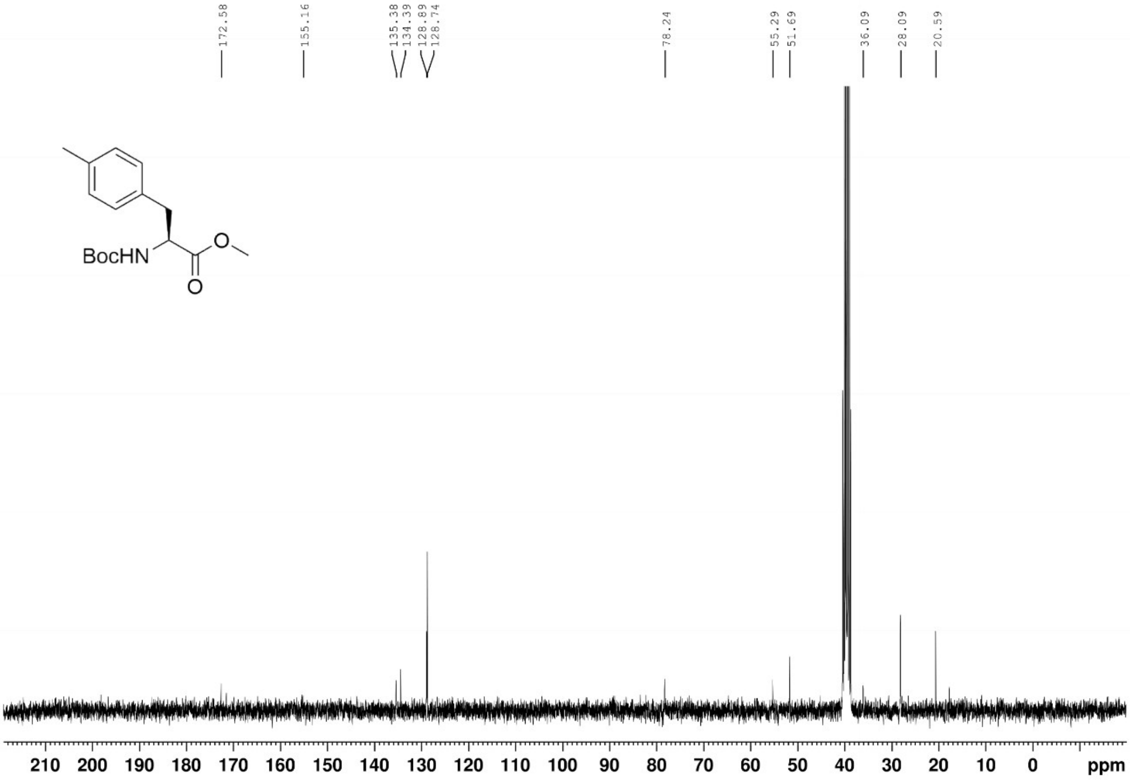

1H-NMR of S11

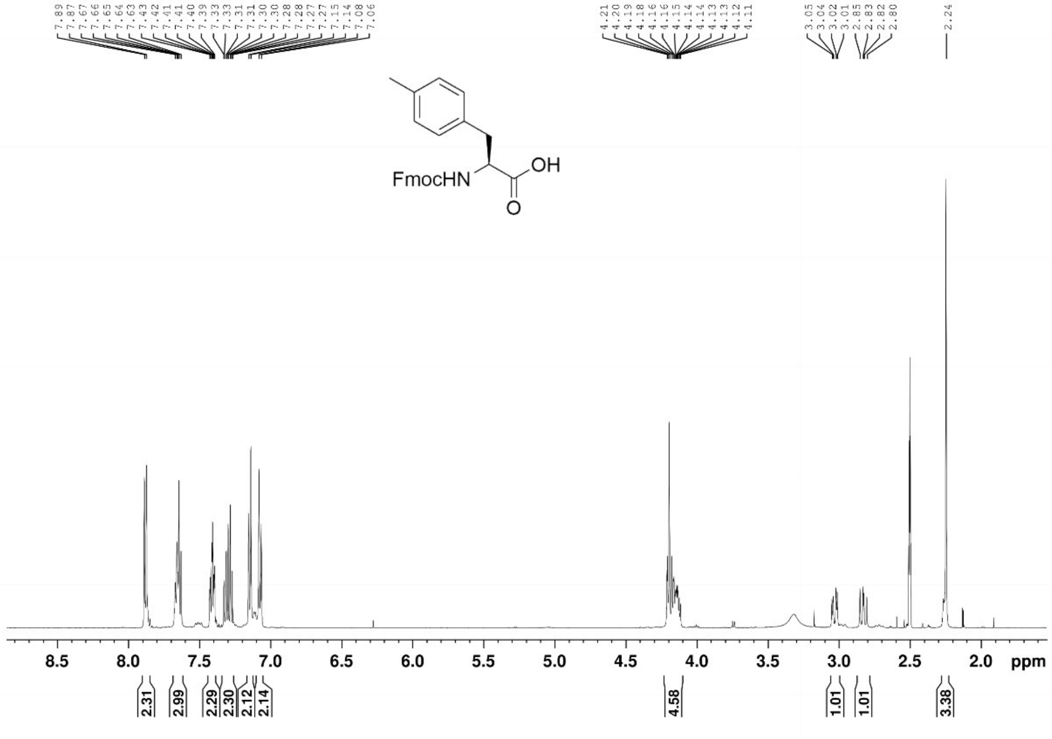

13C-NMR of S11

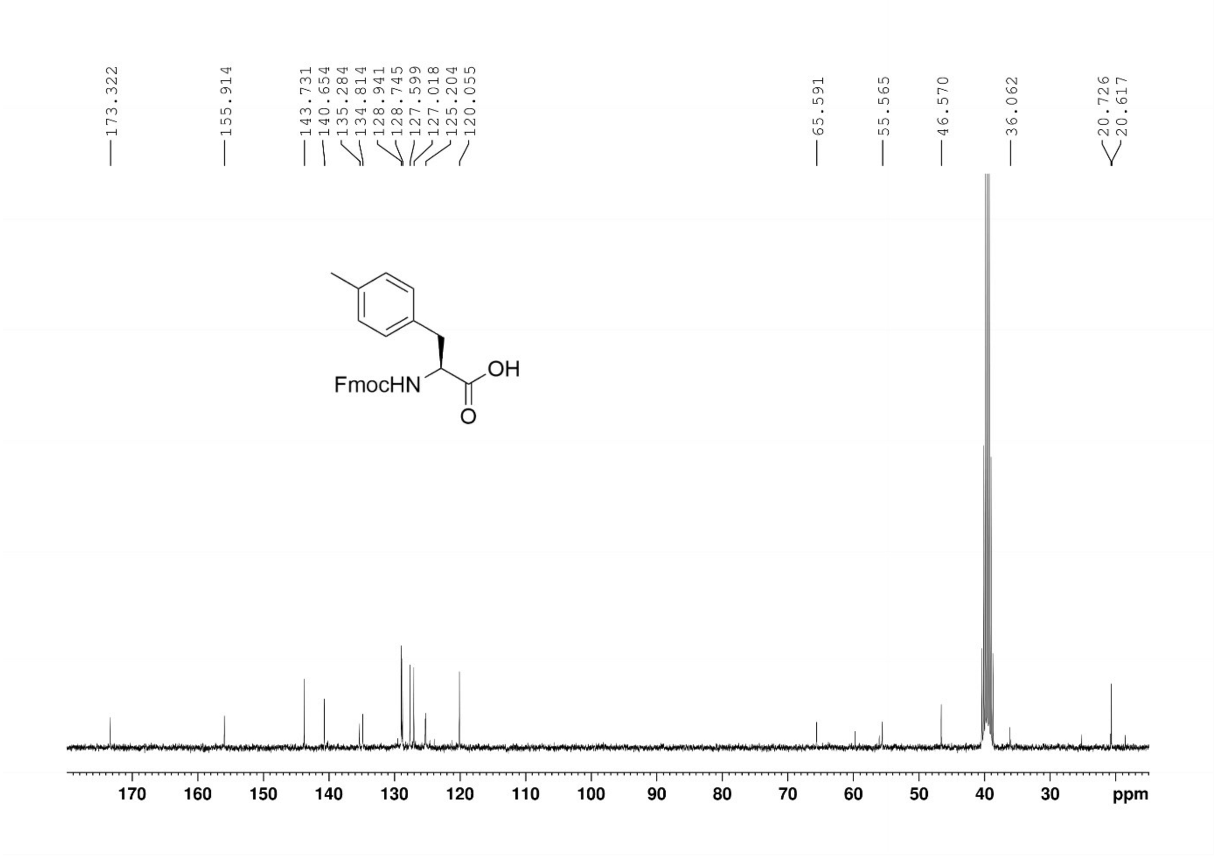
173.322
155.914
143.731
140.654
135.284
134.814
128.941
128.745
127.599
127.018
125.204
120.055
65.591
55.565
46.570
36.062
20.726
20.617
OH
FmocHN
O
170 160 150 140 130 120 110 100 90 80 70 60 50 40 30 ppm

1H-NMR of S5

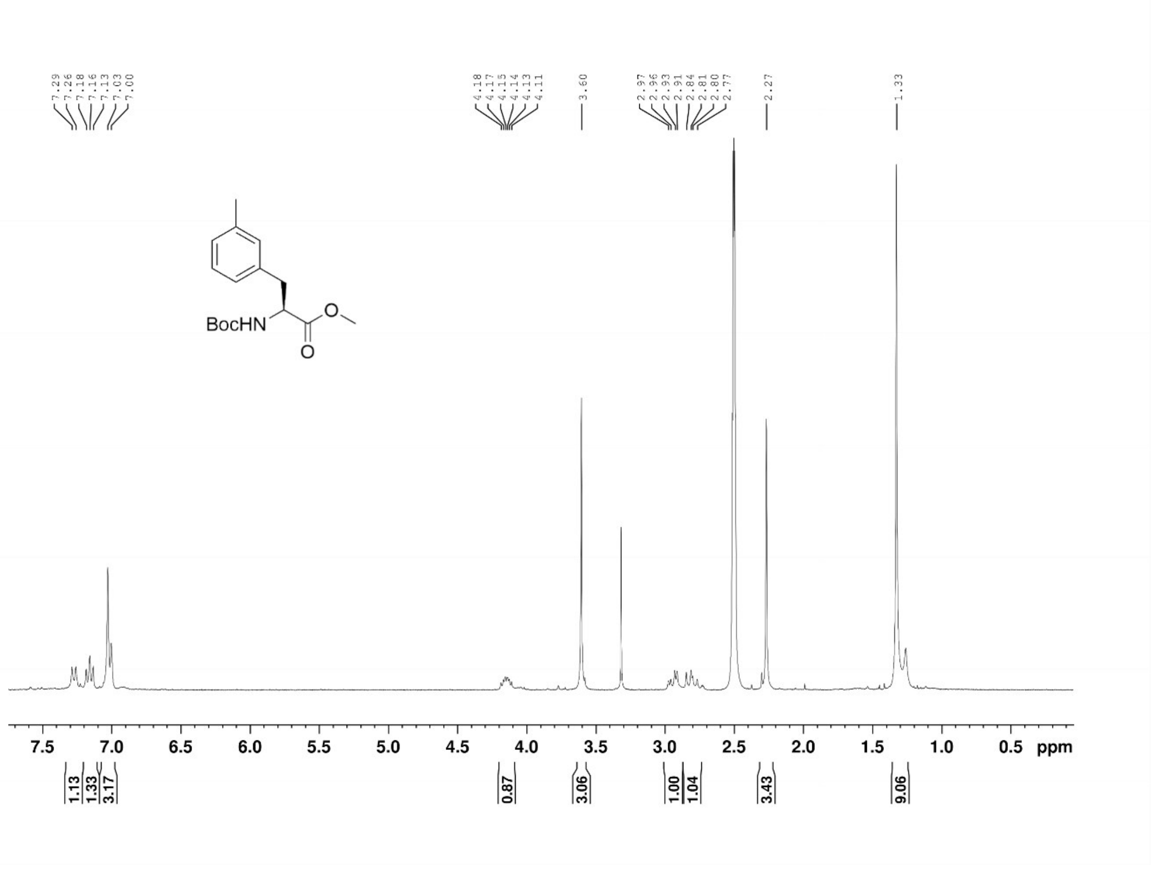
7.29
7.26
7.18
7.16
7.13
7.03
7.00
4.18
4.17
4.15
4.14
4.13
4.11
3.60
2.97
2.96
2.93
2.91
2.84
2.81
2.80
2.77
2.27
1.33
BocHN
O
O
7.5 7.0 6.5 6.0 5.5 5.0 4.5 4.0 3.5 3.0 2.5 2.0 1.5 1.0 0.5 ppm
1.13
1.33
3.17
0.87
3.06
1.00
1.04
3.43
9.06

13C-NMR of S5

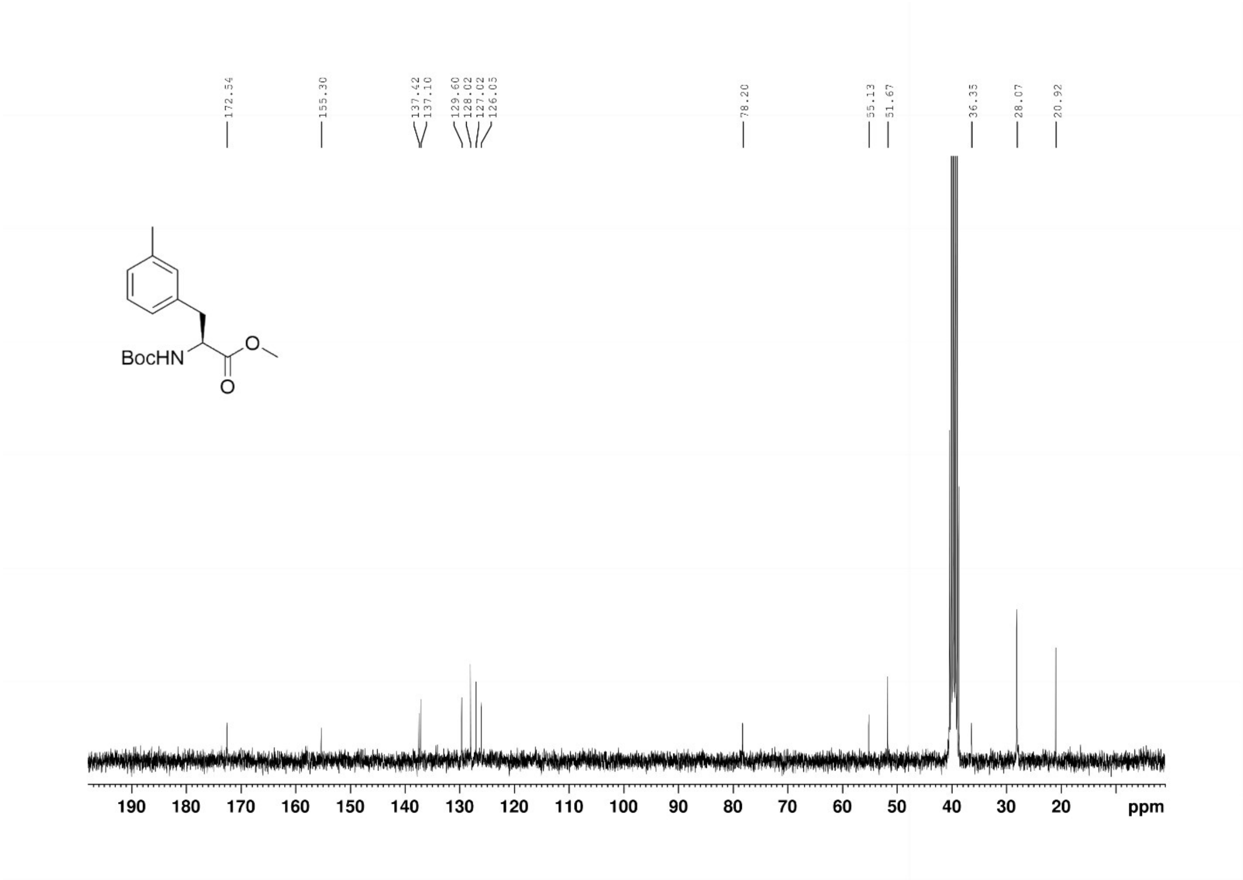

1H-NMR of S6

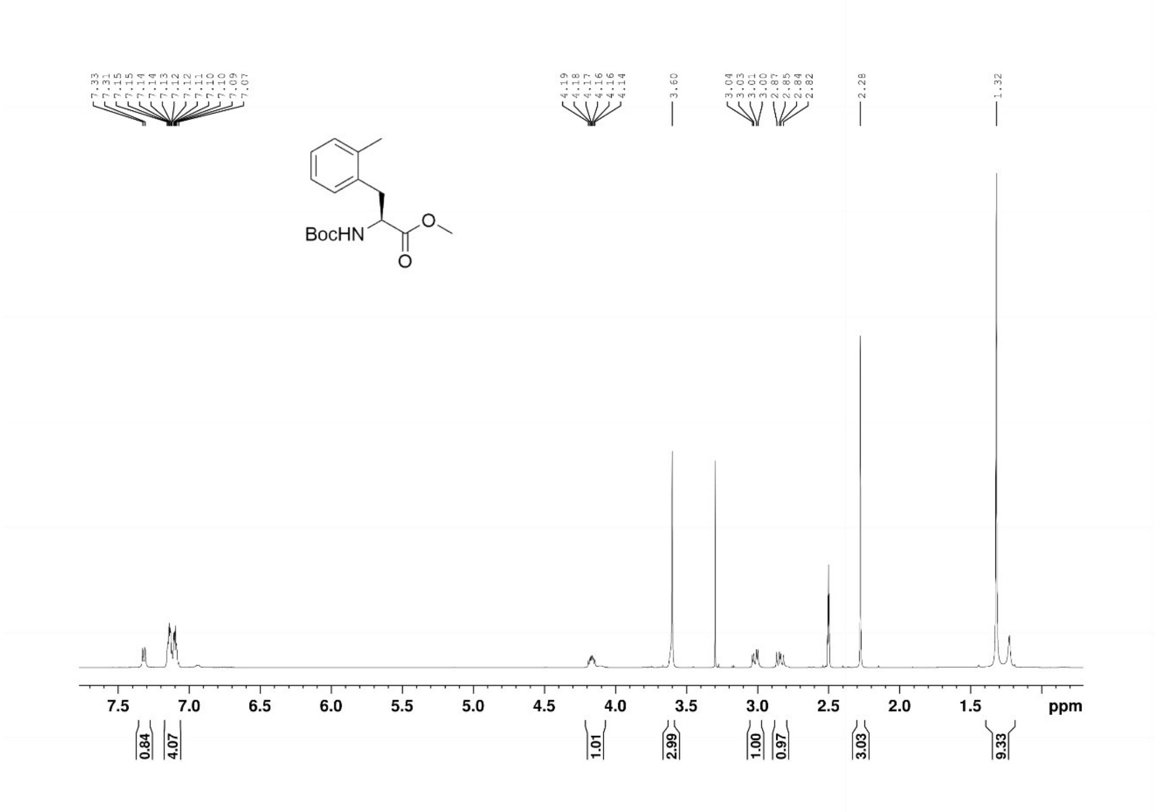

13C-NMR of S6

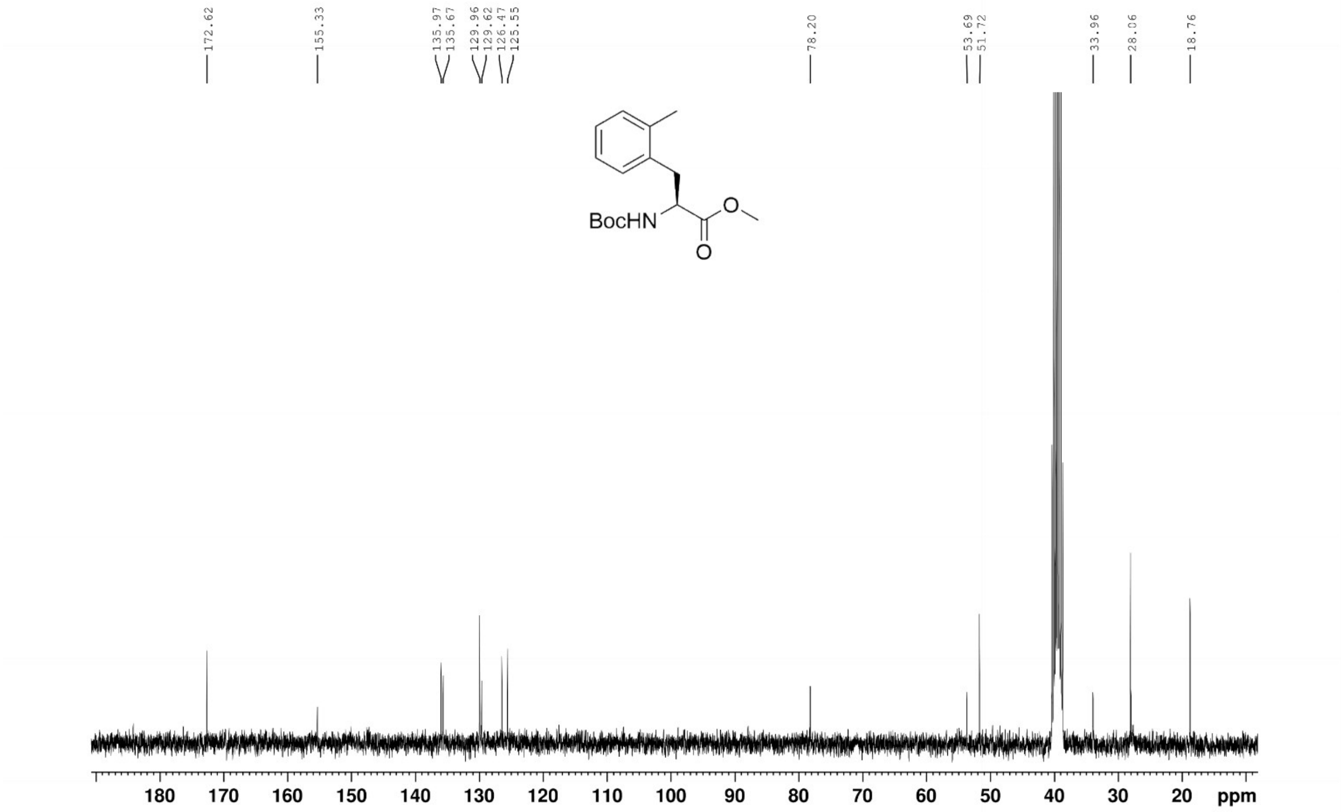

1H-NMR of S7

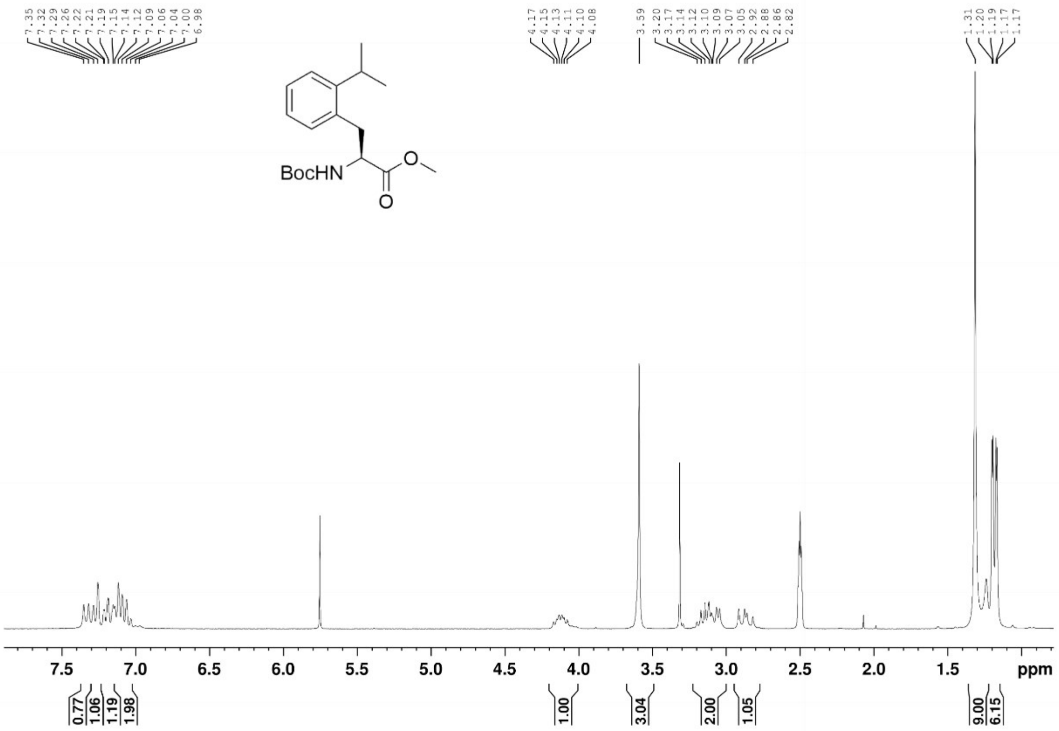

13C-NMR of S7

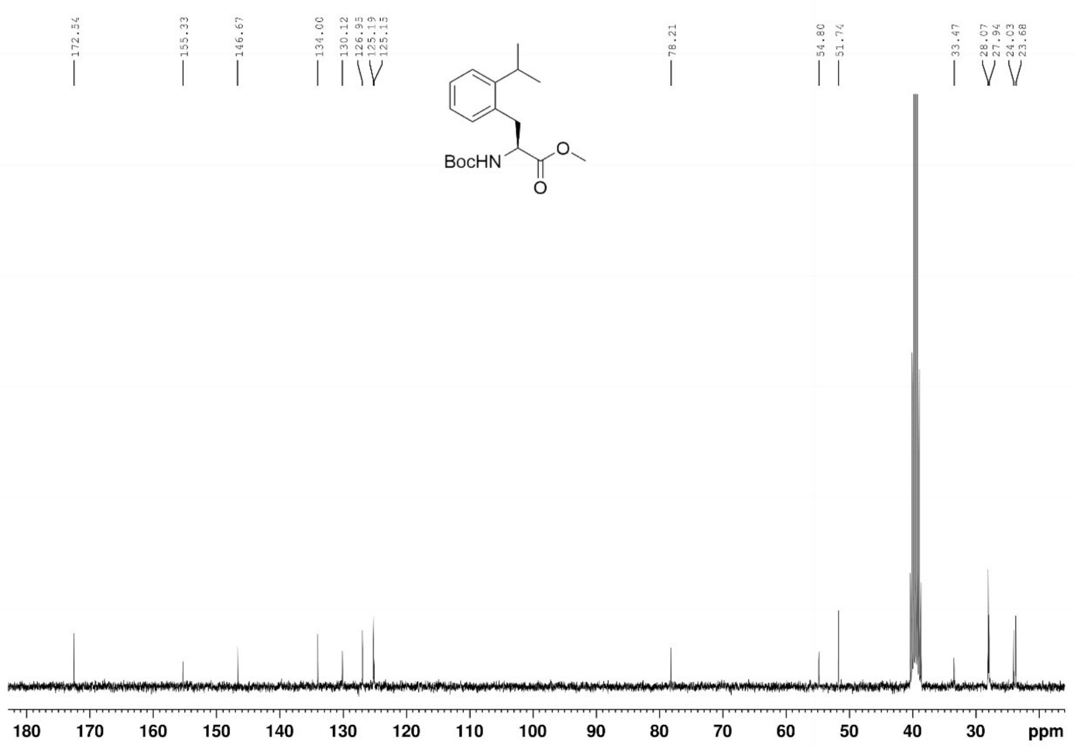

1H-NMR of S8

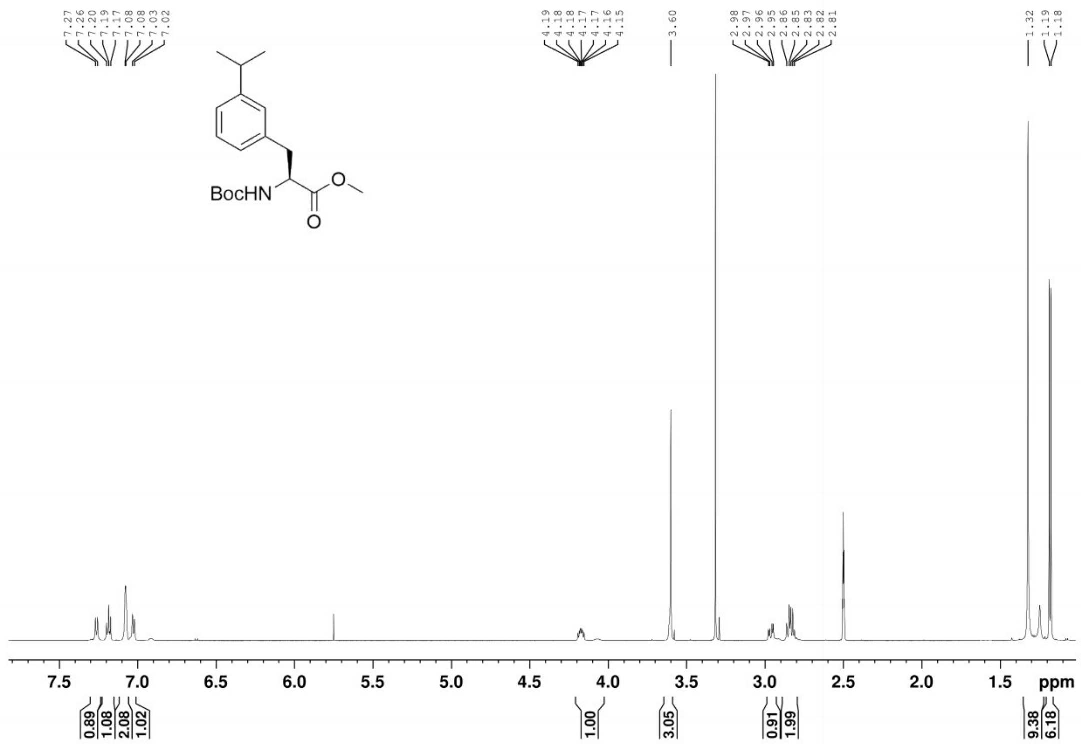

13C-NMR of S8

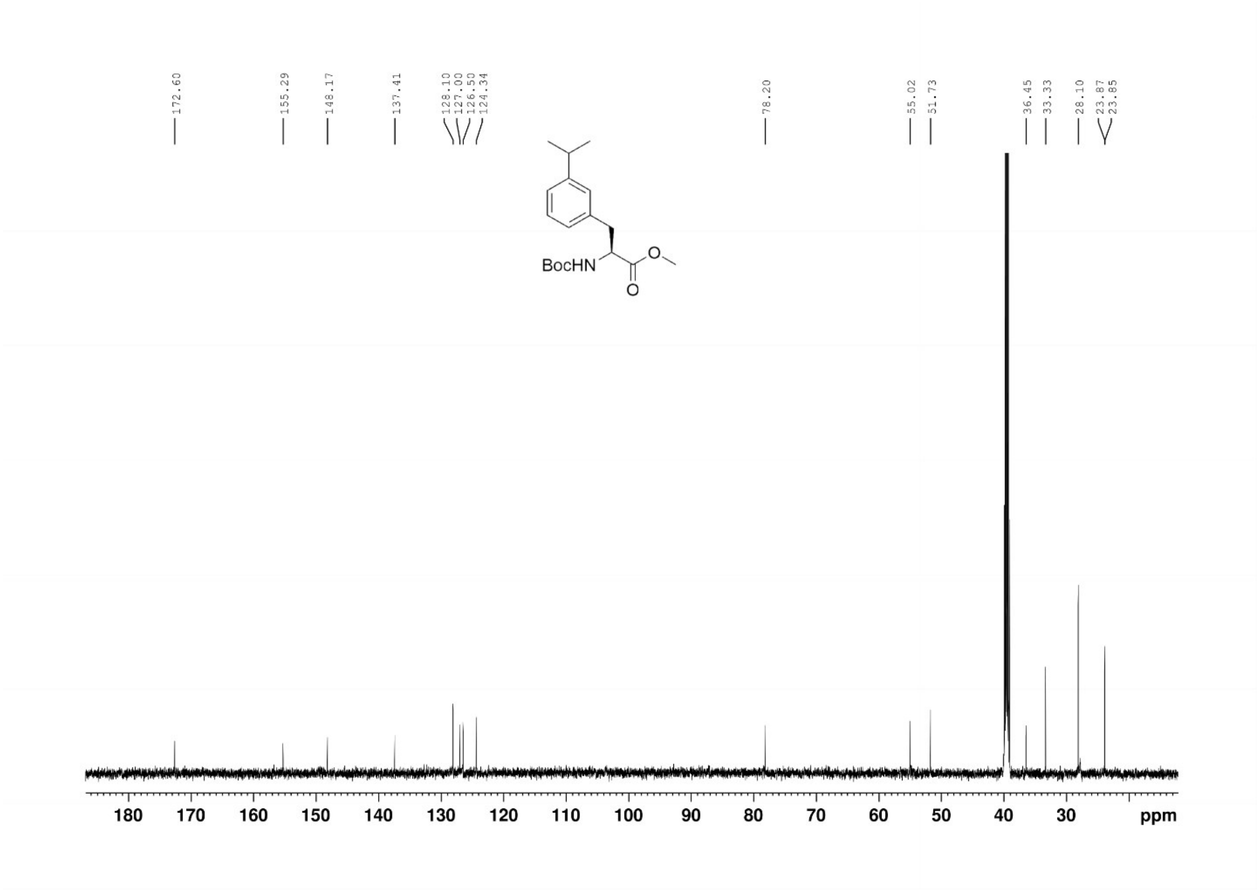

1H-NMR of S9

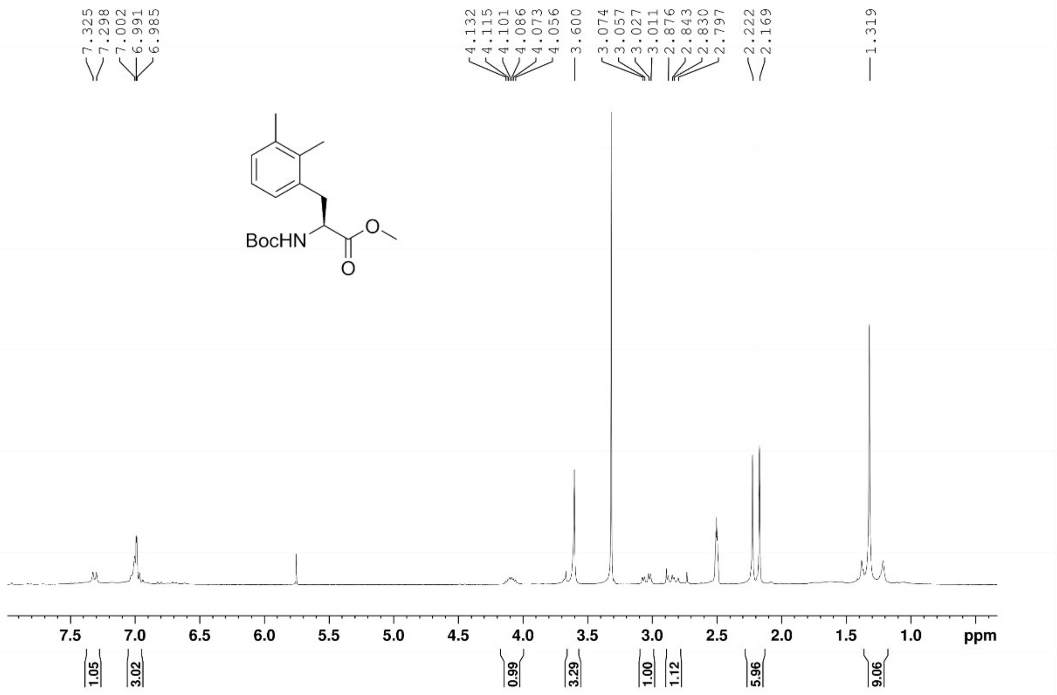

13C-NMR of S9

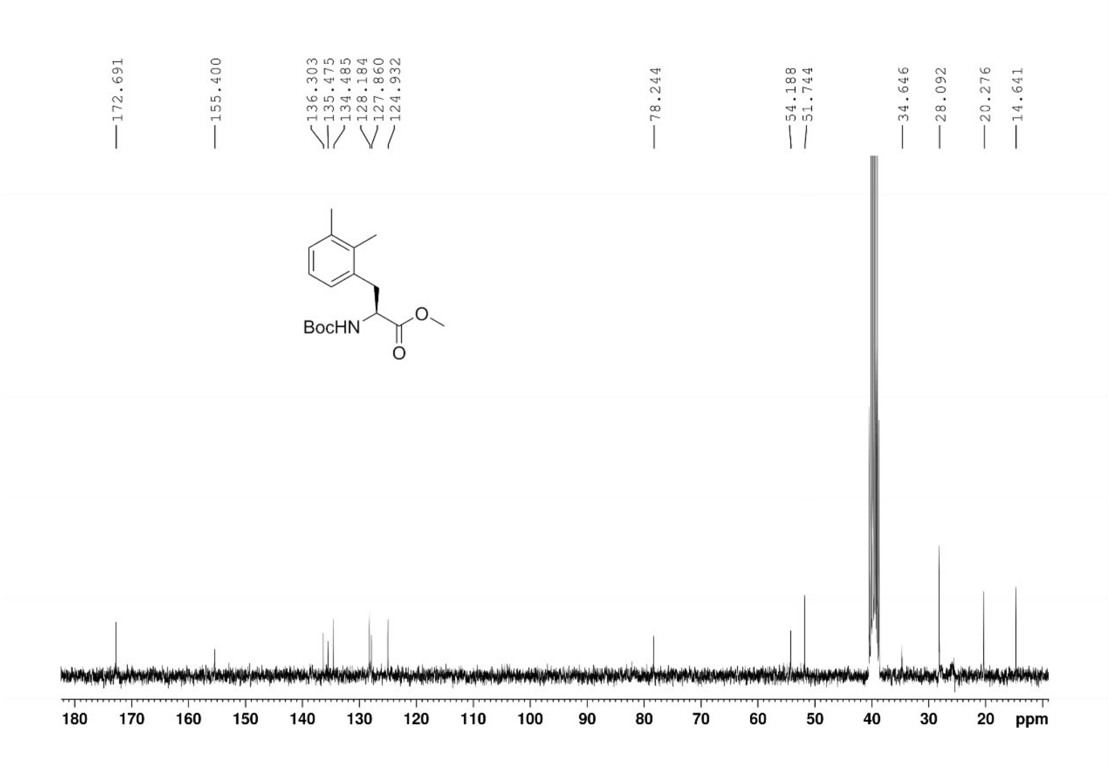

1H-NMR of S10

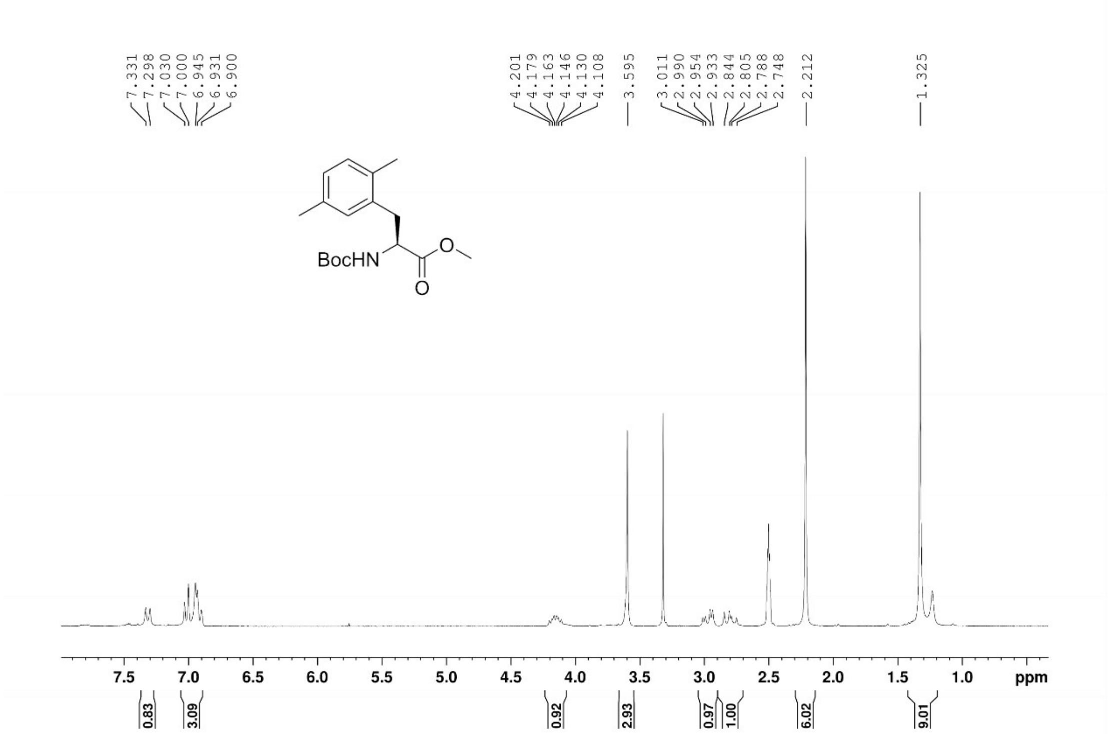

13C-NMR of S10

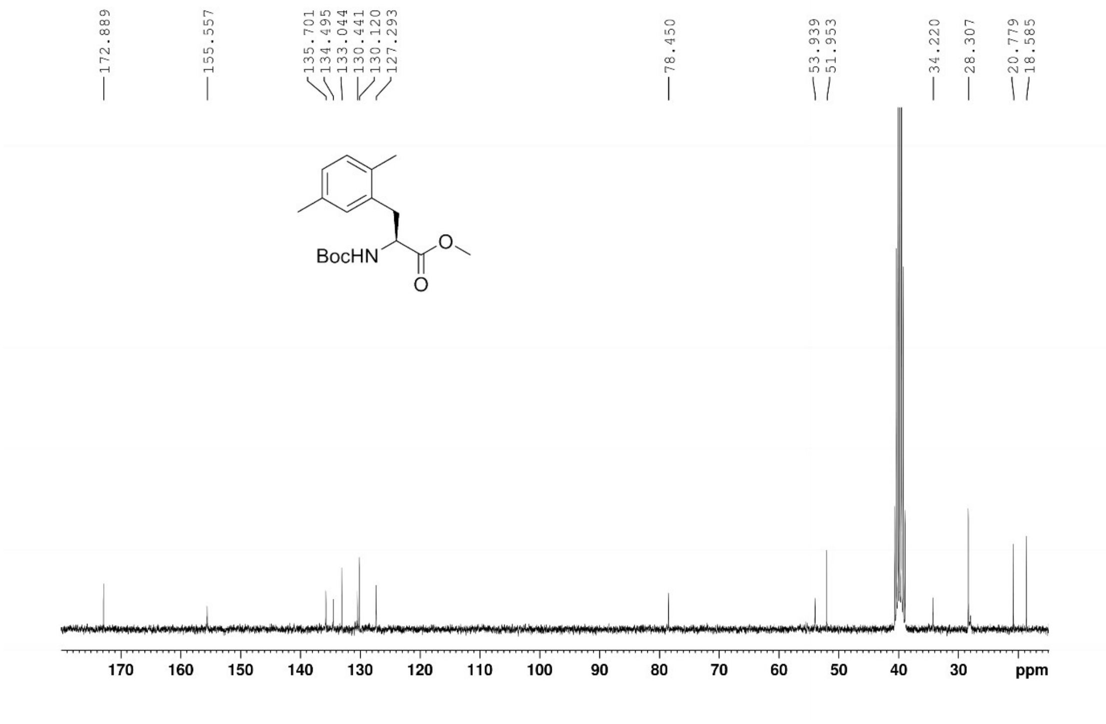

1H-NMR of S12

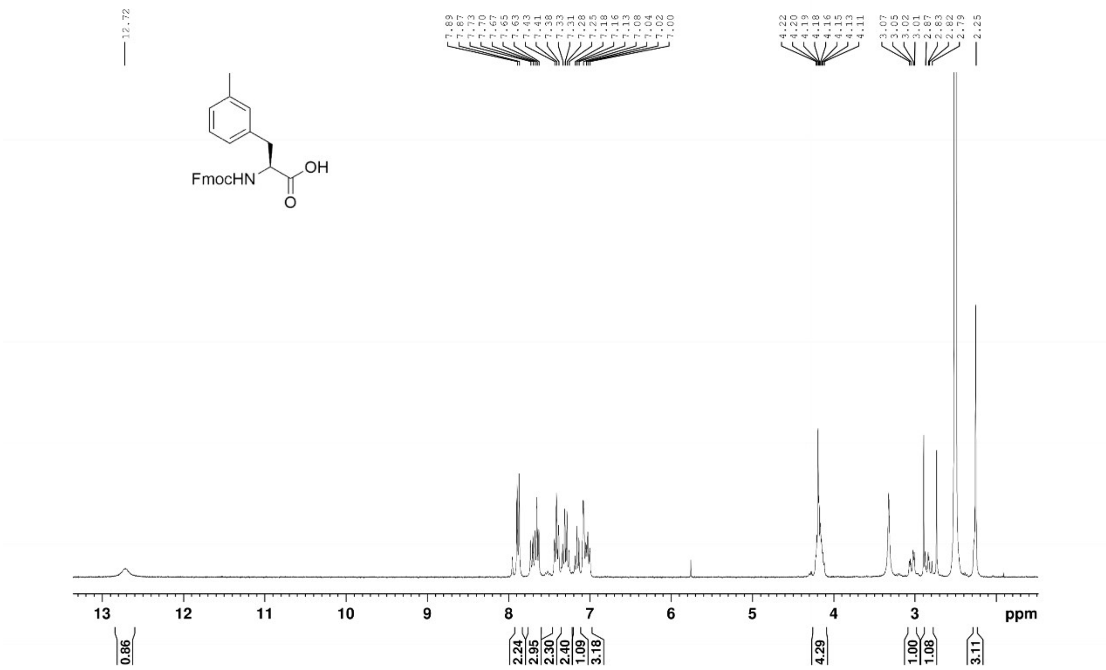

13C-NMR of S12

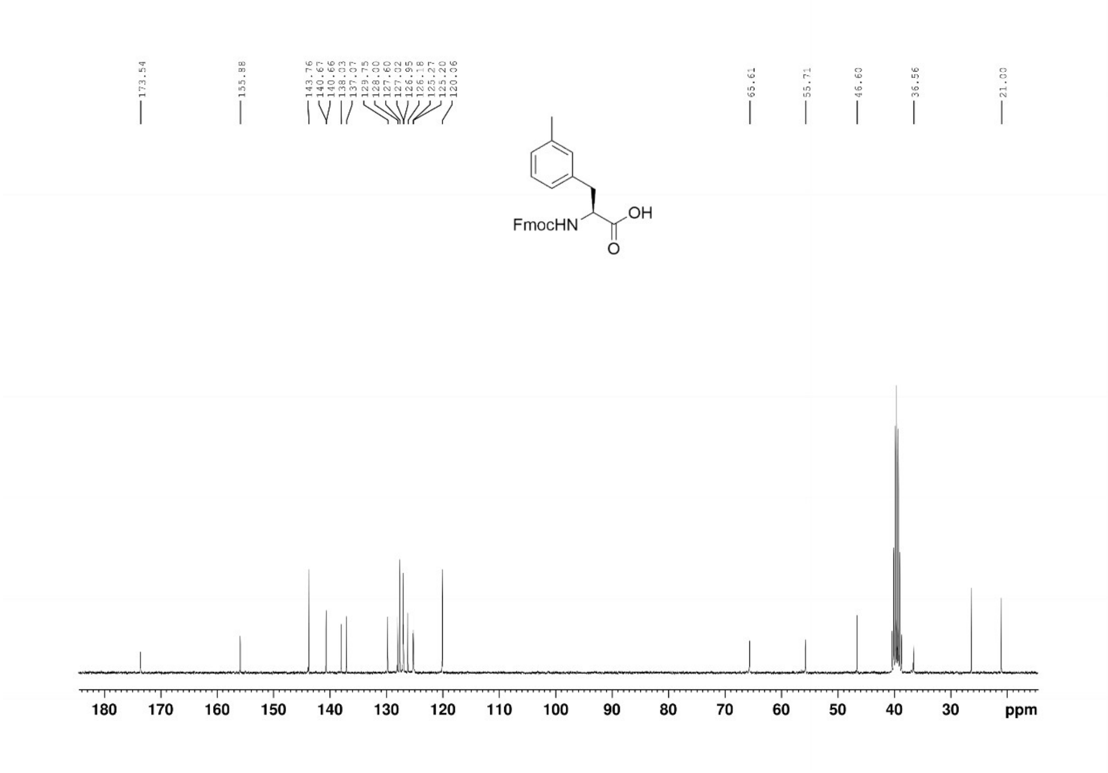

1H-NMR of S13

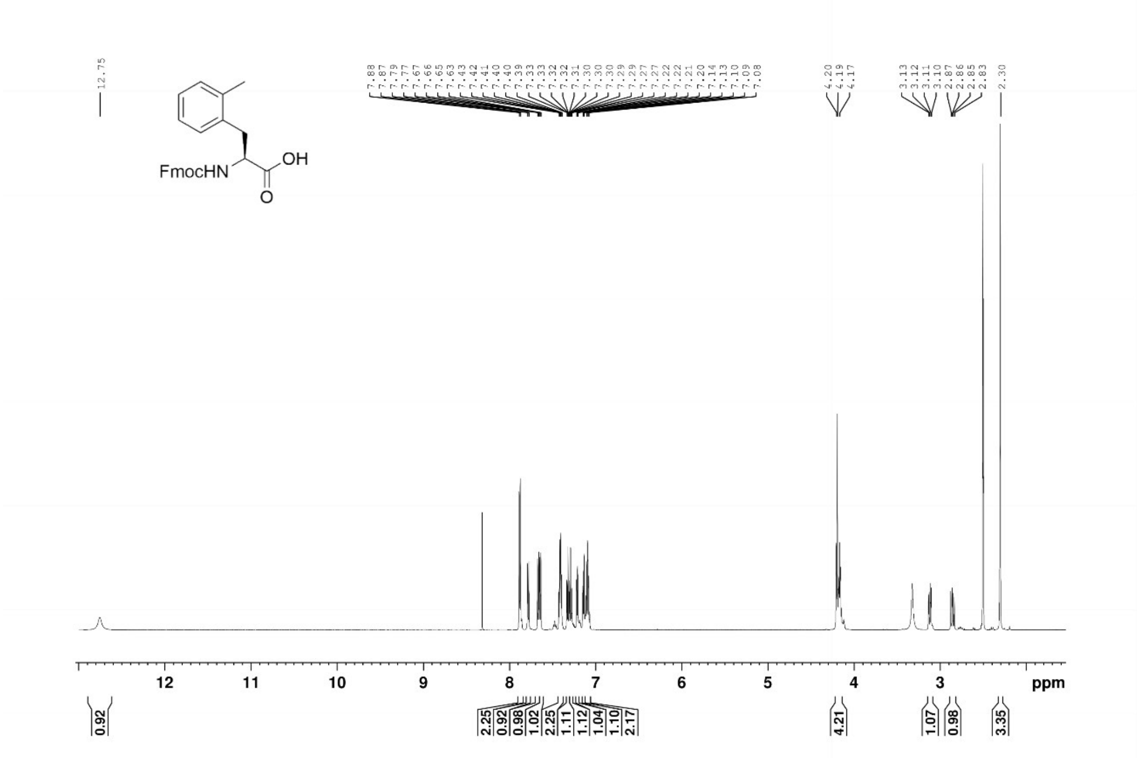

13C-NMR of S13

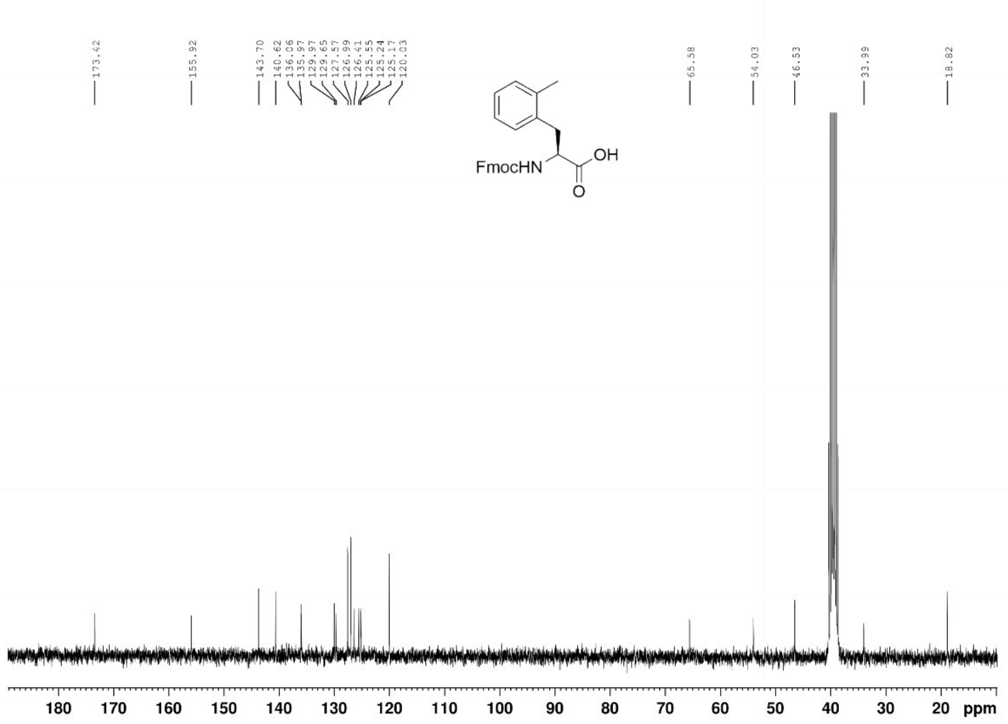

1H-NMR of S14

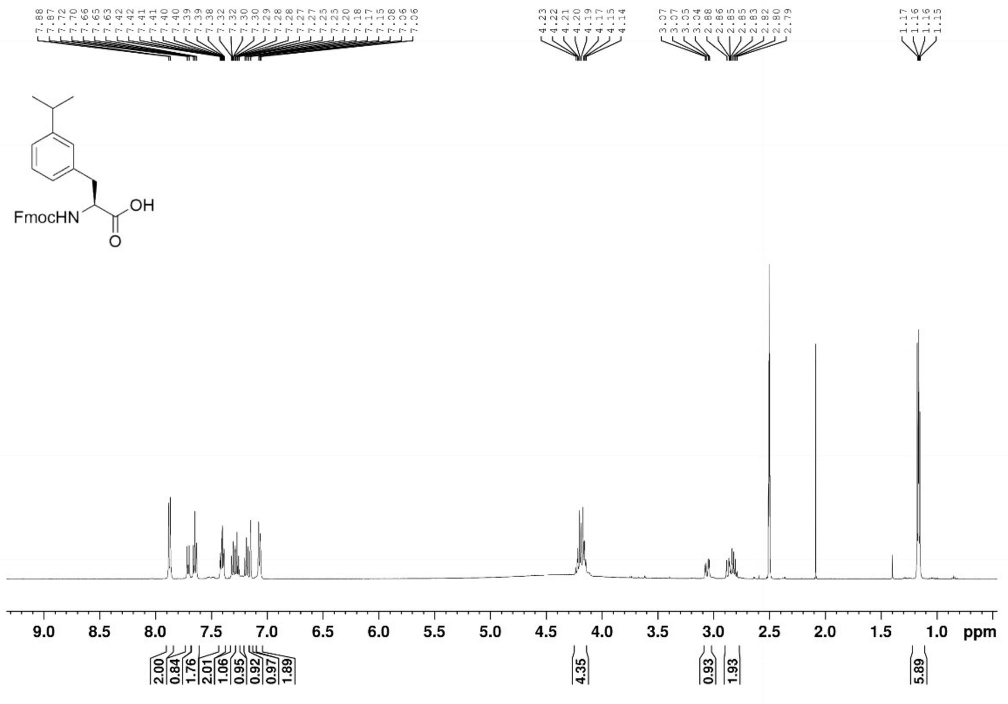

13C-NMR of S14

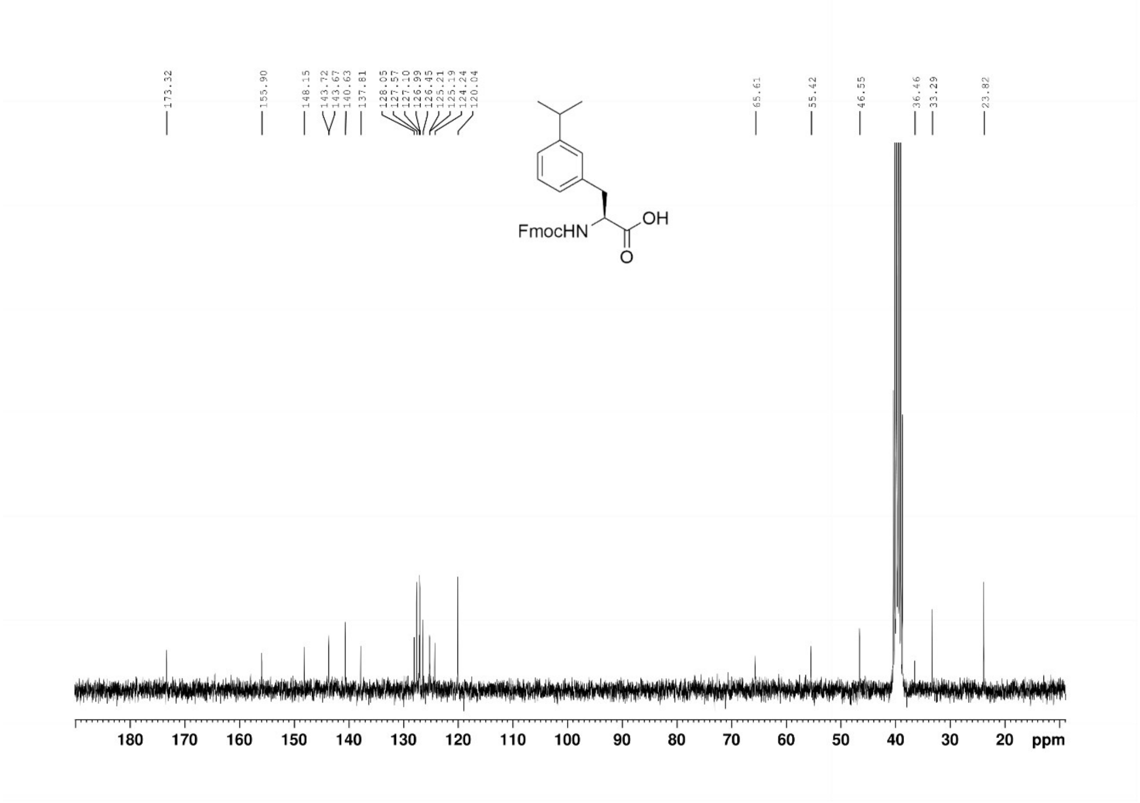

1H-NMR of S15

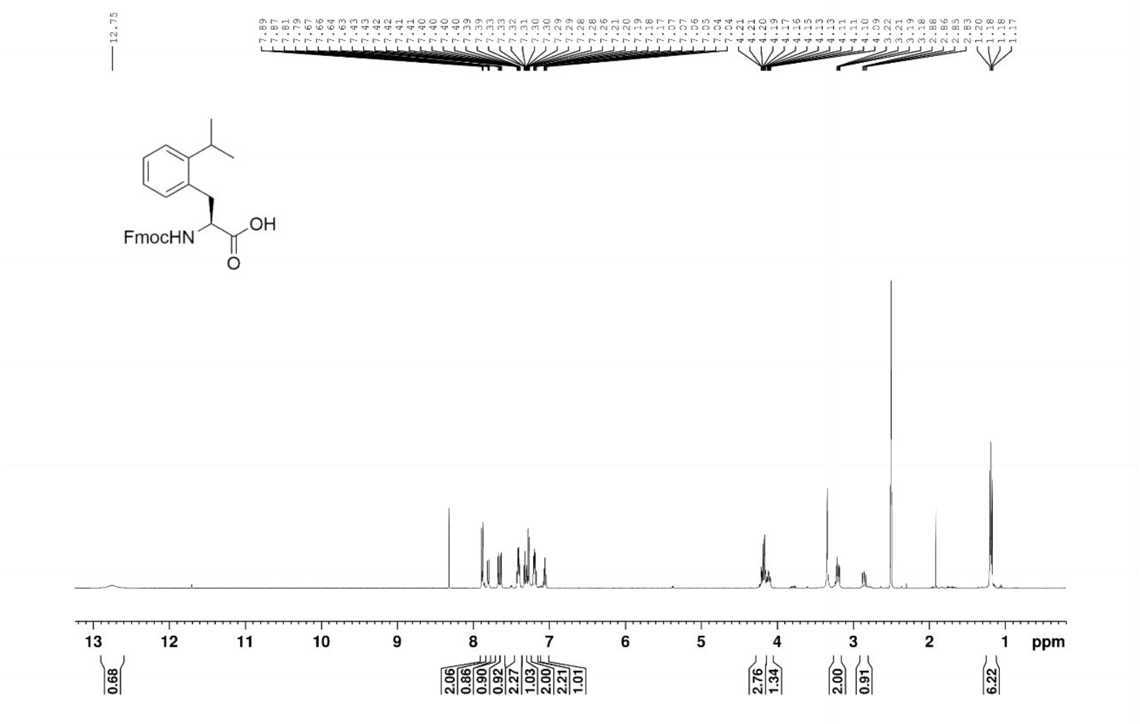

13C-NMR of S15

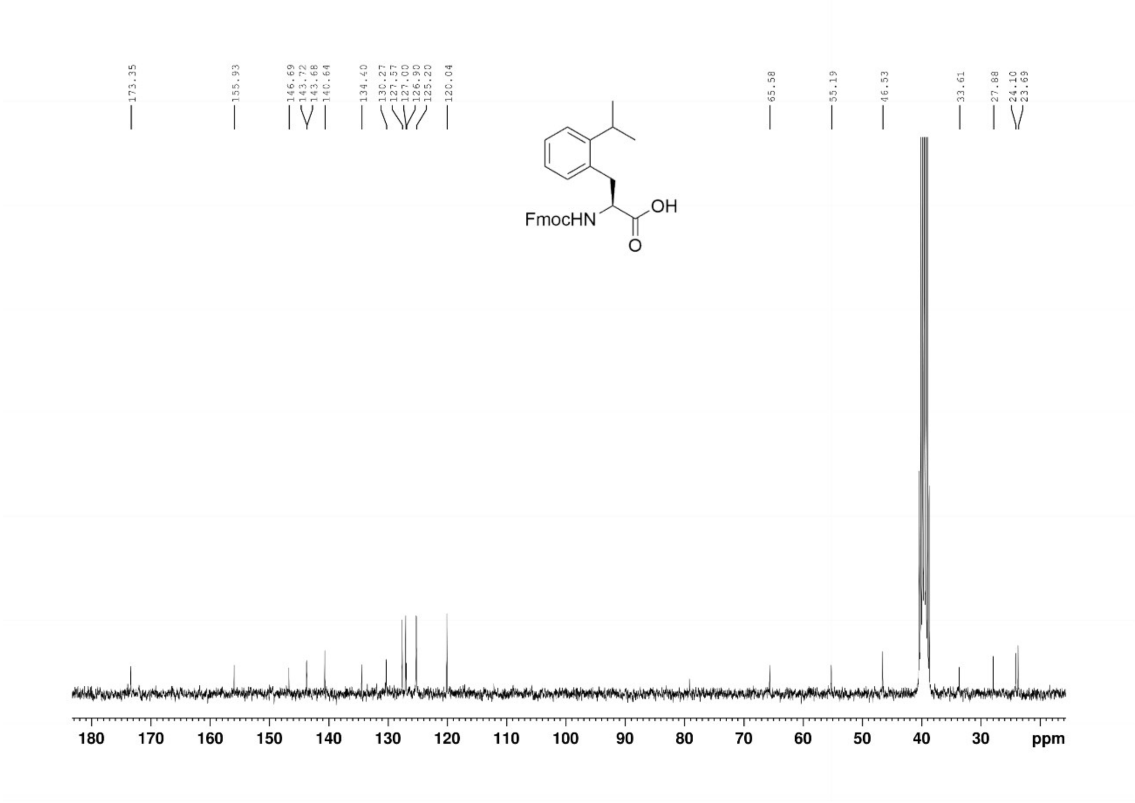

1H-NMR of S16

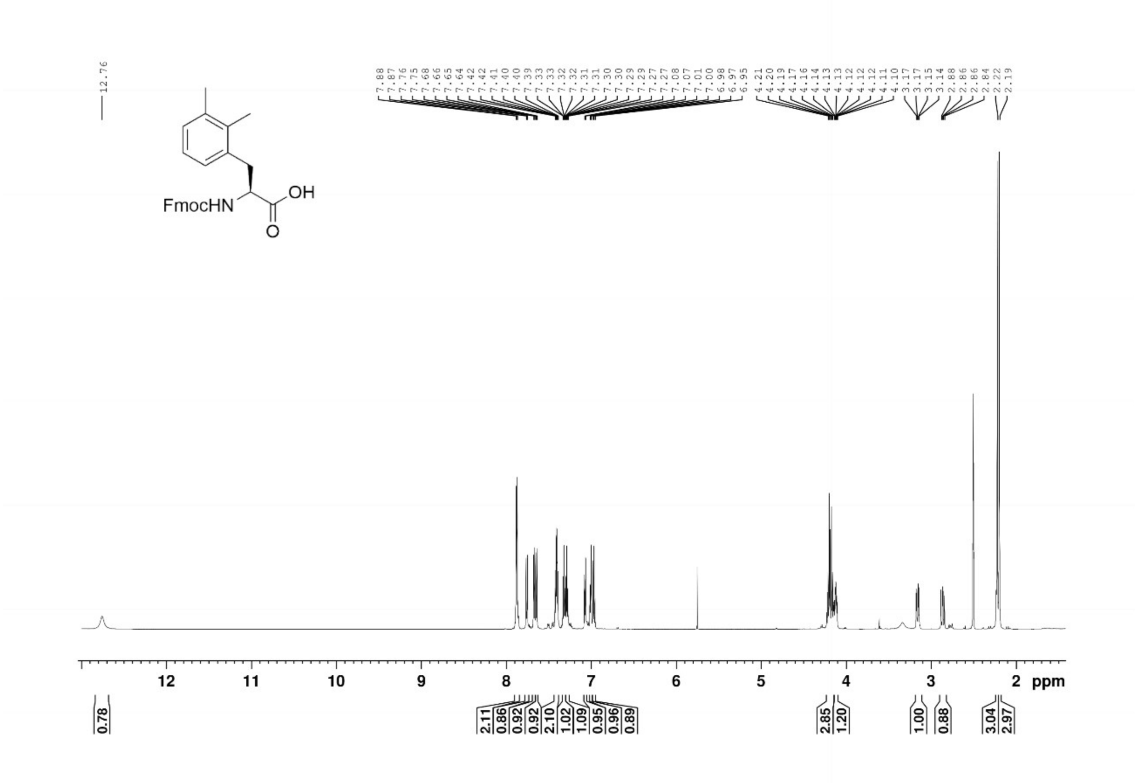

13C-NMR of S16

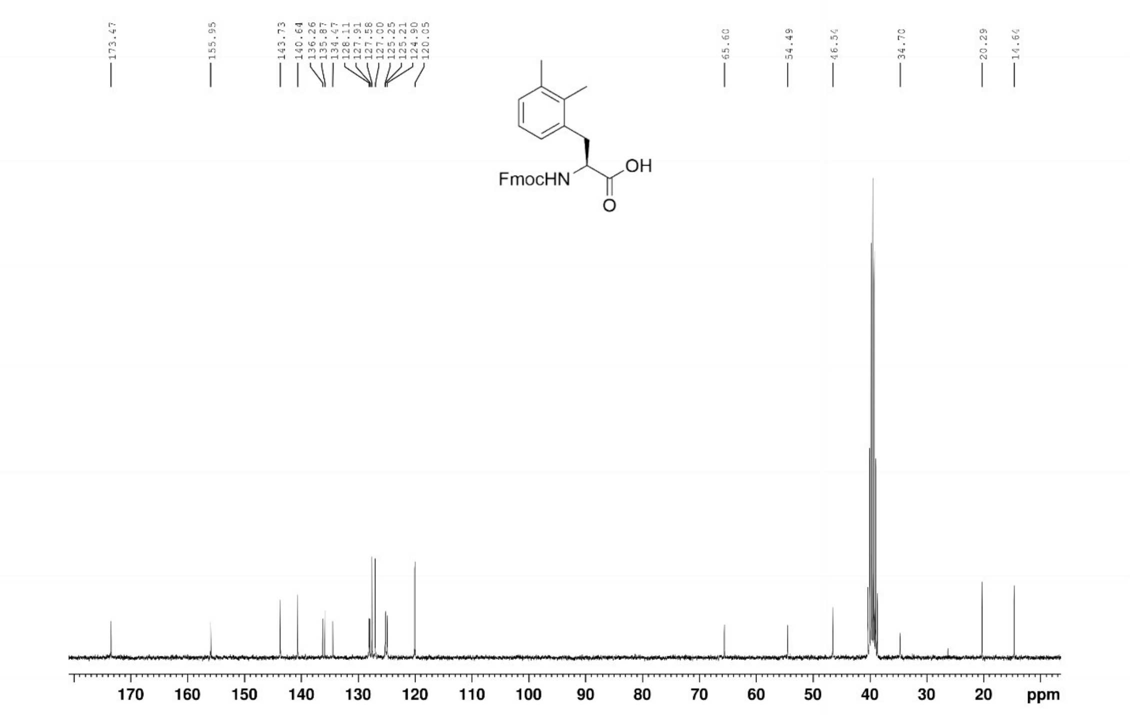

1H-NMR of S17

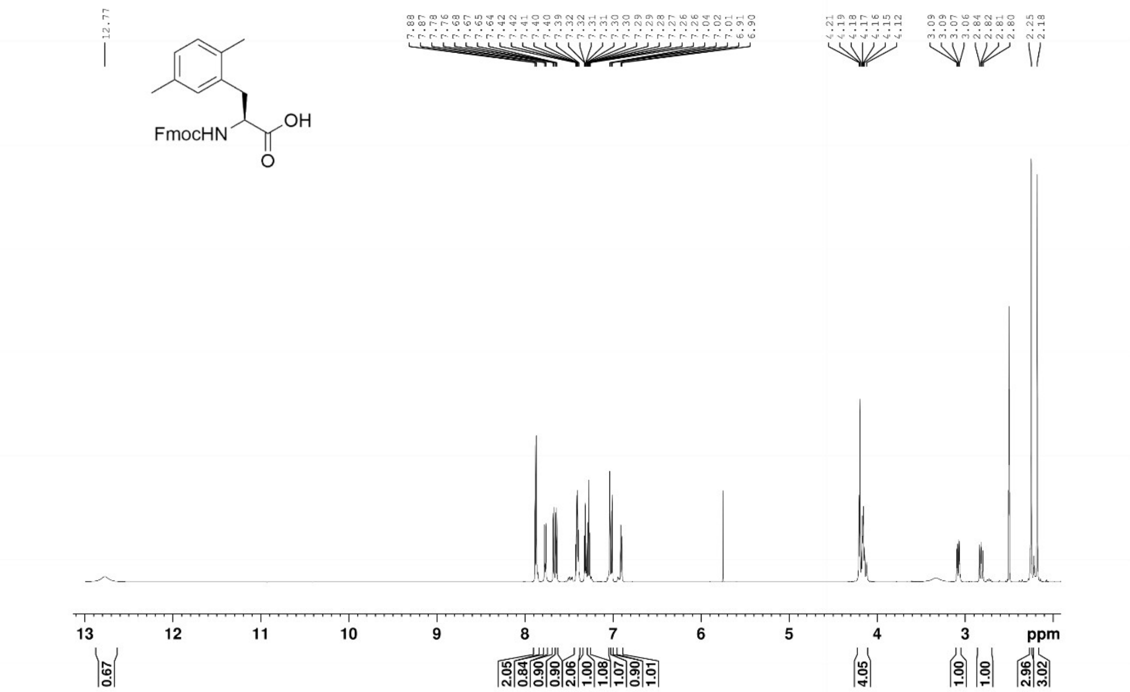

13C-NMR of S17

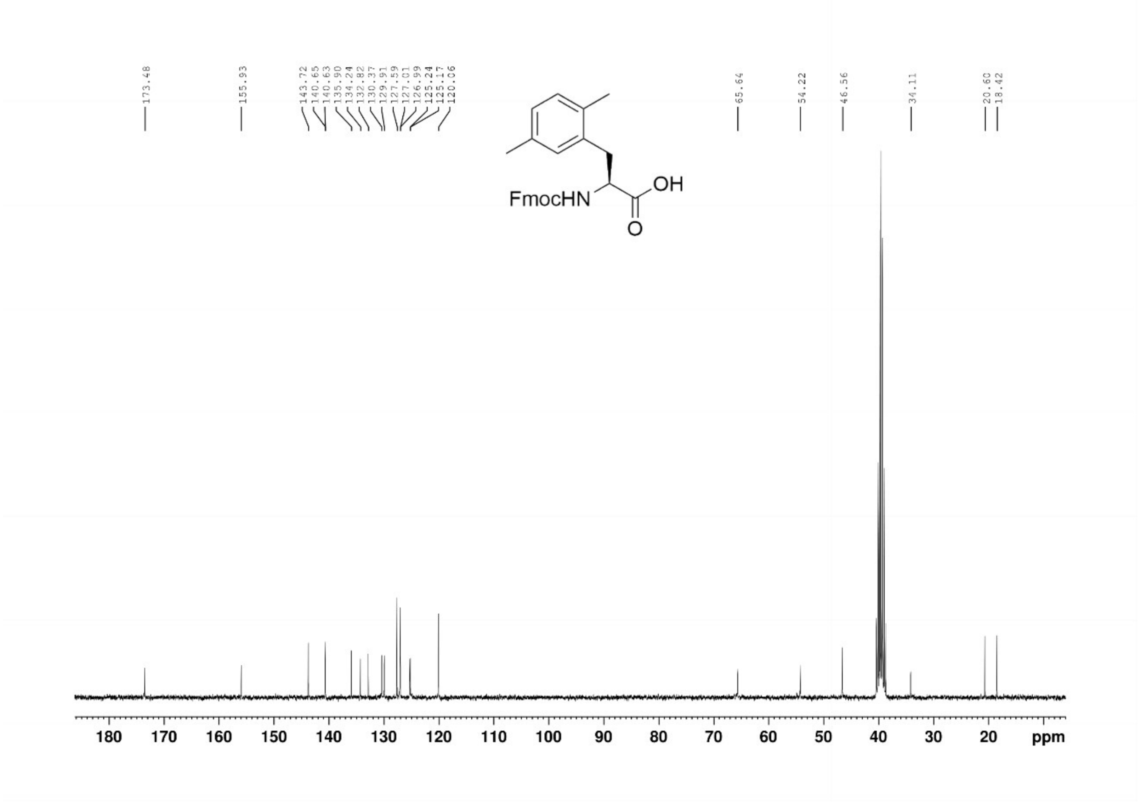

References

1 C. D. Schwieters, J. J. Kuszewski, N. Tjandra and G. Marius Clore, *Journal of Magnetic Resonance*, 2003, **160**, 65.

2 W. Lee, M. Tonelli and J. L. Markley, *Bioinformatics*, 2015, **31**, 1325.

3 E. G. Stein, L. M. Rice and A. T. Brünger, *Journal of Magnetic Resonance*, 1997, **124**, 154.

4 Y. Tian, C. D. Schwieters, S. J. Opella and F. M. Marassi, *J Biomol NMR*, 2017, **67**, 35.

5 E. F. Pettersen, T. D. Goddard, C. C. Huang, E. C. Meng, G. S. Couch, T. I. Croll, J. H. Morris and T. E. Ferrin, *Protein Science*, 2021, **30**, 70.

6 S. Jo, T. Kim, V. G. Iyer and W. Im, *Journal of computational chemistry*, 2008, **29**, 1859.

7 S. Kim, J. Lee, S. Jo, C. L. Brooks, H. S. Lee and W. Im, *Journal of computational chemistry*, 2017, **38**, 1879.

8 H. Berendsen, D. van der Spoel and R. van Drunen, *Computer Physics Communications*, 1995, **91**, 43.

9 M. J. Abraham, T. Murtola, R. Schulz, S. Páll, J. C. Smith, B. Hess and E. Lindahl, *SoftwareX*, 2015, **1-2**, 19.

10 S. Markidis and E. Laure, eds., *Solving software challenges for exascale. International Conference on Exascale Applications and Software, EASC 2014, Stockholm, Sweden, April 2 - 3, 2014 ; revised selected papers*, Springer, Cham, 2015, vol. 8759.

11 S. Páll, M. J. Abraham, C. Kutzner, B. Hess and E. Lindahl, in *Solving software challenges for exascale. International Conference on Exascale Applications and Software, EASC 2014, Stockholm, Sweden, April 2 - 3, 2014 ; revised selected papers*, ed. S. Markidis and E. Laure, Springer, Cham, 2015, pp. 3–27.

12 G. E. Marlow, J. S. Perkyns and B. M. Pettitt, *Chem. Rev.*, 1993, **93**, 2503.

13 G. T. Ibragimova and R. C. Wade, *Biophysical Journal*, 1998, **74**, 2906.

14 J. Zuegg and J. E. Gready, *Biochemistry*, 1999, **38**, 13862.

15 G. D'Abrosca, A. Paladino, I. Baglivo, L. Russo, M. Sassano, R. Grazioso, R. Iacovino, L. Pirone, E. M. Pedone, P. V. Pedone, C. Isernia, R. Fattorusso and G. Malgieri, *Scientific reports*, 2020, **10**, 9283.

16 M. Kashyap, A. K. Ganguly and N. S. Bhavesh, *Scientific reports*, 2015, **5**, 17298.

17 J. Hu, Z. Hu, Y. Zhang, X. Gou, Y. Mu, L. Wang and X.-Q. Xie, *J Mol Model*, 2016, **22**, 156.

18 U. Essmann, L. Perera, M. L. Berkowitz, T. Darden, H. Lee and L. G. Pedersen, *The Journal of Chemical Physics*, 1995, **103**, 8577.

19 B. Hess, H. Bekker, H. Berendsen and J. Fraaije, *Journal of computational chemistry*, 1997, 1463.

20 A. W. Da Sousa Silva and W. F. Vranken, *BMC Res Notes*, 2012, **5**, 367.

21 G. A. Tribello, M. Bonomi, D. Branduardi, C. Camilloni and G. Bussi, *Computer Physics Communications*, 2014, **185**, 604.

22 Eckhardt, W. Grosse, L.-O. Essen and A. Geyer, *PNAS*, 2010, **107**, 18336.
